# Statistics of seismicity to investigate the Campi Flegrei caldera unrest

**DOI:** 10.1038/s41598-021-86506-6

**Published:** 2021-03-30

**Authors:** A. Tramelli, C. Godano, P. Ricciolino, F. Giudicepietro, S. Caliro, M. Orazi, P. De Martino, G. Chiodini

**Affiliations:** 1 Istituto Nazionale di Geofisica e Vulcanologia, Osservatorio Vesuviano, Napoli, Italy; 2 Department of Mathematics and Physics, University of Campania “Luigi Vanvitelli”, Caserta, Italy

**Keywords:** Natural hazards, Solid Earth sciences

## Abstract

The knowledge of the dynamic of the Campi Flegrei calderic system is a primary goal to mitigate the volcanic risk in one of the most densely populated volcanic areas in the world. From 1950 to 1990 Campi Flegrei suffered three bradyseismic crises with a total uplift of 4.3 m. After 20 years of subsidence, the uplift started again in 2005 accompained by a low increment of the seismicity rate. In 2012 an increment in the seismic energy release and a variation in the gas composition of the fumaroles of Solfatara (in the central area of the caldera) were recorded. Since then, a slow and progressive increase in phenomena continued until today. We analyze the INGV - Osservatorio Vesuviano seismic catalogue of Campi Flegrei from 2000 to 2020 in order to look for any variation in the seismic parameters and compare them with geochemical monitored ones. A remarkable correlation between independent variables of earthquake cumulative number, CO/CO_2_ values and vertical ground deformation reveals a likely common origin. Moreover the correlation between all the variables here analysed enlightens that the same origin can cause the temporal behavior of all these variables. We interpret the seismological, geochemical and geodetic observable in terms of the injection of magmatic fluids into the hydrothermal system or its pressurization.

## Introduction

Within the bradyseismic phenomenon which characterize the Campi Flegrei volcanic field, an uplift of the central part of the caldera has been recorded since 2005, reaching a value of 65 cm at the beginning of 2020. The last eruption of Campi Flegrei occurred in 1538 on the western side of a raised marine terrace, and, on the basis of historical and geological reconstructions, the seismicity and uplift had been increasing for about 100 years before the eruption. Some days before the eruption occurred a rapid and localized increase of the ground uplift^1,2^. A slow regular and persistent subsidence occurred for most of the period since the last eruption. Uplift episodes are documented in 1950–1952, 1969–1972 and 1982–1984^3^. The last one was associated with a total uplift of 1.8 m in the central area of the caldera with the occurrence of more than 16,000 earthquakes^4^ without culminating in an eruption.

**Figure 1:**
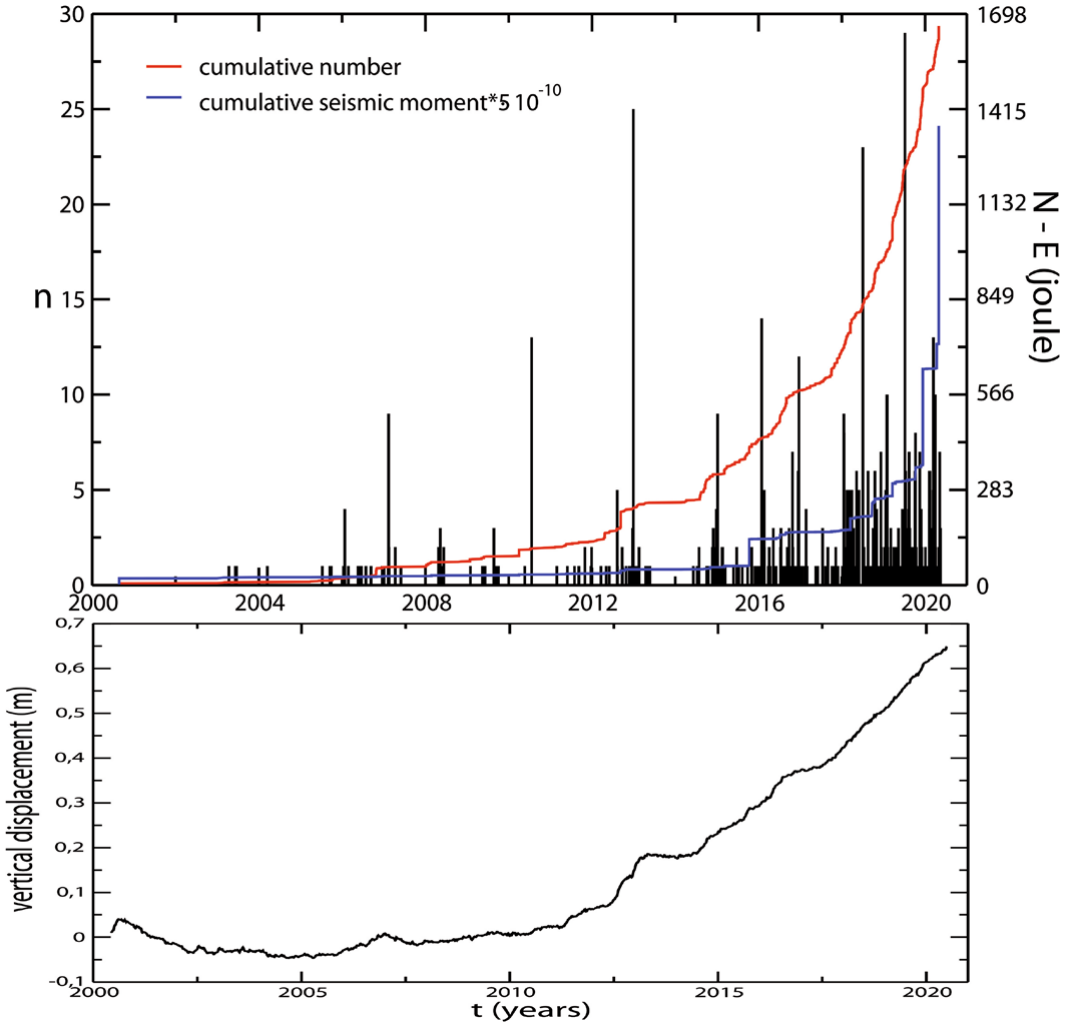
Top panel: histogram of the earthquakes with Mc ≥ 0.2 per day (black); cumulative of the earthquakes number (red) and cumulative of the energy released (blue) of the whole catalogue. Bottom panel: Vertical daily displacement at RITE (Rione Terra, Pozzuoli) GPS station from 29 May 2020 to 30 June 2020.

After a subsidence of more than 20 years, the caldera uplift started again in 2005^5^ together with an increment of the seismicity rate^6^ and of the degassing activity^7^. An increment in the seismic energy released occurred in 2012 (see Fig. 1). After a brief period of quiet, uplift resumed in 2014, the seismicity become shallower (< 2 km) and its rate increased again^8,9^.

Uplift, seismicity and gas-composition of the hydrothermal system are showing variations that needs to be studied to understand a possible evolution of the volcanic system. We statistically analyze the seismic catalogue of Campi Flegrei from 2000 to 2020 to understand the temporal variations of inter-time and inter-space between earthquakes and to correlate them with the trend of the geochemical parameters and deformations.

## Method

We analyzed the seismic catalogue of Campi Flegrei which is constantly updated from INGV - Osservatorio Vesuviano. We considered the time window from August 2000 to April 2020. The catalogue contains earthquake time, location, *M*_*d*_ and location errors. Each event is detected and located automatically or, eventually, by the shift workers present in the monitoring room of Osservatorio Vesuviano 24 H/day. Successively, each earthquake is manually revised by the seismic laboratory, located with the program Hypo71^10^ in a layered velocity model optimized for the caldera (Table 1) and inserted into the official catalogue here considered. The mean location error of the earthquakes present in the catalogue is less than 350 m for the horizontal and less than 400 m for the vertical position.

**Table 1:** Seismic velocity model for the Campi Flegrei caldera used by the seismic laboratory of INGV - Osservatorio Vesuviano, the Vp/Vs is 1.78 (^4^ and reference there in).

1D velocity model of Campi Flegrei	
Velocity of the P-waves (km/s)	Depth of the top of the layer (km b.s.l.)
1.7	0.00
2.0	0.50
3.0	1.00
5.5	3.00
5.7	10.00

The magnitude of the Campi Flegrei seismic catalogue is evaluated by the coda duration measured at a short-period seismic station located at the Solfatara crater (STH). The duration-magnitude empirical relation can be found in^4^. The same relationship and station have been used since 1984 ensuring a stable and stationary method for magnitude estimation.

### The theoretical sensibility of the seismic network with time

The detection threshold of any seismic network can be characterized by the completeness magnitude *M*_*c*_. For magnitudes larger than *M*_*c*_ the network is able to detect any earthquake occurring in the area, conversely, not all the events with magnitude smaller than *M*_*c*_ are detected. As a consequence, the number of earthquakes with a magnitude smaller than *M*_*c*_ reported in the catalogues is smaller than that expected by the Gutenberg–Richter relationship.

**Figure 2:**
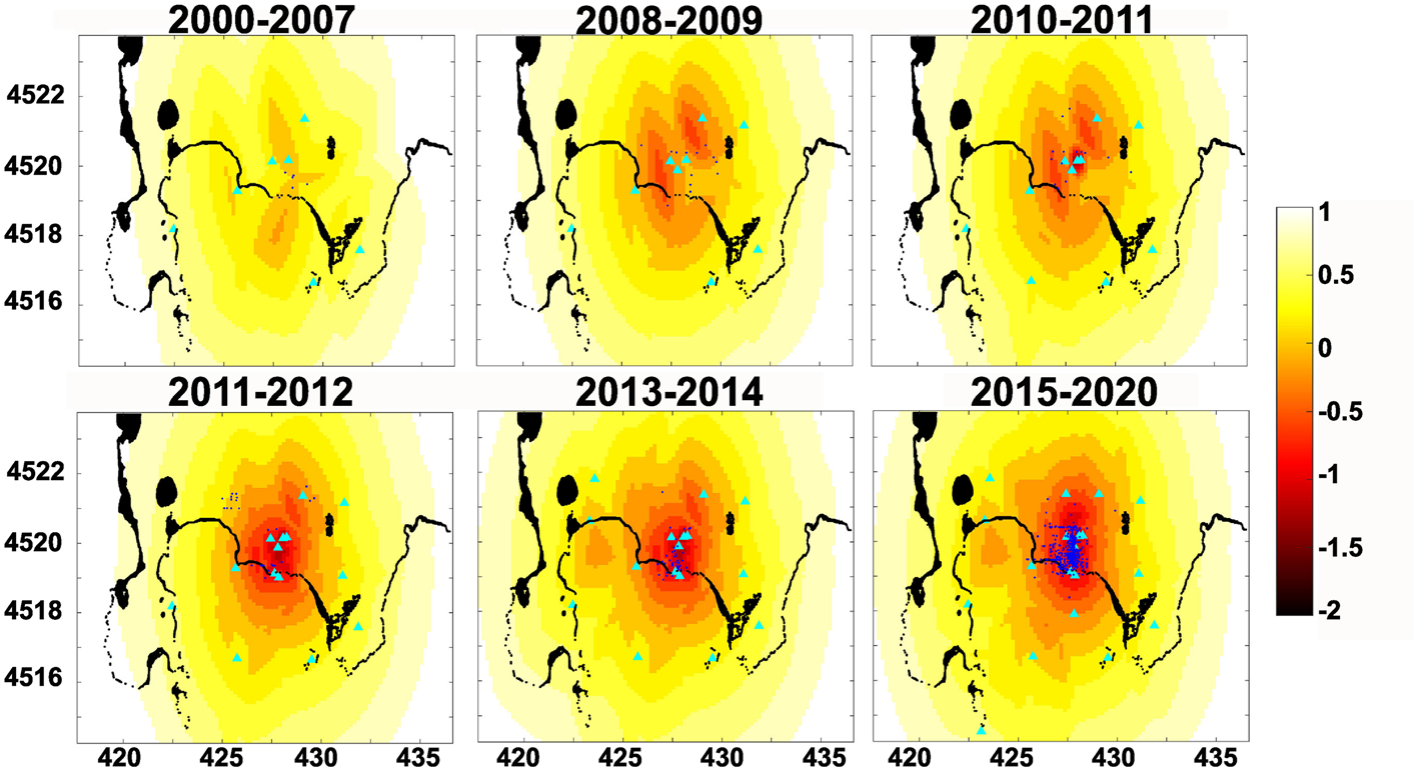
Temporal variations of the sensibility to location of the seismic network. Seismic stations are indicated by cyan triangles and earthquakes with Md ≤ 0.2 are shown by blue dots. The colorbar indicates the magnitude threshold of location. Map coordinates are km UTM.

Analyzing a seismic catalogue *M*_*c*_ is usually estimated with different techniques: the maximum curvature method^11^, Goodness-of-Fit Test^11^, *b*-value stability approach^12^, Entire Magnitude Range^13,14^, and others. To take into account the improvement of the Campi Flegrei seismic network with time we used a theoretical method that allows to define the detection and location sensibility in space of a seismic network^15,16^ and apply it for different time windows. The method consists in simulating earthquakes with different magnitudes distributed on a grid at a certain depth. More precisely, a grid of synthetic earthquakes, located in the area plotted in Fig. 2 at a depth of 1 km b.s.l., has been generated with the following source and medium parameters: stress drop of 0.5 MPa (using the^17^ source model), average rock density of 2000 kg/m^3^^18^ mean quality factor Q=100^19^. The seismic noise at each station has been evaluated as the mean daily noise. The magnitude threshold for location is defined as the minimum magnitude of an earthquake recordable at least by 4 seismic stations. The same method is used for the detection threshold but the number of stations recording the earthquakes has to be larger than 1.

As we are considering the seismic catalogue of the located earthquakes we assume that the location sensitivity of the network in the epicentral area represents a theoretical evaluation of *Mc*. For the analysis we considered the time periods indicated in Fig. 2 and the seismic stations operating for most of the referring period.

Figure 2 shows that the number of seismic stations increased with time (cyan triangles) and the network performances has been improved in parallel enlarging the location area. To allow a better understanding of the proposed image we also plot the earthquakes with magnitude smaller than 0.2 recorded in the referring period. This analysis allows us to define a *M*_*c*_ range between 0 and 0.5 in the inner part of the caldera for the whole analysed period.

### The temporal variations of *b* and the empirical evaluation of *M*_*c*_

**Figure 3:**
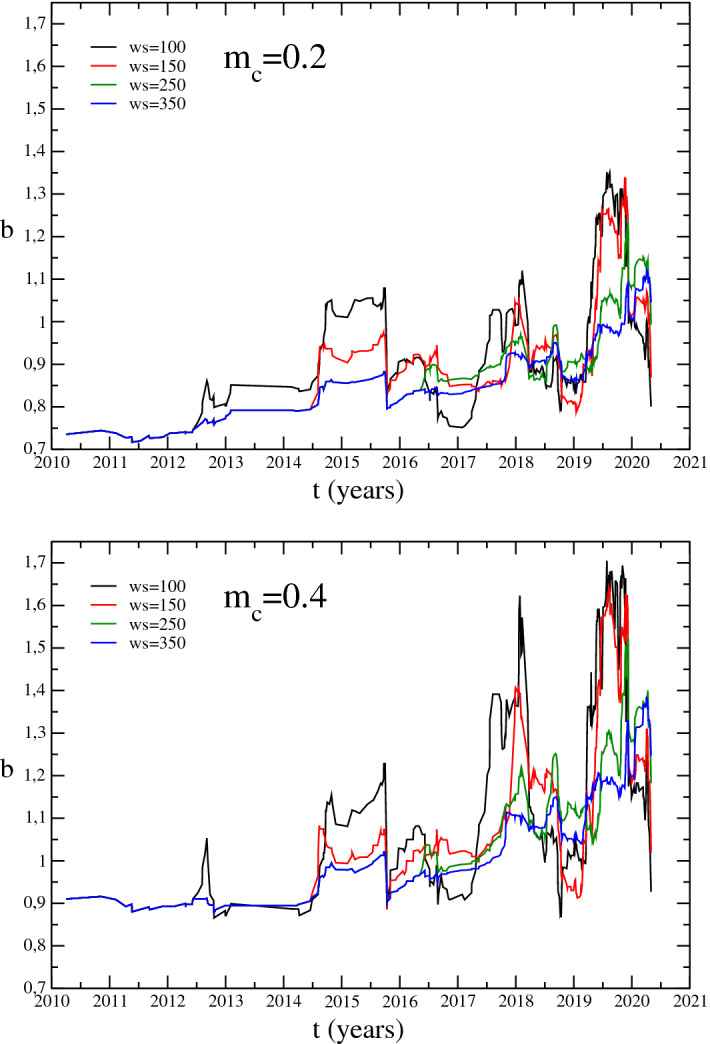
Temporal variations of the *b* value for different window size and *M*_*c*_ values.

As a first step we estimate the temporal variations of the Gutenberg–Richter *b* parameter. The analysis has been performed dividing the catalogue in moving time windows. In order to analyse the influence of the window on the *b* value estimation we used, as suggested by^20^, different window lengths, going from 100 to 350 earthquakes. As can be seen in Fig. 3 the *b* value tends to assume smaller values for larger window size and shows higher variations for smaller window sizes. Our main interest is in these variations to be compared to the trend of other variables. As a consequence we decide to use a window of 150 events representing a good compromise. Moreover the simulation, described in the Supplementary Materials, reveals that using 150 events it is possible to obtain a reliable estimation of the *b* value. Due to the small occurrence rate of earthquake in the first part of the analyzed catalogue, taking a window length of 100 earthquakes the first sample point with magnitude above 0.2 occurs in 2010. With a window length of 350 earthquakes the first sample occurs in 2016. Using 150 samples the analysis starts from 2014.

For each time window we estimate the *M*_*c*_ and the *b* value minimizing the residuals of the Gutenberg–Richter fit^12^. More precisely we analyse the residuals associated with the *b* value in respect to the *M*_*c*_ variations. The obtained *M*_*c*_ values span from − 0.2 to 0.4, with some points at 0.8. However the *M*_*c*_ variation does not exhibit any trend enlightening that there is no evidence of a visible improvement of the *M*_*c*_ estimates in the epicentral area since 2012. From the sensibility analysis we notice that after 2011 the sensibility location threshold of the central part of the caldera remain almost constant, confirming the empirical data. Moreover we observe that, if we use two different values of the completeness magnitude, namely *M*_*c*_ = 0.2 and *M*_*c*_ = 0.4, the *b* parameter variation trend does not exhibit any substantial difference. Even if the *b* value assumes smaller values for *M*_*c*_ = 0.2 in respect of *M*_*c*_ = 0.4, the trend and the variation points are the same (Fig. 3). Finally, we decided to fix *M*_*c*_ = 0.2 for any analyzed window. The reasons for this choice are deeply discussed in the Supplementary Materials.

To evidence possible different behavior of the seismogenic structures, in addition to the *b* value, we evaluate the average intertime Δ*t* between two successive earthquakes, the average epicentral interdistance *ξ* between all the possible couple of the 150 earthquakes weighted for the location horizontal error and the average depth *ζ* weighted for the vertical location error.

The *b* values are estimated using the maximum likelihood method^21^ and their errors are estimated using the^22^ formula. The error in the *b* value estimation due to the use of the *M*_*d*_ has been extensively described in the Supplementary Material and is almost equivalent to the one estimated using^22^.

**Figure 4:**
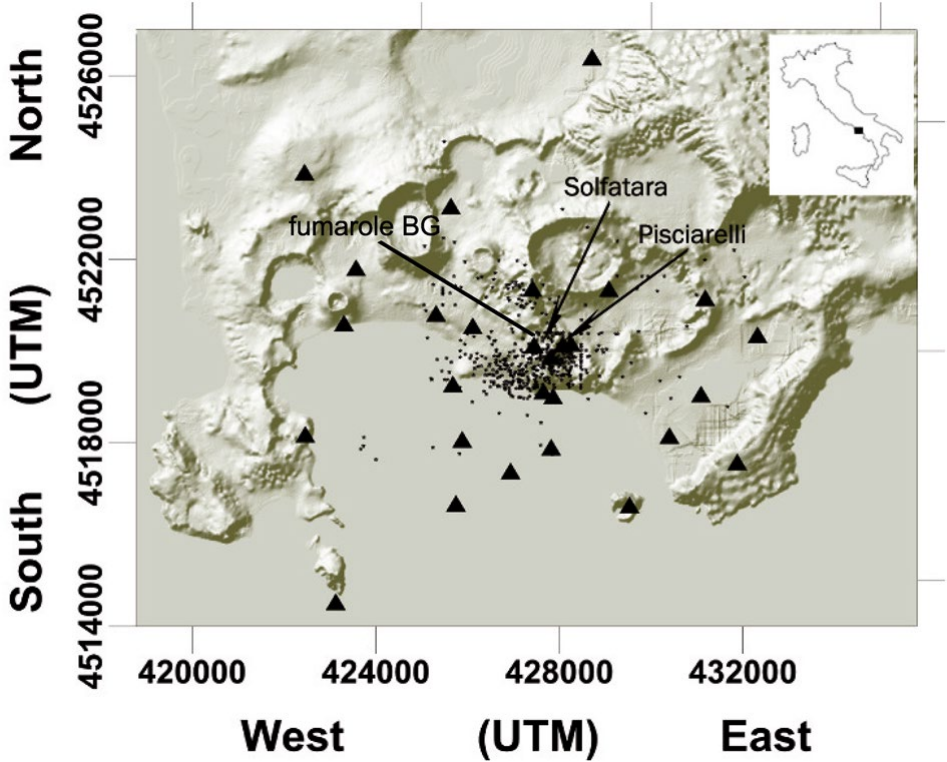
Map of the Campi Flegrei with seismic stations (triangles) and earthquakes (little stars) positions. Pisciarelli and Solfatara vents are indicated with arrows. The map is produced with Surfer v20, https://www.goldensoftware.com/products/surfer.

The obtained seismological time series are finally compared with independent compositional data of the main fumarolic emission of Campi Flegrei (i.e. “Bocca Grande” fumarole, BG in Fig. 4) that showed important variations interpreted as due to heating and pressurization of the feeding hydrothermal system^7–9^. Among the many monitored parameters, we will refer in particular to the CO/CO_2_ fumarolic ratio that is the best indicator of the ongoing heating process (^7^ and references there in).

## Results

The analysis of the *M*_*c*_ shows that its value remains almost constant in the last 10 years. Despite the network has been improved, the new stations were deployed mainly in the western border of the caldera and in its sea side enlarging the area covered by the network and allowing to evidence a possible hypocentral migration and to improve the location error. Otherwise the area where the seismicity mainly occurs is close to the Solfatara crater (Fig. 4) that has been well covered by the network since 2010. Except for some isolated earthquakes, the seismicity is indeed occurring in the central part of the caldera close to the Solfatara crater; differently from what recorded during the 1982–1984 bradyseismic crises when earthquakes were scattered within the whole caldera.

In 2005 a new uplift started in Campi Flegrei caldera together with an increment in the seismicity and a variation in the gas composition emitted at the Solfatara fumarolic vents. The uplift has a bell shape centered in the town of Pozzuoli (Rione Terra) where the elevation reached 65 cm at the beginning of 2020. The uplift started slowly, 1–2 cm per year, until 2012 when the uplift rate increased to 6–7 cm per year. The temporal evolution of these phenomena, together with the variations in the gas composition of the monitored fumaroles, lead the Civil Defence of Italy to move the alert state of the volcano from green to yellow in 2012.

In volcanic areas the seismicity is related to the presence of fluids, to the rheology of the crust, and to the local stress fields^23^. Its increasing rate and energy released, together with surface deformation, and degassing, is used to define caldera unrests^2,24^. Seismicity is usually described using occurrence rate, magnitude and the Gutenberg–Richter *b* value. In volcanic areas the magnitude is usually small to moderate and the *b* value is higher than 1, due to the presence of fluids within a highly fractured rock. Siniscalchi et al.^25^ estimated the *b* value for the Campi Flegrei area during the period 2005–2016. They found a value of 0.92 whereas^9^ found a value of 1.03 for the period 2005–2019. A significantly smaller value (0.72) was found, for the period 1983–1984, by^6^. Such a small value can be related to high stress regimes^26^. It is interesting to notice that, during the 1982–1984 bradiseismic crises the seismicity was spread over the caldera inner and the *b* value was very small ($\simeq$ 0.5–0.6) for earthquakes located in the South-West part of the caldera. Conversely it assumes slightly higher values ($\simeq$ 0.7) for earthquakes located closer to the Solfatara area^6^. From August 2000 to April 2020 about 1700 earthquakes have been recorded. Most of them are located in the Solfatara/Pisciarelli area and we estimated a *b* value of 0.92 ± 0.03 for the whole period. The seismicity is moderate but, taking into account the 20 years of the analyzed catalogue, more than the 70% of the total energy have been released in the period from December 2019 to April 2020 (blue line in Fig. 1).

**Figure 5:**
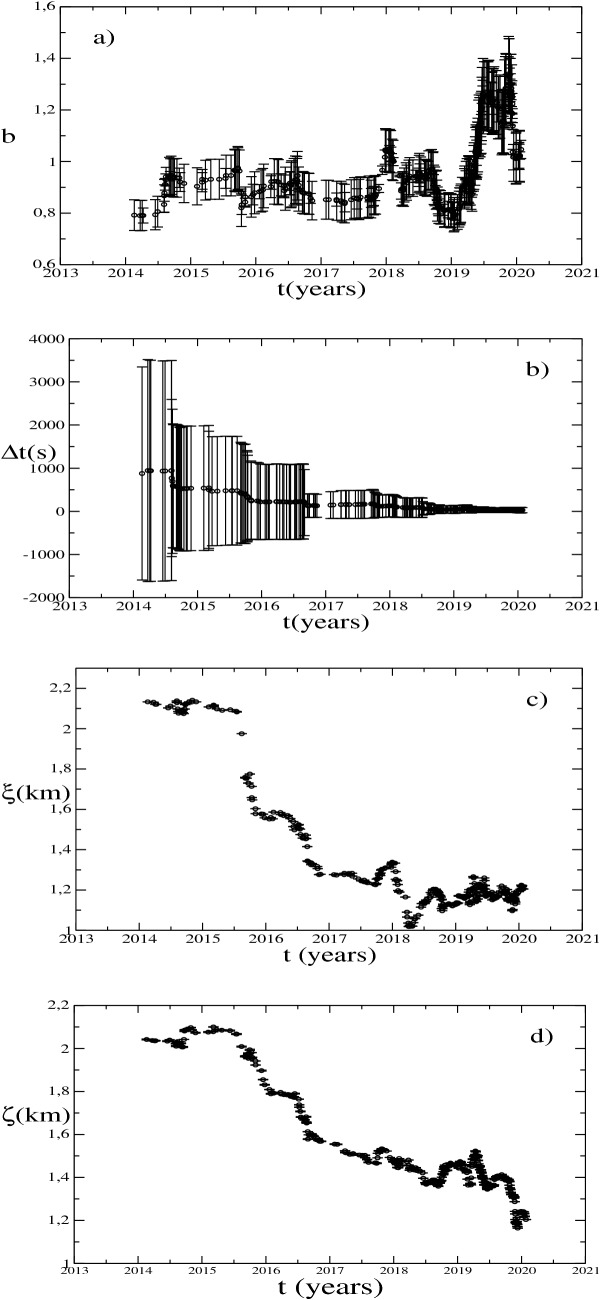
Temporal variation of (**a**) b-value with its standard deviation calculated following^22^; (**b**) inter-time; (**c**) and (**d**) epicentral inter-distance and average depth, respectively. Error bars represent the standard deviations.

The *b* value increases from values smaller than 0.8 in 2014 to values close to 1.3 at the end of 2019 (Fig. 5a). Periods of variability are identifiable at the beginning of 2018 and at the end of 2019 when it again increases reaching values close to 1.3 (Fig. 5a). This increment could be associated to high pore pressure probably induced by magmatic fluids injection^26^. When magmatic fluids intrude, the temperature around them rises weakening the crust which is unable to accumulate high stress. Weakened crusts as well as expanding sills^27,28^ could generate numerous small fractures and, consequently, the *b* value increment.

The evolution of the average intertime between earthquakes (Fig. 5b) evidences that the seismicity is getting closer with time. At the beginning of the analyzed catalogue the average intertimes assumed values of several hours with huge standard deviations. As well as the time goes on, both average values and standard deviations decrease. This reveals that the clustering regime persists with time. Indeed, the clustering degree can be characterized by the *c*_*V*_ defined as $c_V=\frac{\sigma }{\langle \Delta t\rangle }$ being *σ* the standard deviation and $\langle \Delta t\rangle$ the average intertime. *c*_*V*_ assumes a value equal to 0 for periodic occurrence, equal to 1 for Poissonian occurrence and > 1 for clustered events. For the analyzed catalogue *c*_*V*_ ranges from 1.8 to 2.2. A stationary *c*_*V*_ > 1 implies a constant clustering degree even if a decreasing $\langle \Delta t\rangle$ (its value decreases with time till values of several dozens of seconds in 2019) indicates the occurrence of denser swarms separated in time. The inter-space trend, shown in Fig. 5c, enlightens the increment of the earthquake spatial clustering with time. Being the earthquakes in a swarm always close to each others, it shows that the swarms become closer in space. The inter-space goes from values of 2.2 to 1.2 km with a mean error of the epicentral location of a few hundred meters. The average depth (Fig. 5d) decreases with time; the evident steps in the trend are related to the occurrence of new swarms. The *ζ* value moves from 2 to 1.2 km.

**Figure 6:**
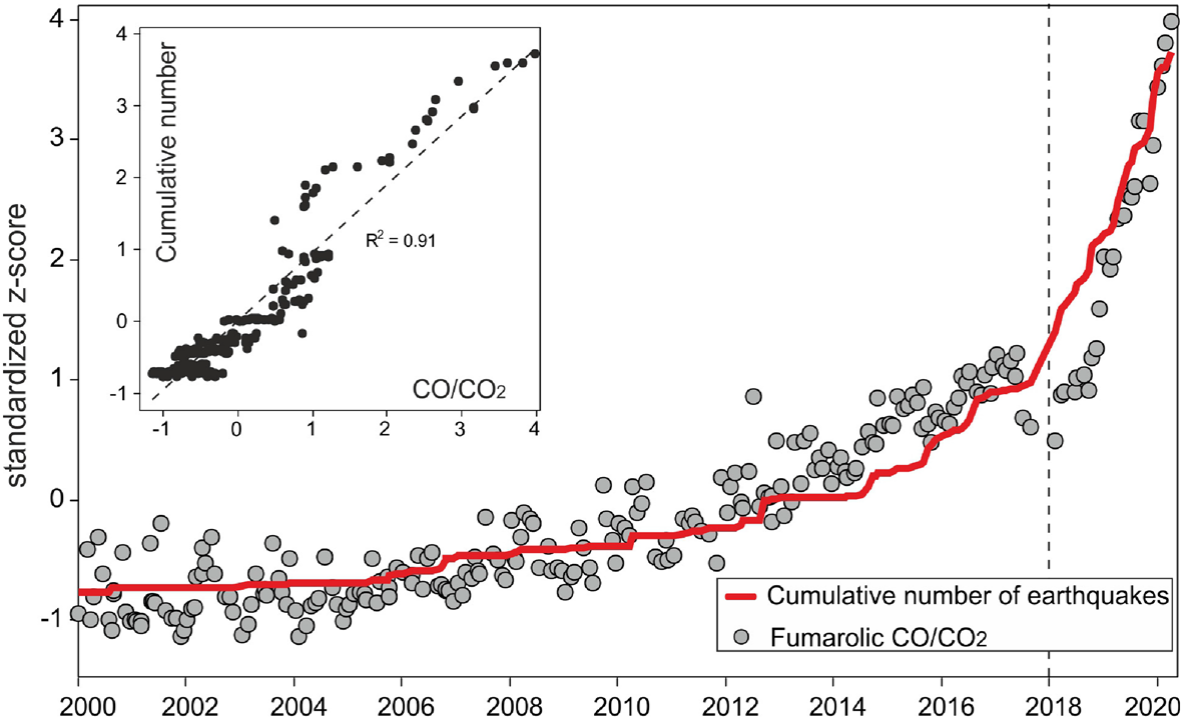
CO/CO_2_ fumarolic ratio and cumulative number of earthquakes normalized with the z-score method. The inside panel shows the same variables one versus the other.

All the dynamics are caused by the occurrence of earthquake swarms. A first step in the intertime is identifiable in 2015 coinciding with the swarm of the October 7, 2015. At the same time we notice a decrease in the average interdistance and in the average depth. This swarm occurred at the end of a period of uplift increment that stopped in coincidence with the swarm itself. The successive step occurred in July 2016 in coincidence with another swarm (54 detected earthquakes, *M*_*max*_ = 2.1)^29^. Again it occurred after an increment in the caldera uplift (Fig. 1) exhibiting the same trend of the curves (decrement of the interdistance and mean depth and an increment in the CO/CO_2_, Fig. 6), as observed for the previous swarm. For both the swarms the *b* value remains almost constant. At the end of 2017 two little swarms (*M*_*max*_ = 0.9) were recorded in the area of Solfatara/Pisciarelli and an increment in the *b* value is detectable. At the same time the interdistance and the mean depth are decreasing. The CO/CO_2_ showed a marked change in its trend (Fig. 6) and also the uplift started again to increase after a stable period (Fig. 1). From the end of 2018 to middle 2019 the *b* value showed a slightly decrement while mean depth and interdistance exhibit tiny variations in the trend. Another noticeable step occurs at the end of 2019 with the swarm of December 6, 2019. Here the earthquakes become shallower but, differently from the previous behavior, they slightly spread away. Anyway, except for the 2012 swarm, all the swarms are located close to the area of Solfatara/Pisciarelli.

**Figure 7:**
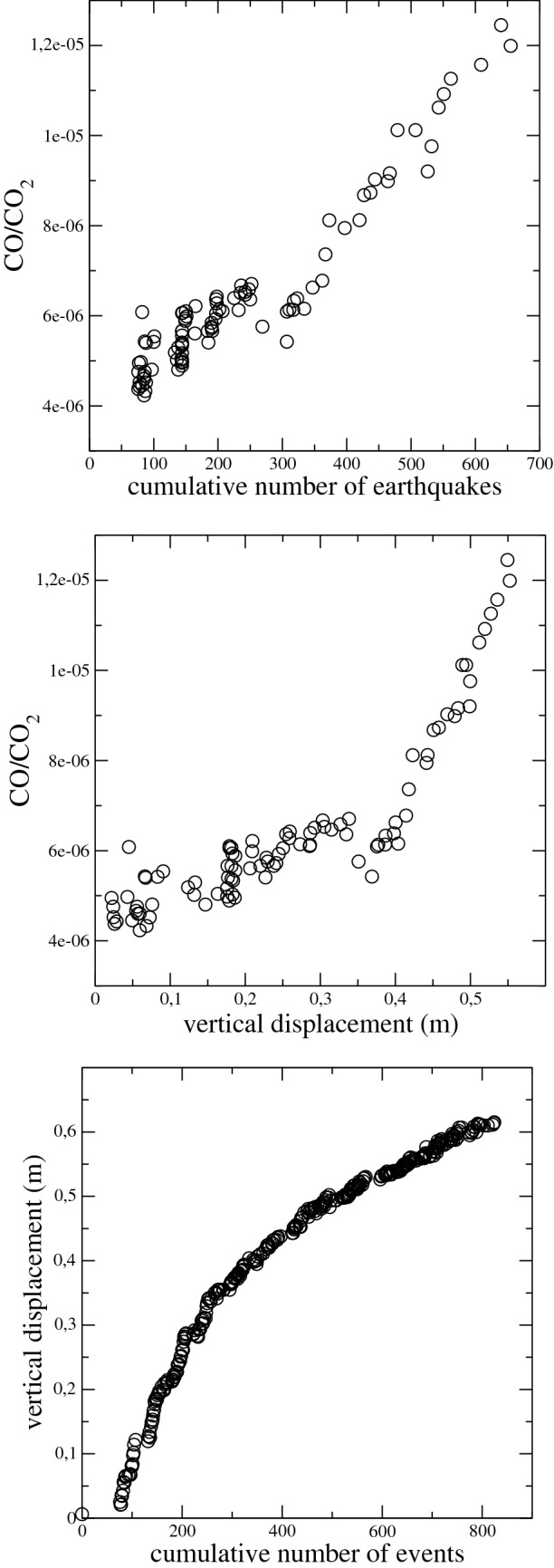
CO/CO_2_ fumarolic ratio versus the cumulative number of earthquakes, CO/CO_2_ fumarolic ratio versus the vertical displacement and the vertical displacement versus the cumulative number of earthquakes. In all cases the correlation between the variables appears to be very clear independently of the functional form of the dependence.

We would like to enlighten the common features of the analysed variables plotting each one versus the others. The plots reveal a clear dependence of each variable on the others. Figure 7 shows three examples of these dependence (the others are shown in the Supplementary Materials). In the great part of the cases, the correlation is self-evident and the functional dependence can be easily established (as an example the linear dependence of the CO/CO_2_ ratio on the cumulative number of events or the power law dependence of the soil deformation on the Δ*t*). In other cases the relationship cannot be so easily established (as for example for the dependence of the *b* value on the other variables). However, we would like to remark that the functional form of the dependence of each variable on the other is less relevant in this study than the existence of the dependence itself.

All the temporal variation of our observable can be possibly led back to a common cause, namely the fluid heating or pressurization clearly revealed by the CO/CO_2_ increase in time. This quantity is remarkably correlated with the cumulative number of events and the ground vertical deformation (see Figs. 6 and 7) enlightening a possible common cause for the temporal variations of the investigated variables.

Taking into account all the variations we suggest that the common cause can be identifiable with a fluid injection or pressurization. Injection of magmatic fluids within the hydrothermal system which feeds the Solfatara/Pisciarelli fumaroles would heat up the system increasing the pore pressure and facilitating the earthquakes occurrence being the medium less prone to bear stress and being the faults lubricated. Considering the vertical uplift the surficial manifestation of a deeper crustal bending, it could plays both as cause and consequence in this process being induced by the heating of the hydrothermal system and inducing fracture generation which recalls fluids towards the surface. A slightly decrement in the *V*_*s*_ velocity, compatible both with fluid presence and temperature increment, was evidenced by^30^ using the cross correlation of the seismic noise recorded in the caldera.

## Conclusions

The seismological and geochemical anomalies recorded in Campi Flegrei can be interpreted in terms of magmatic fluid circulation and permeability increment within the hydrothermal system. A magmatic intrusion or the injection of magmatic fluids were claimed as cause of the variations recorded from the beginning of the uplift until the 2012^8,23^, but starting from middle 2018 the behavior of the system showed a clear variation. The available tomographies [e.g.^31,32^] evidence that a seismic layer, characterized by low *V*_*p*_/*V*_*s*_ and low attenuation, was located at a depth of ~ 2.5 km, where most of the seismicity occurred. Its rheological properties could have suffered little variations due to vertical movement and heating. Geochemical analysis show that the gases involved in the hydrothermal system and sampled at the Solfatara/Pisciarelli fumarolic vents originate from a higher pressured system.

The rheological conditions of the central part of the caldera seems to be changing in time. Different rheological behaviors at Campi Flegrei have already been observed several times during the unrest and volcanic episodes occurred over the last 15 kyr^33^. Successions of brittle structures such as faults in contrast with levels of water saturated loose sands have been observed within the Campi Flegrei caldera. An increment in the temperature could recall hot fluids from the lower system increasing the pore pressures and lubricating the faults facilitating earthquake occurrence. The increment of the *b* value observed in the last decade as well as more dense and shallow swarms are compatible with this hypothesis. These observables temporal change appears to be correlated among themselves and with the ground deformations evidencing a variation in the hydrothermal system supported also by the increment in the CO/CO_2_ fumarolic ratio. In particular, the last quantity signs an increase of the temperature of the hydrothermal system since the end of 2017 that could be interpreted as due to fluid injection into the system or their pressurization. The CO/CO_2_ fumarolic ratio is remarkably correlated with the earthquakes cumulative number since 2000 (Fig. 6) indicating that a further increment could affect both the volcanological and seismological hazard.

## Supplementary information

Supplementary Information 1.

Supplementary Information 2.

Supplementary Information 3.

## Data Availability

The seismic catalog is available at the website of Osservatorio Vesuviano: http://sismolab.ov.ingv.it/sismo/. CO/CO_2_ data since 2017 are reported in the Supplementary. Previous data are reported in^7,34^. The vertical displacements recored at station RITE are reported in the Supplementary.
