# Supplementary material for: Statistics of seismicity to investigate the Campi Flegrei caldera unrest
Source: Sci Rep. 2021 Mar 30;11:7211. doi: 10.1038/s41598-021-86506-6 (PMC8009907; doi:10.1038/s41598-021-86506-6)
Supplement: Supplementary file 1 — Supplementary Information 1. [file 41598_2021_86506_MOESM1_ESM.pdf]

Supplementary materials
Statistics of seismicity to investigate the Campi Flegrei caldera unrest

A. Tramelli[a], C. Godano [a,b], P. Ricciolino[a], F. Giudicepietro[a], S. Caliro [a], M. Orazi [a], P. De Martino [a] and G. Chiodini[a]

[a]Istituto Nazionale di Geofisica e Vulcanologia, Osservatorio Vesuviano, Napoli, Italy
[b]Department of Mathematics and Physics, Second University of Naples, Caserta, Italy

January 21, 2021

1 Events window analysis

The choice of a window size of 150 earthquakes is based on the analysis performed on the Campi Flegrei seismic catalogue with different window sizes and on a simple simulation. Firstly, we generate a catalogue of 500000 earthquakes with magnitudes following the Gutenberg and Richter law with a $b = 1$ and in the range $1.5 \leq M \leq 7.0$. Then we randomly select 100000 samples of n events where n spans from 50 to 500 with a step of 10. For each sample we evaluate the corresponding b value and finally evaluate the average of the 100000 obtained values $\langle b \rangle$ and their standard deviation σ for each value of n. Figure 1 shows $\langle b \rangle$ as a function of n. Error bars represents σ.

In order to have a quantitative criterion of selection of the correct n we evaluate the difference, $\Delta\sigma$ between two successive σs (Figure 2)

The function $\Delta\sigma$ versus n is well represented by a power law $\Delta\sigma \propto n^{-\alpha}$ and we decided that the best choice for n could be when $\Delta\sigma$ reduces of a factor $10^{1-\alpha}$. This approximately occurs at $n = 150$.

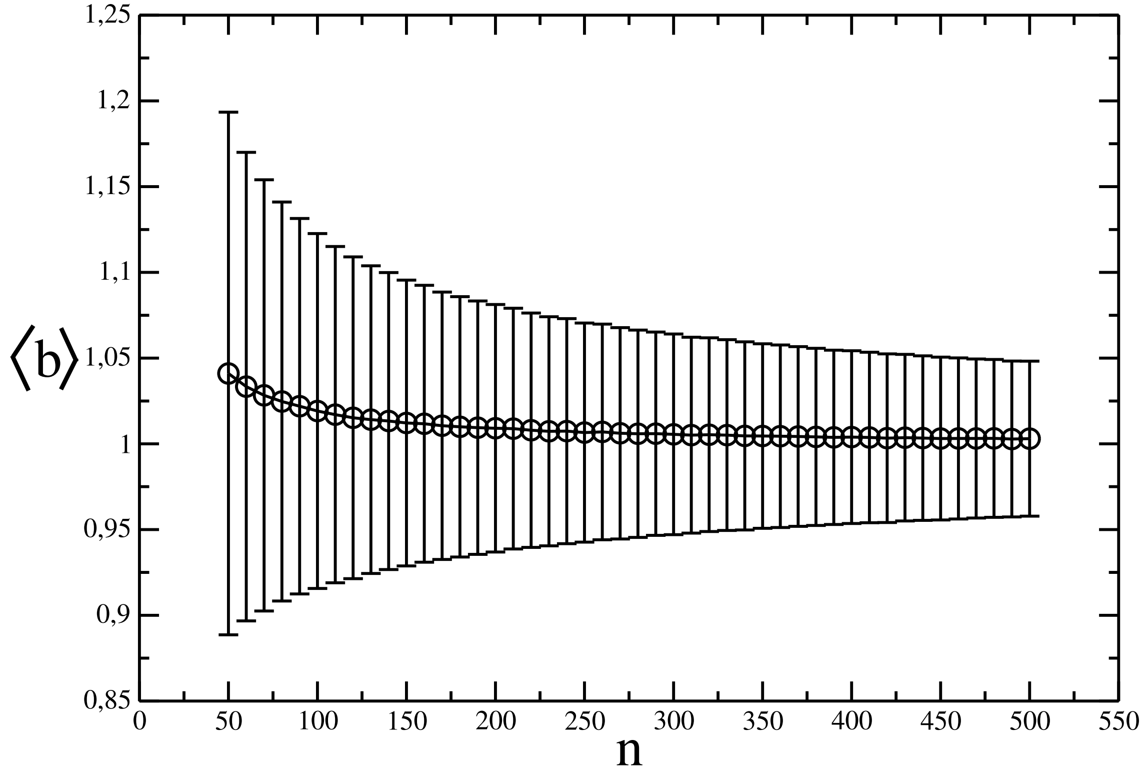

Figure 1: $\langle b \rangle$ as a function of n. Error bars represents σ.

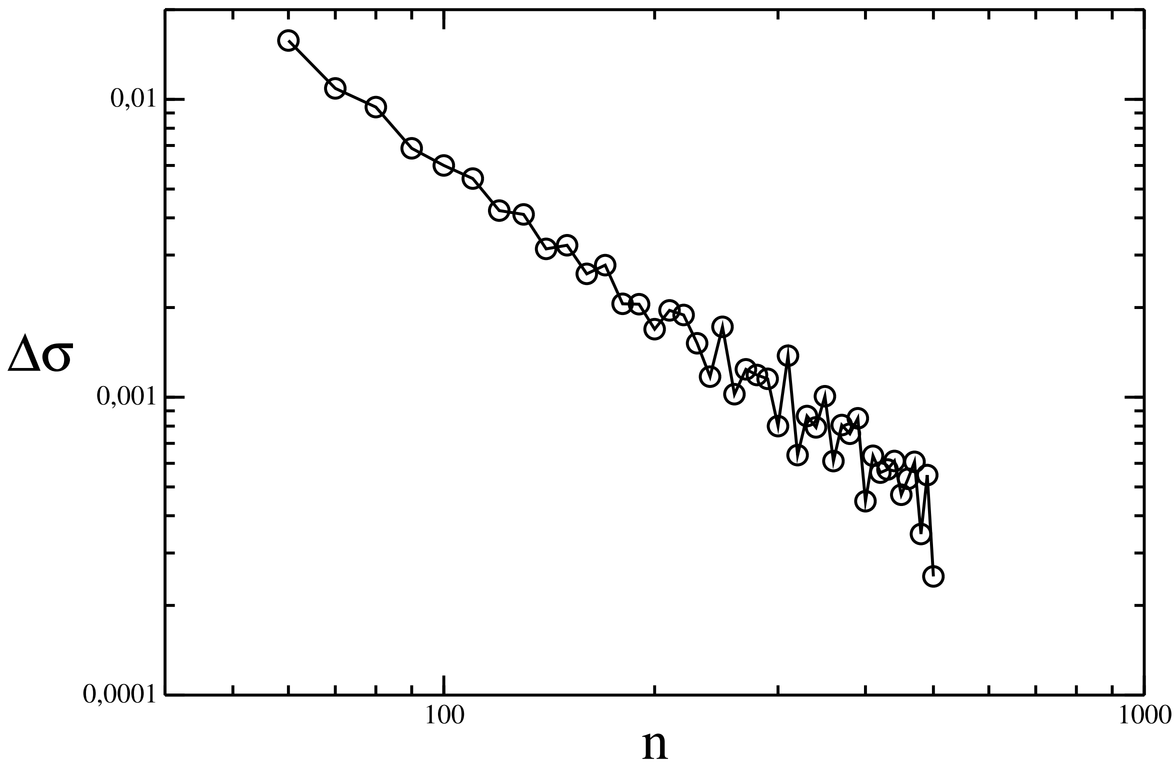

Figure 2: $\Delta\sigma$ as a function of n.

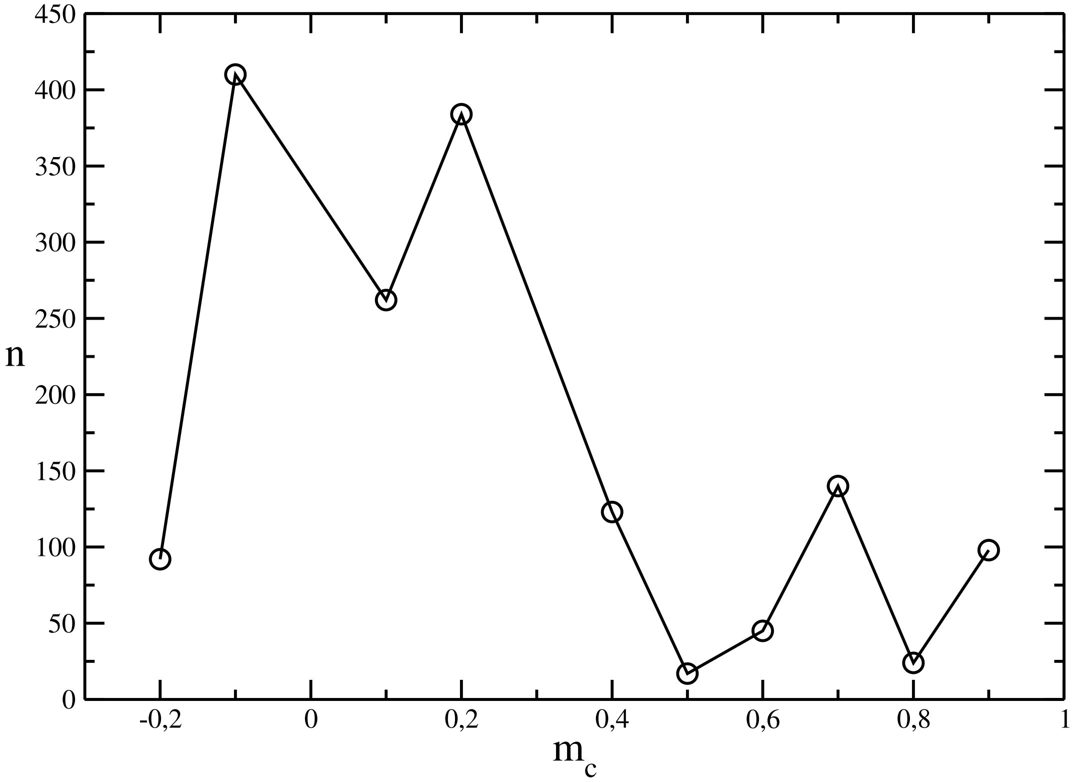

Figure 3: The M_c distribution.

2 The choice of M_c

The distribution of the M_c values (figure 3) shows that the great part of the obtained (using the method described in the main text) values are $M_c = -0.1$ and $M_c = 0.2$. However, some M_c values are larger than 0.4 with a maximum M_c equal to 0.9. In the following we will show as the larger M_c values could be due to artefacts.

Figure 4 shows four Gutenberg-Richter distributions which are substantially overlapped, except for 3 events with magnitudes 0.8 and 0.9, but the completeness magnitude assumes different values. Namely -0.1 for two of them and 0.8 for the other two. Indeed the occurrence of 2 more earthquakes (with magnitude 0.8 and 0.9) in one case (green squares in figure) and of 3 more earthquakes (two with magnitude 0.8 and one 0.9) in the other slightly changes the residuals passing from 0.298 (at $M_c = -0.1$) to 0.294 (at $M_c = 0.8$) in the first case and from 0.305 (at $M_c = -0.1$) to 0.286 (at

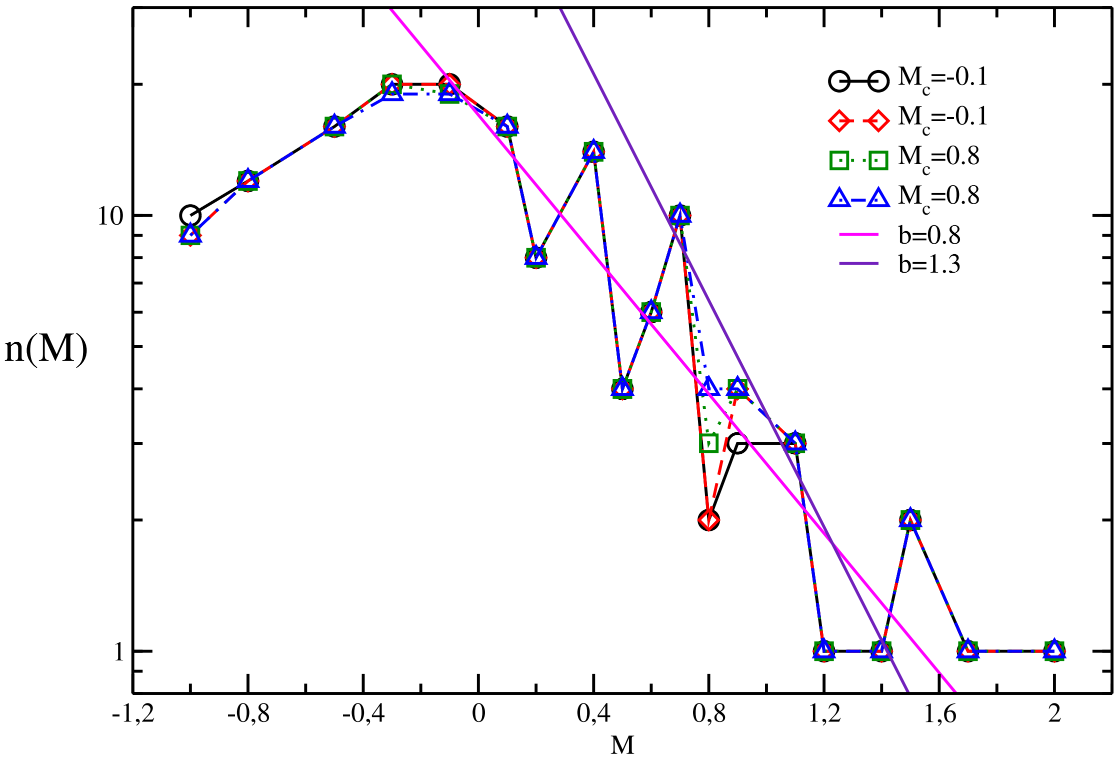

Figure 4: The Gutenberg-Richter distribution for four different time windows. The magenta line represents a fit with $b = 0.8$ plotted as a guide for the eyes. The blue line represent the fit with $b = 1.3$.

$M_c = 0.8$) in the second one. The assumption of different Mcs would introduce dummy jumps because the magnitudes window used for the estimate is too short. To prevent this situation we visually checked di Gutemberg Riechter fits to data. The fit with a b value fixed at 0.8, plotted as a guide for the eyes, clearly reveals that a $M_c = -0.1$ appears to be more appropriate for all the Gutenberg-Richter distributions revealing that the choice of $M_c = 0.8$ is an artefact due to the completeness magnitude estimation method. In this case the too short interval $[M_c - M_max]$ affects the stability of the fit leading to a b of 1.3 (figure 4). Very similar artefacts are observed even using different methods as the maximum curvature or the entire-range ones. As a consequence we decided to adopt a constant $M_c = 0.2$ for all the time windows. Indeed increasing M_c form -0.1 to 0.2 does not change significantly the estimated b value.

3 Magnitude error

The error of the magnitude has great influence on the b value estimation. In order to evaluate its order of magnitude we start from the formula that is used for the M_d estimation in Campi Flegrei:

$$M_d = -2.46(\pm 0.03) + 2.82(\pm 0.041)\log T \qquad (1)$$

Th error propagation rules lead to the M_d error

$$\epsilon_{M_d} = \sqrt{(2.46 \cdot 0.03)^2 + [(M + 2.46) \cdot 0.041]^2} \qquad (2)$$

where we have neglected the error on T because one order of magnitude smaller than the others. The Gutenberg-Richter distribution showing ϵ_{M_d} is reported in figure 5. As can be seen the ϵ_{M_d} influences the estimation of the b value generating an error 0.04 which is equivalent to the one obtained with the Shi and Bolt formula. As a consequence we decided to adopt, for simplicity, the last one error.

4 The correlation between the variables

In the following we show the plot of each variable estimated or used in the article versus each other. Three of these plots are shown in the main text as examples. The panels evidence all the possible correlation between variables.

Figures 6, 7 and 8 show that most of the variables are correlated to each other. In some cases (in particular when the b value is involved) the correlation is worst, however, even in these cases, a trend can be individuated. Notice the presence of a straight line for low values of ξ. This can be viewed as a sort of finite size effect. Namely, using 150 events, is not possible to cover an area smaller than 1 km^2. In some cases the correlation is linear, in others exponential and or a power law. We would like to remark that the kind of correlation is, here, not important. What is relevant, is that they are all correlated to each other evidencing the possible existence of a common cause for their temporal behaviour.

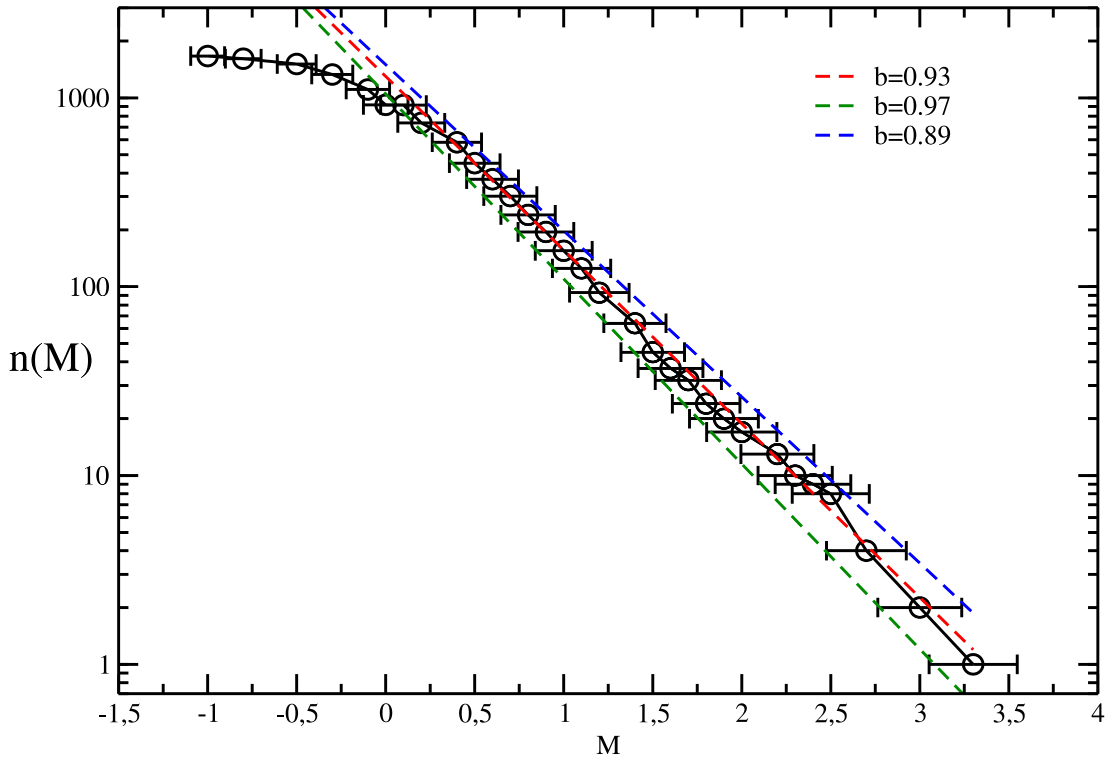

Figure 5: The Gutenberg-Richter distribution showing ϵ_{M_d}. The three dashed lines represents the fitted Gutenberg-Richter laws defining the confidence interval for the b value.

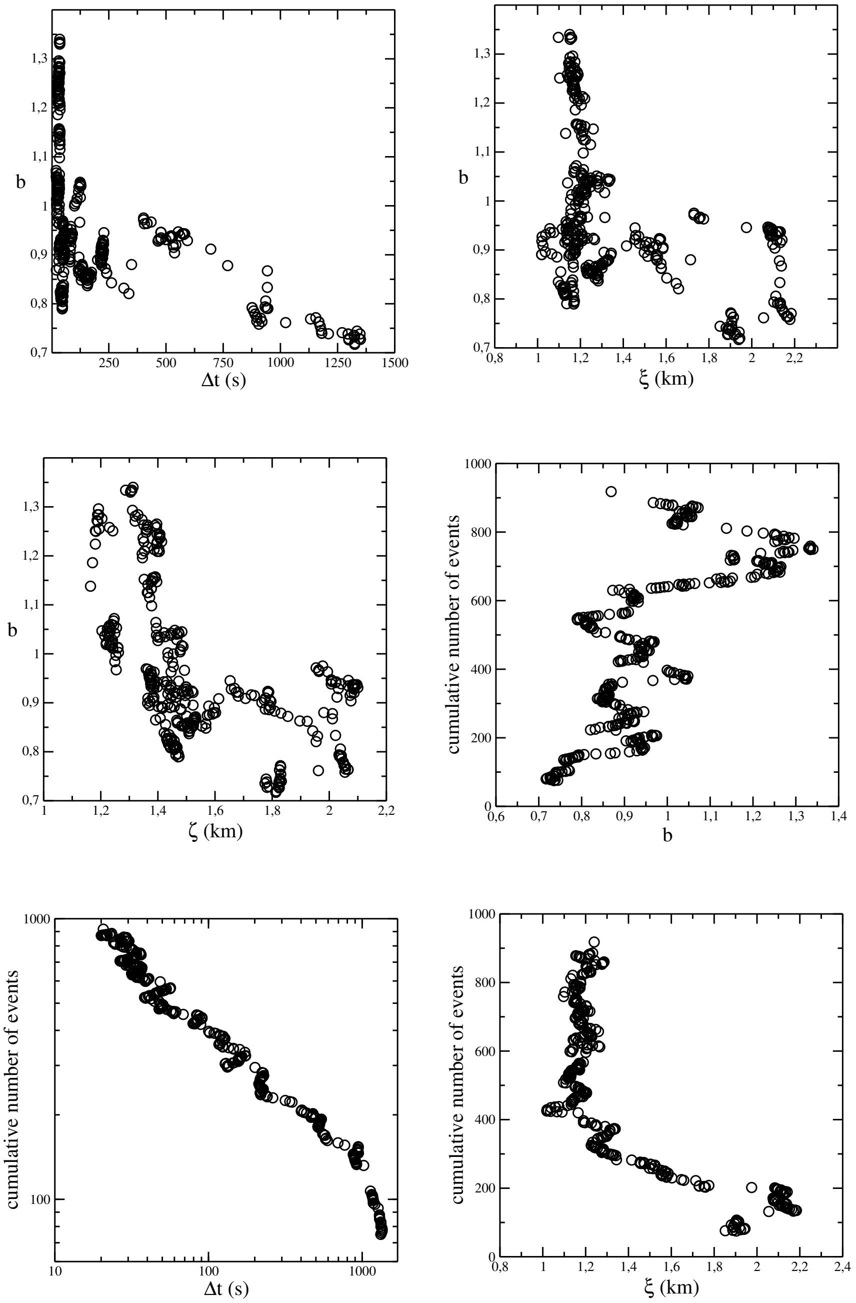

Figure 6: Correlations between couples of variables analysed in the article.

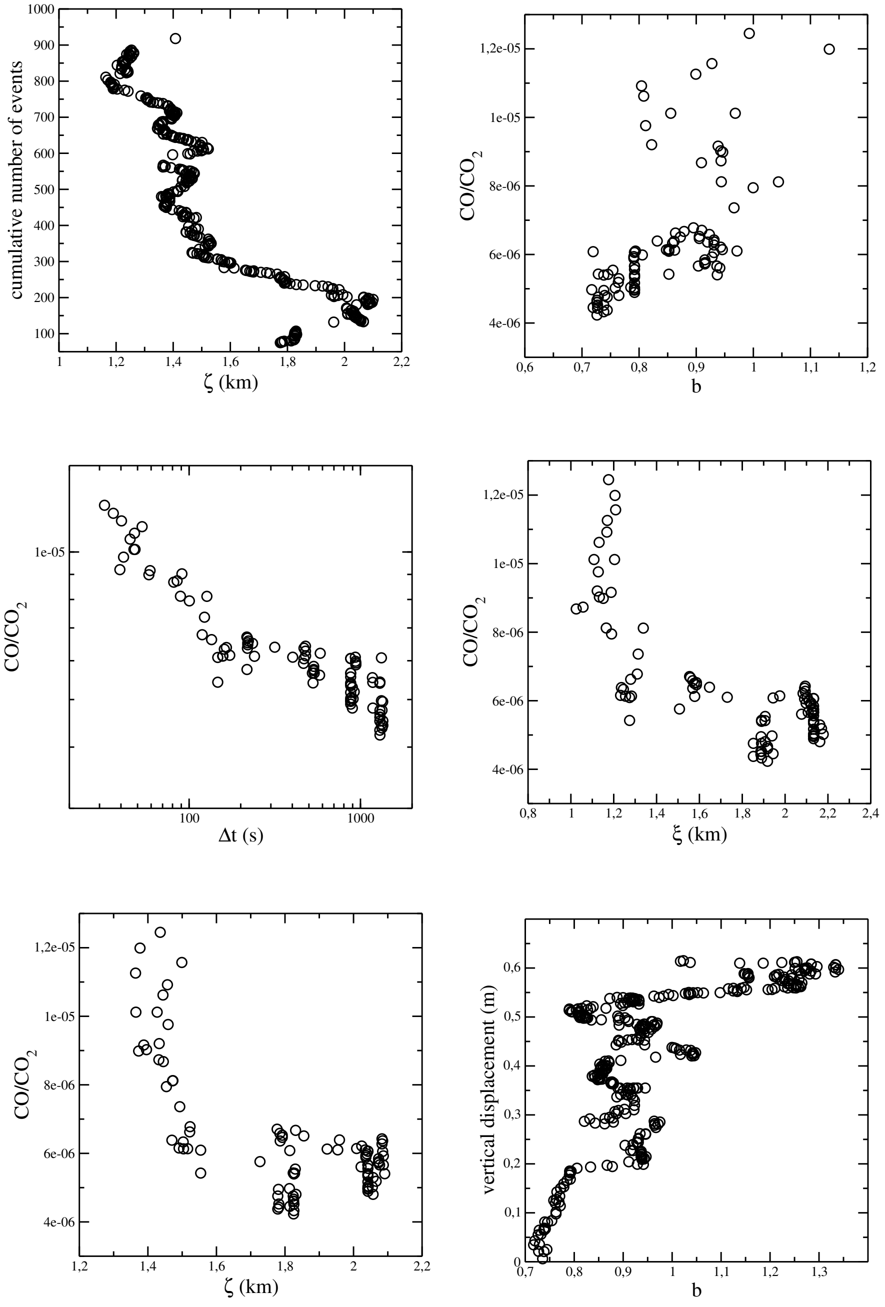

Figure 7: Correlations between couples of variables analysed in the article.

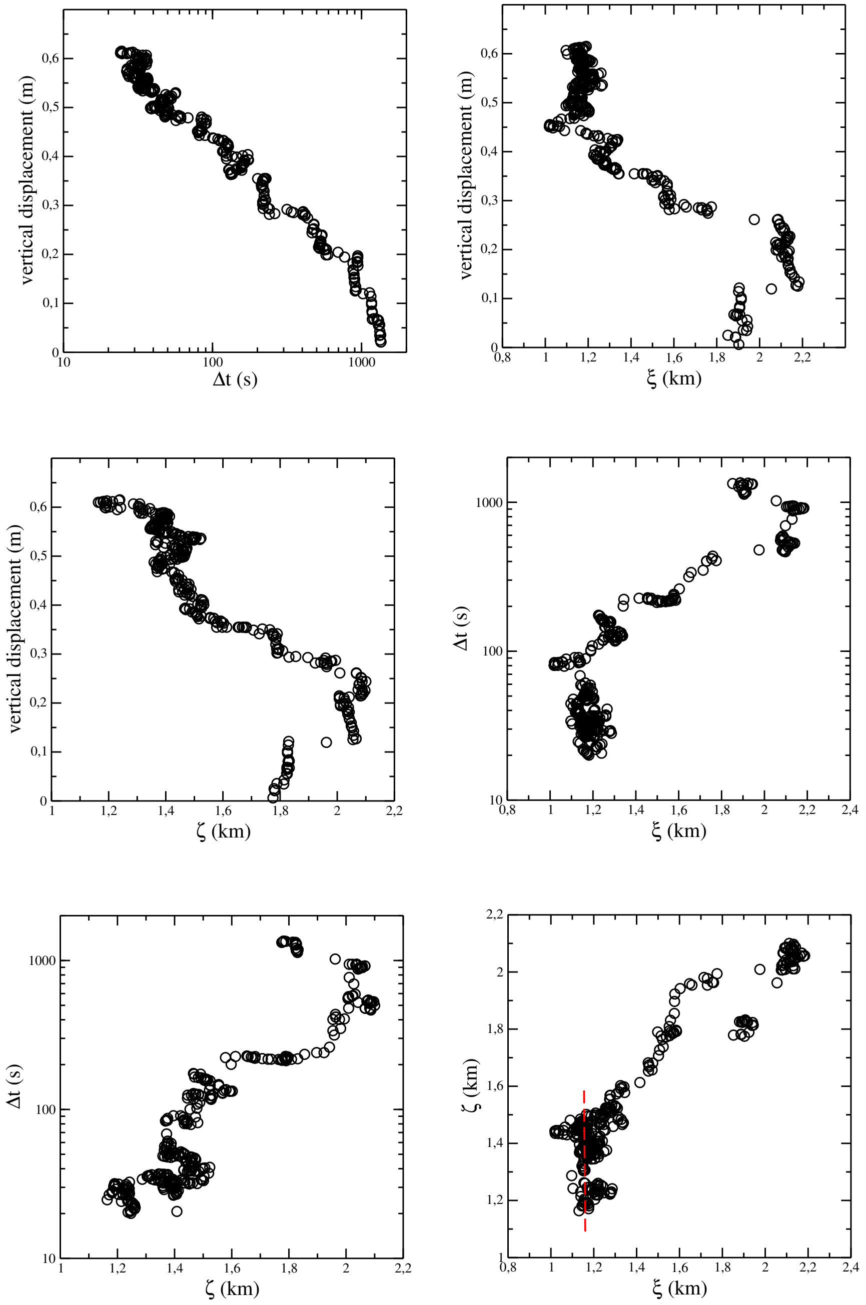

Figure 8: Correlations between couples of variables analysed in the article.
