# Supplementary material for: Statistics of seismicity to investigate the Campi Flegrei caldera unrest
Source: Sci Rep. 2021 Mar 30;11:7211. doi: 10.1038/s41598-021-86506-6 (PMC8009907; doi:10.1038/s41598-021-86506-6)
Supplement: Supplementary file 2 — Supplementary Information 2. [file 41598_2021_86506_MOESM2_ESM.pdf]

Day	CO/CO2
1/11/2017	6.67179E-06
2/20/2017	6.52757E-06
3/16/2017	6.46611E-06
4/18/2017	6.58392E-06
5/12/2017	6.36082E-06
5/30/2017	6.70613E-06
7/7/2017	5.75772E-06
8/29/2017	5.61915E-06
2/12/2018	5.42369E-06
3/19/2018	6.09326E-06
4/18/2018	6.1348E-06
6/25/2018	6.13188E-06
7/9/2018	6.33341E-06
8/30/2018	6.38429E-06
9/26/2018	6.15537E-06
10/16/2018	6.6262E-06
11/19/2018	6.77948E-06
12/4/2018	7.36079E-06
1/7/2019	8.11801E-06
2/19/2019	7.94738E-06
3/12/2019	8.12248E-06
4/8/2019	8.67711E-06
5/21/2019	8.73242E-06
6/18/2019	9.02519E-06
7/15/2019	8.98479E-06
8/5/2019	9.16121E-06
9/4/2019	1.01194E-05
10/8/2019	1.01156E-05
11/11/2019	9.20323E-06
12/2/2019	9.76006E-06
1/9/2020	1.06228E-05
2/3/2020	1.09181E-05
3/2/2020	1.12595E-05
4/6/2020	1.15736E-05
