# Supplementary material for: Statistics of seismicity to investigate the Campi Flegrei caldera unrest
Source: Sci Rep. 2021 Mar 30;11:7211. doi: 10.1038/s41598-021-86506-6 (PMC8009907; doi:10.1038/s41598-021-86506-6)
Supplement: Supplementary file 3 — Supplementary Information 3. [file 41598_2021_86506_MOESM3_ESM.pdf]

Date (dd/mm/yyyy)	Vertical displacement (m)
5/29/2000	0.0000
5/30/2000	0.0171
5/31/2000	0.0093
6/1/2000	0.0135
6/2/2000	0.0182
6/3/2000	0.0118
6/4/2000	0.0093
6/5/2000	0.0138
6/6/2000	0.0143
6/7/2000	0.0126
6/9/2000	0.0094
6/10/2000	0.0030
6/11/2000	0.0047
6/12/2000	0.0114
6/13/2000	0.0141
6/14/2000	0.0161
6/16/2000	0.0185
6/17/2000	0.0227
6/18/2000	0.0103
6/19/2000	0.0209
6/20/2000	0.0132
6/21/2000	0.0105
6/23/2000	0.0105
6/24/2000	0.0206
6/25/2000	0.0137
6/26/2000	0.0117
6/27/2000	0.0142
6/28/2000	0.0176
6/30/2000	0.0182
7/1/2000	0.0320
7/2/2000	0.0253
7/3/2000	0.0310
7/4/2000	0.0267
7/5/2000	0.0296
7/6/2000	0.0264
7/7/2000	0.0270
7/8/2000	0.0262
7/9/2000	0.0289
7/10/2000	0.0260
7/11/2000	0.0116
7/12/2000	0.0252
7/13/2000	0.0304
7/14/2000	0.0374
7/15/2000	0.0289
7/16/2000	0.0288
7/17/2000	0.0340
7/18/2000	0.0314
7/22/2000	0.0331
7/23/2000	0.0342

7/24/2000	0.0311
7/25/2000	0.0324
7/27/2000	0.0383
7/28/2000	0.0393
7/29/2000	0.0339
7/30/2000	0.0406
7/31/2000	0.0396
8/2/2000	0.0386
8/3/2000	0.0413
8/4/2000	0.0445
8/5/2000	0.0332
8/6/2000	0.0419
8/7/2000	0.0426
8/8/2000	0.0315
8/9/2000	0.0493
8/10/2000	0.0444
8/11/2000	0.0352
8/12/2000	0.0416
8/13/2000	0.0367
8/14/2000	0.0435
8/15/2000	0.0377
8/16/2000	0.0310
8/18/2000	0.0405
8/19/2000	0.0417
8/20/2000	0.0412
8/21/2000	0.0444
8/22/2000	0.0384
8/25/2000	0.0404
8/26/2000	0.0418
8/27/2000	0.0311
8/28/2000	0.0209
8/29/2000	0.0384
8/31/2000	0.0433
9/1/2000	0.0560
9/2/2000	0.0295
9/3/2000	0.0468
9/4/2000	0.0502
9/5/2000	0.0373
9/6/2000	0.0369
9/7/2000	0.0160
9/8/2000	0.0359
9/9/2000	0.0311
9/11/2000	0.0459
9/12/2000	0.0422
9/13/2000	0.0464
9/14/2000	0.0357
9/15/2000	0.0400
9/16/2000	0.0352
9/17/2000	0.0401
9/18/2000	0.0291

9/19/2000	0.0380
9/20/2000	0.0314
9/21/2000	0.0277
9/23/2000	0.0376
9/24/2000	0.0267
9/25/2000	0.0440
9/26/2000	0.0395
9/27/2000	0.0343
9/28/2000	0.0385
9/29/2000	0.0396
9/30/2000	0.0305
10/1/2000	0.0291
10/2/2000	0.0395
10/3/2000	0.0324
10/4/2000	0.0665
10/7/2000	0.0317
10/8/2000	0.0355
10/9/2000	0.0322
10/10/2000	0.0336
10/11/2000	0.0263
10/12/2000	0.0331
10/13/2000	0.0316
10/14/2000	0.0390
10/15/2000	0.0363
10/16/2000	0.0293
10/17/2000	0.0437
10/18/2000	0.0303
10/19/2000	0.0361
10/20/2000	0.0370
10/21/2000	0.0383
10/22/2000	0.0350
10/23/2000	0.0353
10/24/2000	0.0355
10/25/2000	0.0357
11/19/2000	0.0219
11/20/2000	0.0314
11/22/2000	0.0265
11/23/2000	0.0303
11/24/2000	0.0317
11/25/2000	0.0241
11/26/2000	0.0329
11/27/2000	0.0309
11/28/2000	0.0271
11/29/2000	0.0244
12/1/2000	0.0250
12/2/2000	0.0278
12/3/2000	0.0273
12/4/2000	0.0294
12/6/2000	0.0255
12/9/2000	0.0296

12/10/2000	0.0265
12/11/2000	0.0265
12/12/2000	0.0316
12/13/2000	0.0306
12/14/2000	0.0265
12/15/2000	0.0331
12/17/2000	0.0287
12/18/2000	0.0250
12/19/2000	0.0216
12/20/2000	0.0245
12/22/2000	0.0226
12/23/2000	0.0250
12/24/2000	0.0205
12/25/2000	0.0201
12/26/2000	0.0252
12/27/2000	0.0149
12/28/2000	0.0183
12/29/2000	0.0203
12/30/2000	0.0211
12/31/2000	0.0275
1/1/2001	0.0215
1/2/2001	0.0181
1/3/2001	0.0115
1/4/2001	0.0225
1/5/2001	0.0182
1/6/2001	0.0283
1/7/2001	0.0235
1/8/2001	0.0156
1/9/2001	0.0160
1/10/2001	0.0184
1/11/2001	0.0226
1/12/2001	0.0257
1/13/2001	0.0281
1/14/2001	0.0289
1/15/2001	0.0259
1/16/2001	0.0245
1/17/2001	0.0258
1/18/2001	0.0268
1/19/2001	0.0186
1/20/2001	0.0266
1/21/2001	0.0209
1/22/2001	0.0192
1/23/2001	0.0210
1/24/2001	0.0221
1/25/2001	0.0252
1/26/2001	0.0225
1/27/2001	0.0196
1/28/2001	0.0259
1/29/2001	0.0246
1/30/2001	0.0254

1/31/2001	0.0222
2/1/2001	0.0329
2/2/2001	0.0249
2/3/2001	0.0202
2/4/2001	0.0192
2/5/2001	0.0133
2/6/2001	0.0209
2/7/2001	0.0164
2/8/2001	0.0217
2/9/2001	0.0182
2/10/2001	0.0222
2/11/2001	0.0177
2/12/2001	0.0219
2/13/2001	0.0208
2/14/2001	0.0239
2/15/2001	0.0210
2/17/2001	0.0199
2/18/2001	0.0197
2/19/2001	0.0196
2/20/2001	0.0235
2/21/2001	0.0185
2/22/2001	0.0175
2/23/2001	0.0171
2/24/2001	0.0191
2/25/2001	0.0185
2/26/2001	0.0140
2/27/2001	0.0133
2/28/2001	0.0145
3/1/2001	0.0068
3/2/2001	0.0083
3/3/2001	0.0042
3/4/2001	0.0057
3/5/2001	0.0065
3/6/2001	0.0136
3/7/2001	0.0112
3/8/2001	0.0101
3/10/2001	0.0075
3/11/2001	0.0099
3/12/2001	0.0157
3/13/2001	0.0123
3/14/2001	0.0134
3/15/2001	0.0116
3/16/2001	0.0088
3/17/2001	0.0123
3/18/2001	0.0071
3/19/2001	0.0111
3/20/2001	0.0092
3/21/2001	0.0116
3/22/2001	0.0134
3/23/2001	0.0155

3/24/2001	0.0054
3/25/2001	0.0090
3/27/2001	0.0090
3/28/2001	0.0062
3/29/2001	0.0137
3/30/2001	0.0140
3/31/2001	0.0019
4/1/2001	0.0172
4/2/2001	0.0119
4/3/2001	0.0102
4/5/2001	0.0059
4/12/2001	0.0112
4/13/2001	0.0067
4/14/2001	0.0043
4/16/2001	0.0013
4/18/2001	0.0071
4/19/2001	0.0056
4/20/2001	0.0054
4/21/2001	0.0013
4/22/2001	0.0023
4/23/2001	0.0055
4/24/2001	-0.0045
4/25/2001	-0.0016
4/26/2001	0.0024
4/27/2001	0.0031
4/28/2001	0.0017
4/29/2001	0.0000
4/30/2001	0.0042
5/1/2001	0.0148
5/2/2001	0.0033
5/3/2001	0.0046
5/4/2001	0.0029
5/5/2001	0.0045
5/6/2001	0.0015
5/7/2001	-0.0002
5/8/2001	0.0030
5/9/2001	0.0016
5/10/2001	0.0062
5/11/2001	0.0023
5/12/2001	-0.0030
5/13/2001	0.0034
5/14/2001	0.0007
5/15/2001	-0.0068
5/16/2001	-0.0007
5/17/2001	-0.0005
5/18/2001	-0.0001
5/19/2001	0.0038
5/20/2001	-0.0062
5/21/2001	0.0001
5/22/2001	-0.0045

5/23/2001	-0.0016
5/24/2001	0.0070
5/29/2001	0.0074
5/30/2001	-0.0045
5/31/2001	0.0025
6/1/2001	0.0039
6/2/2001	-0.0013
6/3/2001	0.0039
6/4/2001	0.0023
6/5/2001	-0.0022
6/6/2001	-0.0025
6/7/2001	-0.0030
6/8/2001	-0.0051
6/9/2001	-0.0015
6/10/2001	0.0017
6/11/2001	0.0065
6/12/2001	0.0007
6/13/2001	0.0048
6/14/2001	0.0034
6/15/2001	-0.0008
6/16/2001	-0.0034
6/17/2001	-0.0015
6/18/2001	0.0018
6/19/2001	0.0017
6/20/2001	-0.0028
6/21/2001	-0.0067
6/22/2001	-0.0003
6/23/2001	0.0008
6/24/2001	-0.0015
6/25/2001	-0.0044
6/26/2001	-0.0109
6/27/2001	0.0023
6/28/2001	0.0042
6/29/2001	-0.0110
6/30/2001	-0.0024
7/1/2001	-0.0010
7/2/2001	0.0015
7/3/2001	-0.0020
7/4/2001	0.0012
7/5/2001	-0.0014
7/6/2001	-0.0013
7/7/2001	0.0019
7/8/2001	-0.0003
7/9/2001	-0.0052
7/10/2001	-0.0024
7/11/2001	0.0003
7/12/2001	-0.0031
7/13/2001	-0.0049
7/14/2001	-0.0107
7/15/2001	0.0059

7/16/2001	-0.0041
7/18/2001	-0.0062
7/19/2001	-0.0026
7/20/2001	-0.0029
7/21/2001	-0.0040
7/22/2001	0.0038
7/23/2001	-0.0003
7/24/2001	-0.0014
7/25/2001	-0.0096
7/26/2001	0.0017
7/27/2001	-0.0008
7/28/2001	-0.0022
7/29/2001	0.0005
7/30/2001	-0.0064
7/31/2001	0.0003
8/1/2001	0.0011
8/2/2001	-0.0048
8/3/2001	0.0001
8/4/2001	-0.0021
8/5/2001	-0.0013
8/7/2001	-0.0043
8/9/2001	-0.0079
8/10/2001	0.0028
8/11/2001	-0.0064
8/12/2001	-0.0034
8/13/2001	-0.0051
8/14/2001	-0.0039
8/15/2001	-0.0114
8/16/2001	-0.0006
8/17/2001	-0.0055
8/18/2001	-0.0061
8/19/2001	-0.0102
8/20/2001	-0.0026
8/21/2001	0.0035
8/22/2001	-0.0030
8/23/2001	-0.0102
8/24/2001	-0.0044
8/25/2001	-0.0021
8/26/2001	-0.0058
8/27/2001	0.0004
8/28/2001	0.0012
8/29/2001	-0.0105
8/30/2001	-0.0039
8/31/2001	0.0046
9/1/2001	-0.0014
9/2/2001	-0.0057
9/3/2001	-0.0107
9/6/2001	-0.0054
9/7/2001	-0.0010
9/8/2001	-0.0098

9/9/2001	-0.0111
9/10/2001	-0.0126
9/11/2001	-0.0051
9/12/2001	-0.0116
9/13/2001	-0.0046
9/14/2001	-0.0108
9/15/2001	0.0010
9/16/2001	-0.0069
9/17/2001	-0.0078
9/18/2001	-0.0077
9/19/2001	-0.0124
9/20/2001	-0.0109
9/21/2001	-0.0151
9/22/2001	-0.0132
9/23/2001	-0.0097
9/24/2001	-0.0073
9/25/2001	-0.0156
9/26/2001	-0.0228
9/27/2001	-0.0162
9/28/2001	-0.0131
9/29/2001	-0.0158
9/30/2001	-0.0173
10/1/2001	-0.0135
10/2/2001	-0.0173
10/3/2001	-0.0084
10/4/2001	-0.0104
10/5/2001	-0.0120
10/6/2001	-0.0183
10/7/2001	-0.0086
10/8/2001	-0.0132
10/9/2001	-0.0138
10/10/2001	-0.0107
10/11/2001	-0.0139
10/12/2001	-0.0064
10/13/2001	-0.0126
10/14/2001	-0.0129
10/15/2001	-0.0054
10/16/2001	-0.0140
10/17/2001	-0.0174
10/18/2001	-0.0156
10/19/2001	-0.0165
10/20/2001	-0.0151
10/21/2001	-0.0091
10/22/2001	-0.0194
10/23/2001	-0.0190
10/24/2001	-0.0104
10/25/2001	-0.0134
10/26/2001	-0.0137
10/27/2001	-0.0184
10/28/2001	-0.0107

10/29/2001	-0.0197
10/30/2001	-0.0127
10/31/2001	-0.0151
11/1/2001	-0.0101
11/7/2001	-0.0157
11/8/2001	-0.0164
11/10/2001	-0.0131
11/11/2001	-0.0246
11/12/2001	-0.0124
11/13/2001	-0.0167
11/14/2001	-0.0159
11/15/2001	-0.0162
11/16/2001	-0.0130
11/17/2001	-0.0168
11/18/2001	-0.0190
11/19/2001	-0.0162
11/20/2001	-0.0100
11/21/2001	-0.0161
11/22/2001	-0.0187
11/23/2001	-0.0075
11/24/2001	-0.0081
11/25/2001	-0.0147
11/26/2001	-0.0141
11/27/2001	-0.0179
11/28/2001	-0.0126
11/29/2001	-0.0091
11/30/2001	-0.0155
12/1/2001	-0.0099
12/2/2001	-0.0117
12/3/2001	-0.0140
12/4/2001	-0.0173
12/5/2001	-0.0144
12/6/2001	-0.0113
12/7/2001	-0.0113
12/8/2001	-0.0133
12/9/2001	-0.0159
12/10/2001	-0.0150
12/11/2001	-0.0133
12/12/2001	-0.0136
12/14/2001	-0.0163
12/15/2001	-0.0179
12/16/2001	-0.0175
12/17/2001	-0.0136
12/19/2001	-0.0153
12/22/2001	-0.0146
12/23/2001	-0.0173
12/24/2001	-0.0139
12/25/2001	-0.0156
12/26/2001	-0.0118
1/5/2002	-0.0133

1/6/2002	-0.0176
1/7/2002	-0.0192
1/8/2002	-0.0222
1/9/2002	-0.0218
1/10/2002	-0.0165
1/11/2002	-0.0190
1/12/2002	-0.0197
1/13/2002	-0.0180
1/14/2002	-0.0145
1/15/2002	-0.0189
1/16/2002	-0.0177
1/17/2002	-0.0154
1/18/2002	-0.0183
1/20/2002	-0.0196
1/23/2002	-0.0220
1/24/2002	-0.0162
1/25/2002	-0.0176
1/26/2002	-0.0263
1/27/2002	-0.0202
1/29/2002	-0.0203
1/30/2002	-0.0255
1/31/2002	-0.0233
2/5/2002	-0.0167
2/6/2002	-0.0252
2/8/2002	-0.0154
2/9/2002	-0.0200
2/10/2002	-0.0219
2/11/2002	-0.0203
2/12/2002	-0.0185
2/13/2002	-0.0231
2/14/2002	-0.0258
2/15/2002	-0.0069
2/16/2002	-0.0230
2/17/2002	-0.0207
2/18/2002	-0.0233
2/19/2002	-0.0277
2/20/2002	-0.0302
2/21/2002	-0.0213
2/23/2002	-0.0286
2/24/2002	-0.0261
2/26/2002	-0.0295
2/27/2002	-0.0301
2/28/2002	-0.0239
3/1/2002	-0.0222
3/2/2002	-0.0258
3/3/2002	-0.0186
3/4/2002	-0.0291
3/5/2002	-0.0274
3/6/2002	-0.0306
3/7/2002	-0.0265

3/8/2002	-0.0268
3/9/2002	-0.0307
3/10/2002	-0.0243
3/11/2002	-0.0307
3/12/2002	-0.0217
3/13/2002	-0.0280
3/14/2002	-0.0287
3/15/2002	-0.0320
3/16/2002	-0.0310
3/17/2002	-0.0290
3/18/2002	-0.0281
3/19/2002	-0.0353
3/20/2002	-0.0287
3/21/2002	-0.0313
3/22/2002	-0.0282
3/23/2002	-0.0242
3/24/2002	-0.0310
3/25/2002	-0.0317
3/26/2002	-0.0236
3/29/2002	-0.0297
3/30/2002	-0.0304
3/31/2002	-0.0308
4/1/2002	-0.0338
4/2/2002	-0.0327
4/3/2002	-0.0406
4/4/2002	-0.0297
4/5/2002	-0.0317
4/6/2002	-0.0355
4/7/2002	-0.0345
4/8/2002	-0.0310
4/9/2002	-0.0346
4/10/2002	-0.0316
4/11/2002	-0.0334
4/12/2002	-0.0435
4/13/2002	-0.0370
4/14/2002	-0.0358
4/15/2002	-0.0343
4/16/2002	-0.0340
4/17/2002	-0.0388
4/18/2002	-0.0309
4/19/2002	-0.0294
4/20/2002	-0.0287
4/21/2002	-0.0353
4/22/2002	-0.0307
4/23/2002	-0.0322
4/24/2002	-0.0319
4/25/2002	-0.0311
4/26/2002	-0.0365
4/27/2002	-0.0304
4/28/2002	-0.0386

4/29/2002	-0.0343
4/30/2002	-0.0329
5/1/2002	-0.0339
5/2/2002	-0.0379
5/3/2002	-0.0318
5/4/2002	-0.0312
5/5/2002	-0.0311
5/16/2002	-0.0326
5/17/2002	-0.0301
5/18/2002	-0.0382
5/19/2002	-0.0278
5/20/2002	-0.0378
5/21/2002	-0.0404
5/22/2002	-0.0345
5/23/2002	-0.0373
5/24/2002	-0.0297
5/25/2002	-0.0320
5/26/2002	-0.0391
5/27/2002	-0.0377
5/28/2002	-0.0366
5/29/2002	-0.0333
5/30/2002	-0.0333
5/31/2002	-0.0349
6/1/2002	-0.0314
6/2/2002	-0.0357
6/3/2002	-0.0285
6/4/2002	-0.0277
6/5/2002	-0.0319
6/6/2002	-0.0272
6/7/2002	-0.0373
6/8/2002	-0.0335
6/9/2002	-0.0342
6/10/2002	-0.0304
6/11/2002	-0.0302
6/12/2002	-0.0292
6/13/2002	-0.0274
6/14/2002	-0.0271
6/15/2002	-0.0326
6/16/2002	-0.0296
6/17/2002	-0.0321
6/18/2002	-0.0326
6/19/2002	-0.0280
6/20/2002	-0.0276
6/21/2002	-0.0263
6/22/2002	-0.0241
6/23/2002	-0.0330
6/24/2002	-0.0224
6/25/2002	-0.0351
6/26/2002	-0.0292
6/27/2002	-0.0284

6/28/2002	-0.0288
6/29/2002	-0.0209
7/1/2002	-0.0301
7/2/2002	-0.0226
7/3/2002	-0.0295
7/4/2002	-0.0202
7/5/2002	-0.0205
7/6/2002	-0.0282
7/7/2002	-0.0218
7/8/2002	-0.0255
7/9/2002	-0.0126
7/10/2002	-0.0141
7/11/2002	-0.0191
7/12/2002	-0.0202
7/13/2002	-0.0192
7/14/2002	-0.0175
7/15/2002	-0.0200
7/16/2002	-0.0290
7/17/2002	-0.0241
7/18/2002	-0.0224
7/19/2002	-0.0277
7/20/2002	-0.0221
7/21/2002	-0.0208
7/22/2002	-0.0196
7/23/2002	-0.0185
7/24/2002	-0.0166
7/25/2002	-0.0231
7/26/2002	-0.0254
7/27/2002	-0.0239
7/28/2002	-0.0182
7/29/2002	-0.0296
7/30/2002	-0.0280
7/31/2002	-0.0371
8/1/2002	-0.0227
8/2/2002	-0.0209
8/3/2002	-0.0225
8/4/2002	-0.0198
8/5/2002	-0.0235
8/6/2002	-0.0168
8/7/2002	-0.0356
8/8/2002	-0.0191
8/9/2002	-0.0251
8/10/2002	-0.0231
8/11/2002	-0.0322
8/12/2002	-0.0223
8/13/2002	-0.0216
8/14/2002	-0.0246
8/15/2002	-0.0222
8/16/2002	-0.0215
8/17/2002	-0.0415

8/18/2002	-0.0196
8/19/2002	-0.0223
8/20/2002	-0.0231
8/21/2002	-0.0198
8/22/2002	-0.0177
8/23/2002	-0.0215
8/24/2002	-0.0317
8/25/2002	-0.0228
9/3/2002	-0.0211
9/4/2002	-0.0232
9/5/2002	-0.0331
9/6/2002	-0.0333
9/7/2002	-0.0270
9/8/2002	-0.0299
9/9/2002	-0.0353
9/10/2002	-0.0362
9/11/2002	-0.0342
9/12/2002	-0.0292
9/13/2002	-0.0280
9/14/2002	-0.0312
9/15/2002	-0.0290
9/16/2002	-0.0382
9/17/2002	-0.0238
9/18/2002	-0.0409
9/19/2002	-0.0321
9/20/2002	-0.0477
9/21/2002	-0.0244
9/22/2002	-0.0336
9/23/2002	-0.0361
9/24/2002	-0.0355
9/25/2002	-0.0363
9/26/2002	-0.0347
9/27/2002	-0.0315
9/28/2002	-0.0382
9/29/2002	-0.0381
9/30/2002	-0.0279
10/1/2002	-0.0388
10/2/2002	-0.0423
10/3/2002	-0.0414
10/4/2002	-0.0325
10/5/2002	-0.0376
10/6/2002	-0.0322
10/7/2002	-0.0320
10/8/2002	-0.0342
10/9/2002	-0.0376
10/10/2002	-0.0321
10/11/2002	-0.0285
10/12/2002	-0.0382
10/13/2002	-0.0368
10/14/2002	-0.0345

10/15/2002	-0.0332
10/16/2002	-0.0331
10/17/2002	-0.0331
10/18/2002	-0.0293
10/19/2002	-0.0342
10/20/2002	-0.0377
10/21/2002	-0.0426
10/22/2002	-0.0325
10/23/2002	-0.0335
10/24/2002	-0.0326
10/25/2002	-0.0334
10/26/2002	-0.0380
10/27/2002	-0.0368
10/28/2002	-0.0294
10/29/2002	-0.0337
10/30/2002	-0.0392
10/31/2002	-0.0316
11/1/2002	-0.0349
11/2/2002	-0.0308
11/3/2002	-0.0374
11/4/2002	-0.0330
11/5/2002	-0.0333
11/6/2002	-0.0369
11/7/2002	-0.0337
11/8/2002	-0.0308
11/9/2002	-0.0330
11/10/2002	-0.0322
11/11/2002	-0.0346
11/12/2002	-0.0345
11/13/2002	-0.0331
11/14/2002	-0.0336
11/15/2002	-0.0399
11/16/2002	-0.0334
11/17/2002	-0.0351
11/18/2002	-0.0415
11/19/2002	-0.0422
11/20/2002	-0.0418
11/21/2002	-0.0345
11/22/2002	-0.0394
11/23/2002	-0.0395
11/24/2002	-0.0362
11/25/2002	-0.0398
11/26/2002	-0.0350
11/27/2002	-0.0369
11/28/2002	-0.0354
11/29/2002	-0.0383
11/30/2002	-0.0399
12/1/2002	-0.0362
12/2/2002	-0.0344
12/3/2002	-0.0365

12/4/2002	-0.0335
12/5/2002	-0.0344
12/6/2002	-0.0367
12/7/2002	-0.0341
12/8/2002	-0.0284
12/9/2002	-0.0350
12/10/2002	-0.0351
12/11/2002	-0.0362
12/12/2002	-0.0328
12/13/2002	-0.0367
12/14/2002	-0.0347
12/15/2002	-0.0379
12/16/2002	-0.0303
12/17/2002	-0.0331
12/18/2002	-0.0279
12/19/2002	-0.0373
12/20/2002	-0.0339
12/21/2002	-0.0341
12/22/2002	-0.0332
12/23/2002	-0.0379
12/24/2002	-0.0338
12/25/2002	-0.0363
12/26/2002	-0.0282
12/27/2002	-0.0303
12/28/2002	-0.0270
12/29/2002	-0.0313
12/30/2002	-0.0316
12/31/2002	-0.0235
1/1/2003	-0.0255
1/2/2003	-0.0290
1/3/2003	-0.0279
1/4/2003	-0.0288
1/5/2003	-0.0249
1/6/2003	-0.0248
1/7/2003	-0.0228
1/8/2003	-0.0194
1/9/2003	-0.0237
1/10/2003	-0.0274
1/11/2003	-0.0195
1/12/2003	-0.0223
1/13/2003	-0.0265
1/14/2003	-0.0297
1/15/2003	-0.0273
1/16/2003	-0.0284
1/17/2003	-0.0254
1/18/2003	-0.0249
1/19/2003	-0.0306
1/20/2003	-0.0300
1/21/2003	-0.0348
1/23/2003	-0.0272

1/24/2003	-0.0301
1/25/2003	-0.0260
1/26/2003	-0.0275
1/27/2003	-0.0252
1/28/2003	-0.0281
1/29/2003	-0.0213
1/30/2003	-0.0212
1/31/2003	-0.0261
2/1/2003	-0.0254
2/2/2003	-0.0262
2/3/2003	-0.0295
2/4/2003	-0.0309
2/5/2003	-0.0274
2/6/2003	-0.0252
2/7/2003	-0.0303
2/8/2003	-0.0312
2/9/2003	-0.0348
2/10/2003	-0.0293
2/11/2003	-0.0321
2/13/2003	-0.0329
2/14/2003	-0.0296
2/15/2003	-0.0305
2/16/2003	-0.0257
2/21/2003	-0.0333
2/22/2003	-0.0361
2/23/2003	-0.0300
2/24/2003	-0.0333
2/25/2003	-0.0333
2/26/2003	-0.0348
2/27/2003	-0.0372
2/28/2003	-0.0332
3/1/2003	-0.0293
3/2/2003	-0.0345
3/3/2003	-0.0309
3/4/2003	-0.0340
3/6/2003	-0.0349
3/7/2003	-0.0308
3/8/2003	-0.0346
3/9/2003	-0.0347
3/10/2003	-0.0373
3/11/2003	-0.0332
3/12/2003	-0.0311
3/13/2003	-0.0290
3/14/2003	-0.0329
3/15/2003	-0.0313
3/16/2003	-0.0309
3/17/2003	-0.0312
3/18/2003	-0.0336
3/19/2003	-0.0321
3/20/2003	-0.0336

3/21/2003	-0.0284
3/22/2003	-0.0279
3/23/2003	-0.0279
3/24/2003	-0.0314
3/25/2003	-0.0290
3/26/2003	-0.0360
3/27/2003	-0.0327
3/28/2003	-0.0341
3/29/2003	-0.0336
3/30/2003	-0.0361
3/31/2003	-0.0299
4/1/2003	-0.0302
4/2/2003	-0.0325
4/3/2003	-0.0292
4/4/2003	-0.0309
4/5/2003	-0.0305
4/6/2003	-0.0238
4/7/2003	-0.0297
4/8/2003	-0.0364
4/9/2003	-0.0340
4/10/2003	-0.0382
4/11/2003	-0.0271
4/12/2003	-0.0368
4/13/2003	-0.0303
4/14/2003	-0.0330
4/15/2003	-0.0296
4/16/2003	-0.0284
4/17/2003	-0.0267
4/18/2003	-0.0296
4/19/2003	-0.0284
4/20/2003	-0.0355
4/21/2003	-0.0316
4/22/2003	-0.0243
4/23/2003	-0.0376
4/24/2003	-0.0334
4/25/2003	-0.0353
4/26/2003	-0.0320
4/27/2003	-0.0334
4/28/2003	-0.0363
4/29/2003	-0.0346
4/30/2003	-0.0367
5/1/2003	-0.0297
5/2/2003	-0.0321
5/3/2003	-0.0322
5/4/2003	-0.0326
5/5/2003	-0.0306
5/6/2003	-0.0330
5/8/2003	-0.0311
5/9/2003	-0.0286
5/10/2003	-0.0286

5/11/2003	-0.0307
5/12/2003	-0.0303
5/13/2003	-0.0318
5/14/2003	-0.0276
5/15/2003	-0.0295
5/16/2003	-0.0315
5/17/2003	-0.0323
5/18/2003	-0.0325
5/19/2003	-0.0328
5/20/2003	-0.0309
5/21/2003	-0.0344
5/22/2003	-0.0229
5/23/2003	-0.0231
5/24/2003	-0.0340
5/25/2003	-0.0317
5/26/2003	-0.0251
5/27/2003	-0.0301
5/28/2003	-0.0275
5/29/2003	-0.0329
5/30/2003	-0.0260
5/31/2003	-0.0289
6/1/2003	-0.0315
6/2/2003	-0.0296
6/3/2003	-0.0323
6/4/2003	-0.0344
6/10/2003	-0.0328
6/11/2003	-0.0259
6/13/2003	-0.0293
6/14/2003	-0.0267
6/15/2003	-0.0195
6/16/2003	-0.0294
6/17/2003	-0.0260
6/18/2003	-0.0302
6/19/2003	-0.0296
6/20/2003	-0.0280
6/21/2003	-0.0336
6/22/2003	-0.0295
6/23/2003	-0.0325
6/25/2003	-0.0303
6/26/2003	-0.0297
6/27/2003	-0.0320
6/28/2003	-0.0292
6/29/2003	-0.0356
6/30/2003	-0.0308
7/1/2003	-0.0261
7/2/2003	-0.0407
7/3/2003	-0.0393
7/4/2003	-0.0367
7/5/2003	-0.0302
7/6/2003	-0.0327

7/7/2003	-0.0396
7/8/2003	-0.0317
7/9/2003	-0.0334
7/10/2003	-0.0302
7/11/2003	-0.0251
7/12/2003	-0.0276
7/13/2003	-0.0231
7/14/2003	-0.0331
7/15/2003	-0.0329
7/16/2003	-0.0304
7/17/2003	-0.0258
7/18/2003	-0.0263
7/19/2003	-0.0215
7/20/2003	-0.0295
7/22/2003	-0.0310
7/23/2003	-0.0265
7/24/2003	-0.0336
7/25/2003	-0.0241
7/26/2003	-0.0317
7/27/2003	-0.0334
7/28/2003	-0.0283
7/29/2003	-0.0267
7/30/2003	-0.0223
7/31/2003	-0.0224
8/1/2003	-0.0250
8/2/2003	-0.0256
8/3/2003	-0.0117
8/4/2003	-0.0232
8/5/2003	-0.0122
8/6/2003	-0.0274
8/7/2003	-0.0270
8/8/2003	-0.0296
8/9/2003	-0.0332
8/10/2003	-0.0323
8/11/2003	-0.0336
8/12/2003	-0.0149
8/13/2003	-0.0232
8/14/2003	-0.0324
8/15/2003	-0.0294
8/16/2003	-0.0294
8/17/2003	-0.0332
8/18/2003	-0.0323
8/19/2003	-0.0302
8/20/2003	-0.0294
8/21/2003	-0.0374
8/22/2003	-0.0289
8/23/2003	-0.0342
8/24/2003	-0.0341
9/1/2003	-0.0303
9/2/2003	-0.0312

9/3/2003	-0.0280
9/4/2003	-0.0333
9/5/2003	-0.0338
9/6/2003	-0.0307
9/7/2003	-0.0324
9/8/2003	-0.0301
9/9/2003	-0.0343
9/10/2003	-0.0303
9/11/2003	-0.0363
9/12/2003	-0.0322
9/13/2003	-0.0315
9/14/2003	-0.0264
9/15/2003	-0.0331
9/16/2003	-0.0349
9/17/2003	-0.0315
9/18/2003	-0.0390
9/19/2003	-0.0328
9/20/2003	-0.0361
9/21/2003	-0.0319
9/22/2003	-0.0374
9/23/2003	-0.0317
9/24/2003	-0.0305
9/25/2003	-0.0308
9/26/2003	-0.0297
9/27/2003	-0.0310
9/28/2003	-0.0325
9/29/2003	-0.0184
9/30/2003	-0.0284
10/1/2003	-0.0303
10/2/2003	-0.0313
10/3/2003	-0.0273
10/4/2003	-0.0267
10/5/2003	-0.0276
10/6/2003	-0.0326
10/7/2003	-0.0225
10/8/2003	-0.0338
10/9/2003	-0.0321
10/10/2003	-0.0324
10/11/2003	-0.0315
10/12/2003	-0.0305
10/13/2003	-0.0342
10/14/2003	-0.0350
10/15/2003	-0.0246
10/16/2003	-0.0328
10/17/2003	-0.0301
10/18/2003	-0.0307
10/19/2003	-0.0322
10/20/2003	-0.0306
10/21/2003	-0.0258
10/22/2003	-0.0360

10/23/2003	-0.0281
10/24/2003	-0.0280
10/25/2003	-0.0360
10/26/2003	-0.0363
10/27/2003	-0.0283
10/28/2003	-0.0330
10/29/2003	-0.0315
10/30/2003	-0.0309
10/31/2003	-0.0365
11/1/2003	-0.0366
11/2/2003	-0.0358
11/3/2003	-0.0324
11/4/2003	-0.0280
11/5/2003	-0.0309
11/6/2003	-0.0299
11/7/2003	-0.0335
11/8/2003	-0.0316
11/9/2003	-0.0321
11/10/2003	-0.0319
11/11/2003	-0.0302
11/12/2003	-0.0329
11/13/2003	-0.0299
11/14/2003	-0.0347
11/15/2003	-0.0314
11/16/2003	-0.0365
11/17/2003	-0.0296
11/18/2003	-0.0339
11/19/2003	-0.0305
11/20/2003	-0.0423
11/21/2003	-0.0347
11/22/2003	-0.0311
11/23/2003	-0.0360
11/24/2003	-0.0297
11/25/2003	-0.0280
11/26/2003	-0.0280
11/27/2003	-0.0297
11/28/2003	-0.0223
11/29/2003	-0.0360
11/30/2003	-0.0329
12/1/2003	-0.0309
12/2/2003	-0.0329
12/3/2003	-0.0291
12/4/2003	-0.0277
12/5/2003	-0.0304
12/6/2003	-0.0289
12/7/2003	-0.0317
12/8/2003	-0.0322
12/9/2003	-0.0329
12/10/2003	-0.0297
12/11/2003	-0.0273

12/12/2003	-0.0299
12/13/2003	-0.0371
12/14/2003	-0.0261
12/15/2003	-0.0265
12/16/2003	-0.0363
12/17/2003	-0.0293
12/18/2003	-0.0313
12/19/2003	-0.0302
12/20/2003	-0.0300
12/21/2003	-0.0308
12/22/2003	-0.0281
12/23/2003	-0.0237
12/24/2003	-0.0249
12/25/2003	-0.0284
12/26/2003	-0.0302
12/27/2003	-0.0331
12/28/2003	-0.0328
12/29/2003	-0.0327
12/30/2003	-0.0303
12/31/2003	-0.0315
1/1/2004	-0.0303
1/2/2004	-0.0319
1/3/2004	-0.0345
1/4/2004	-0.0318
1/5/2004	-0.0304
1/6/2004	-0.0359
1/7/2004	-0.0303
1/8/2004	-0.0362
1/9/2004	-0.0373
1/10/2004	-0.0323
1/11/2004	-0.0357
1/12/2004	-0.0331
1/13/2004	-0.0326
1/14/2004	-0.0290
1/15/2004	-0.0289
1/16/2004	-0.0393
1/17/2004	-0.0339
1/18/2004	-0.0297
1/19/2004	-0.0248
1/20/2004	-0.0292
1/21/2004	-0.0255
1/22/2004	-0.0290
1/23/2004	-0.0317
1/24/2004	-0.0277
1/25/2004	-0.0306
1/26/2004	-0.0317
1/27/2004	-0.0297
1/28/2004	-0.0272
1/29/2004	-0.0318
1/30/2004	-0.0351

1/31/2004	-0.0385
2/1/2004	-0.0349
2/2/2004	-0.0347
2/3/2004	-0.0348
2/4/2004	-0.0334
2/9/2004	-0.0318
2/10/2004	-0.0340
2/11/2004	-0.0318
2/12/2004	-0.0317
2/13/2004	-0.0384
2/14/2004	-0.0363
2/15/2004	-0.0339
2/16/2004	-0.0355
2/17/2004	-0.0342
2/18/2004	-0.0335
2/19/2004	-0.0350
2/20/2004	-0.0344
2/21/2004	-0.0369
2/22/2004	-0.0353
2/23/2004	-0.0323
2/24/2004	-0.0312
2/25/2004	-0.0391
2/26/2004	-0.0336
2/27/2004	-0.0411
2/28/2004	-0.0413
2/29/2004	-0.0422
3/1/2004	-0.0448
3/2/2004	-0.0397
3/3/2004	-0.0363
3/4/2004	-0.0327
3/5/2004	-0.0390
3/6/2004	-0.0434
3/7/2004	-0.0418
3/9/2004	-0.0437
3/10/2004	-0.0460
3/11/2004	-0.0456
3/12/2004	-0.0450
3/13/2004	-0.0429
3/14/2004	-0.0423
3/15/2004	-0.0437
3/16/2004	-0.0421
3/17/2004	-0.0398
3/18/2004	-0.0412
3/19/2004	-0.0401
3/20/2004	-0.0414
3/21/2004	-0.0396
3/22/2004	-0.0369
3/23/2004	-0.0406
3/24/2004	-0.0356
3/25/2004	-0.0341

3/26/2004	-0.0355
3/27/2004	-0.0424
3/28/2004	-0.0396
3/29/2004	-0.0378
3/30/2004	-0.0395
3/31/2004	-0.0413
4/1/2004	-0.0442
4/2/2004	-0.0463
4/3/2004	-0.0425
4/4/2004	-0.0452
4/5/2004	-0.0384
4/6/2004	-0.0428
4/7/2004	-0.0416
4/8/2004	-0.0397
4/9/2004	-0.0411
4/10/2004	-0.0391
4/11/2004	-0.0421
4/12/2004	-0.0410
4/13/2004	-0.0419
4/14/2004	-0.0451
4/15/2004	-0.0426
4/17/2004	-0.0484
4/18/2004	-0.0490
4/21/2004	-0.0396
4/22/2004	-0.0412
4/23/2004	-0.0446
4/24/2004	-0.0445
4/25/2004	-0.0369
4/26/2004	-0.0333
4/27/2004	-0.0410
4/28/2004	-0.0435
4/29/2004	-0.0420
4/30/2004	-0.0443
5/1/2004	-0.0438
5/2/2004	-0.0438
5/3/2004	-0.0454
5/4/2004	-0.0458
5/5/2004	-0.0468
5/6/2004	-0.0459
5/7/2004	-0.0525
5/8/2004	-0.0407
5/9/2004	-0.0401
5/10/2004	-0.0411
5/11/2004	-0.0446
5/12/2004	-0.0409
5/13/2004	-0.0381
5/14/2004	-0.0369
5/15/2004	-0.0372
5/16/2004	-0.0426
5/17/2004	-0.0338

5/18/2004	-0.0370
5/19/2004	-0.0395
5/20/2004	-0.0394
5/21/2004	-0.0492
5/22/2004	-0.0425
5/23/2004	-0.0346
5/24/2004	-0.0409
5/25/2004	-0.0406
5/26/2004	-0.0442
5/27/2004	-0.0418
5/28/2004	-0.0454
5/29/2004	-0.0401
5/30/2004	-0.0385
5/31/2004	-0.0420
6/1/2004	-0.0452
6/2/2004	-0.0408
6/3/2004	-0.0386
6/4/2004	-0.0417
6/5/2004	-0.0405
6/6/2004	-0.0504
6/7/2004	-0.0436
6/8/2004	-0.0452
6/9/2004	-0.0411
6/10/2004	-0.0415
6/11/2004	-0.0405
6/12/2004	-0.0448
6/13/2004	-0.0422
6/14/2004	-0.0471
6/15/2004	-0.0448
6/16/2004	-0.0390
6/17/2004	-0.0394
6/18/2004	-0.0440
6/19/2004	-0.0442
6/20/2004	-0.0468
6/21/2004	-0.0439
6/22/2004	-0.0452
6/23/2004	-0.0412
6/24/2004	-0.0333
6/25/2004	-0.0453
6/26/2004	-0.0444
6/27/2004	-0.0427
6/28/2004	-0.0402
6/29/2004	-0.0415
6/30/2004	-0.0352
7/1/2004	-0.0439
7/2/2004	-0.0437
7/3/2004	-0.0399
7/4/2004	-0.0443
7/5/2004	-0.0380
7/6/2004	-0.0311

7/7/2004	-0.0454
7/8/2004	-0.0379
7/9/2004	-0.0397
7/10/2004	-0.0448
7/11/2004	-0.0345
7/12/2004	-0.0411
7/13/2004	-0.0385
7/14/2004	-0.0325
7/15/2004	-0.0447
7/16/2004	-0.0451
7/17/2004	-0.0471
7/18/2004	-0.0469
7/19/2004	-0.0477
7/20/2004	-0.0441
7/21/2004	-0.0423
7/22/2004	-0.0384
7/23/2004	-0.0366
7/24/2004	-0.0357
7/25/2004	-0.0423
7/26/2004	-0.0373
7/27/2004	-0.0436
7/28/2004	-0.0434
7/29/2004	-0.0427
7/30/2004	-0.0364
7/31/2004	-0.0503
8/1/2004	-0.0428
8/2/2004	-0.0393
8/3/2004	-0.0421
8/4/2004	-0.0451
8/6/2004	-0.0400
8/7/2004	-0.0538
8/8/2004	-0.0455
8/9/2004	-0.0461
8/10/2004	-0.0430
8/11/2004	-0.0434
8/12/2004	-0.0527
8/13/2004	-0.0355
8/14/2004	-0.0462
8/15/2004	-0.0423
8/16/2004	-0.0470
8/17/2004	-0.0478
8/18/2004	-0.0441
8/19/2004	-0.0502
8/20/2004	-0.0507
8/21/2004	-0.0459
8/22/2004	-0.0437
8/23/2004	-0.0466
8/24/2004	-0.0488
8/25/2004	-0.0428
8/26/2004	-0.0458

8/27/2004	-0.0433
8/28/2004	-0.0454
8/29/2004	-0.0443
8/30/2004	-0.0499
8/31/2004	-0.0444
9/1/2004	-0.0421
9/2/2004	-0.0465
9/3/2004	-0.0485
9/4/2004	-0.0520
9/5/2004	-0.0443
9/7/2004	-0.0484
9/8/2004	-0.0465
9/9/2004	-0.0425
9/10/2004	-0.0468
9/11/2004	-0.0461
9/12/2004	-0.0474
9/13/2004	-0.0467
9/14/2004	-0.0514
9/15/2004	-0.0469
9/16/2004	-0.0408
9/17/2004	-0.0497
9/18/2004	-0.0377
9/19/2004	-0.0449
9/20/2004	-0.0475
9/21/2004	-0.0439
9/22/2004	-0.0433
9/23/2004	-0.0457
9/24/2004	-0.0434
9/25/2004	-0.0472
9/26/2004	-0.0427
9/27/2004	-0.0458
9/28/2004	-0.0480
9/29/2004	-0.0459
9/30/2004	-0.0463
10/1/2004	-0.0464
10/2/2004	-0.0443
10/3/2004	-0.0440
10/4/2004	-0.0484
10/5/2004	-0.0424
10/6/2004	-0.0419
10/7/2004	-0.0460
10/8/2004	-0.0416
10/9/2004	-0.0418
10/10/2004	-0.0365
10/11/2004	-0.0524
10/12/2004	-0.0459
10/13/2004	-0.0407
10/14/2004	-0.0466
10/15/2004	-0.0438
10/16/2004	-0.0456

10/17/2004	-0.0505
10/18/2004	-0.0468
10/19/2004	-0.0454
10/21/2004	-0.0433
10/22/2004	-0.0426
10/23/2004	-0.0429
10/24/2004	-0.0510
10/25/2004	-0.0453
10/26/2004	-0.0400
10/27/2004	-0.0483
10/28/2004	-0.0475
10/29/2004	-0.0439
10/30/2004	-0.0444
10/31/2004	-0.0446
11/1/2004	-0.0423
11/2/2004	-0.0428
11/3/2004	-0.0398
11/4/2004	-0.0381
11/5/2004	-0.0423
11/6/2004	-0.0417
11/7/2004	-0.0412
11/8/2004	-0.0426
11/9/2004	-0.0416
11/10/2004	-0.0398
11/11/2004	-0.0448
11/12/2004	-0.0428
11/13/2004	-0.0374
11/14/2004	-0.0396
11/15/2004	-0.0433
11/16/2004	-0.0451
11/17/2004	-0.0450
11/18/2004	-0.0465
11/19/2004	-0.0368
11/20/2004	-0.0390
11/21/2004	-0.0443
11/22/2004	-0.0382
11/23/2004	-0.0443
11/24/2004	-0.0414
11/25/2004	-0.0445
11/26/2004	-0.0466
11/27/2004	-0.0444
11/28/2004	-0.0485
11/29/2004	-0.0510
11/30/2004	-0.0441
12/1/2004	-0.0417
12/2/2004	-0.0418
12/3/2004	-0.0455
12/4/2004	-0.0440
12/5/2004	-0.0440
12/6/2004	-0.0421

12/7/2004	-0.0490
12/8/2004	-0.0435
12/9/2004	-0.0457
12/10/2004	-0.0397
12/11/2004	-0.0417
12/12/2004	-0.0425
12/13/2004	-0.0412
12/14/2004	-0.0413
12/15/2004	-0.0404
12/16/2004	-0.0417
12/17/2004	-0.0413
12/18/2004	-0.0445
12/19/2004	-0.0395
12/20/2004	-0.0428
12/21/2004	-0.0434
12/22/2004	-0.0444
12/23/2004	-0.0447
12/24/2004	-0.0410
12/25/2004	-0.0425
12/26/2004	-0.0361
12/27/2004	-0.0387
12/28/2004	-0.0425
12/29/2004	-0.0413
12/30/2004	-0.0388
1/1/2005	-0.0442
1/2/2005	-0.0413
1/3/2005	-0.0422
1/4/2005	-0.0395
1/5/2005	-0.0425
1/6/2005	-0.0418
1/7/2005	-0.0377
1/8/2005	-0.0453
1/9/2005	-0.0415
1/10/2005	-0.0414
1/11/2005	-0.0412
1/12/2005	-0.0376
1/13/2005	-0.0348
1/14/2005	-0.0363
1/15/2005	-0.0353
1/16/2005	-0.0463
1/17/2005	-0.0448
1/18/2005	-0.0415
1/19/2005	-0.0368
1/20/2005	-0.0460
1/21/2005	-0.0366
1/22/2005	-0.0398
1/23/2005	-0.0383
1/24/2005	-0.0322
1/25/2005	-0.0307
1/26/2005	-0.0329

1/27/2005	-0.0407
1/28/2005	-0.0361
1/29/2005	-0.0395
1/30/2005	-0.0376
1/31/2005	-0.0399
2/1/2005	-0.0427
2/2/2005	-0.0419
2/3/2005	-0.0402
2/4/2005	-0.0406
2/5/2005	-0.0389
2/6/2005	-0.0414
2/7/2005	-0.0426
2/8/2005	-0.0440
2/9/2005	-0.0405
2/10/2005	-0.0388
2/11/2005	-0.0429
2/12/2005	-0.0420
2/13/2005	-0.0383
2/14/2005	-0.0365
2/15/2005	-0.0375
2/16/2005	-0.0326
2/17/2005	-0.0361
2/18/2005	-0.0379
2/19/2005	-0.0389
2/20/2005	-0.0415
2/21/2005	-0.0391
2/22/2005	-0.0412
2/23/2005	-0.0424
2/24/2005	-0.0443
2/25/2005	-0.0414
2/26/2005	-0.0404
2/27/2005	-0.0408
2/28/2005	-0.0395
3/1/2005	-0.0429
3/2/2005	-0.0416
3/3/2005	-0.0402
3/4/2005	-0.0414
3/5/2005	-0.0443
3/6/2005	-0.0348
3/7/2005	-0.0413
3/8/2005	-0.0423
3/9/2005	-0.0418
3/10/2005	-0.0434
3/11/2005	-0.0451
3/12/2005	-0.0487
3/13/2005	-0.0421
3/14/2005	-0.0440
3/15/2005	-0.0400
3/16/2005	-0.0457
3/17/2005	-0.0400

3/18/2005	-0.0410
3/19/2005	-0.0383
3/20/2005	-0.0394
3/21/2005	-0.0432
3/22/2005	-0.0434
3/23/2005	-0.0463
3/24/2005	-0.0441
3/25/2005	-0.0447
3/26/2005	-0.0460
3/27/2005	-0.0438
3/28/2005	-0.0446
3/29/2005	-0.0435
3/30/2005	-0.0457
3/31/2005	-0.0377
4/1/2005	-0.0431
4/2/2005	-0.0435
4/3/2005	-0.0476
4/4/2005	-0.0480
4/5/2005	-0.0489
4/6/2005	-0.0489
4/7/2005	-0.0509
4/8/2005	-0.0479
4/9/2005	-0.0425
4/10/2005	-0.0490
4/11/2005	-0.0388
4/12/2005	-0.0444
4/13/2005	-0.0478
4/14/2005	-0.0443
4/15/2005	-0.0469
4/16/2005	-0.0468
4/17/2005	-0.0511
4/18/2005	-0.0484
4/19/2005	-0.0447
4/20/2005	-0.0410
4/21/2005	-0.0446
4/22/2005	-0.0438
4/23/2005	-0.0443
4/24/2005	-0.0475
4/25/2005	-0.0475
4/26/2005	-0.0420
4/27/2005	-0.0427
4/28/2005	-0.0440
4/29/2005	-0.0425
4/30/2005	-0.0451
5/1/2005	-0.0444
5/2/2005	-0.0404
5/4/2005	-0.0401
5/5/2005	-0.0359
5/6/2005	-0.0346
5/7/2005	-0.0418

5/8/2005	-0.0416
5/9/2005	-0.0405
5/10/2005	-0.0442
5/11/2005	-0.0403
5/12/2005	-0.0409
5/13/2005	-0.0429
5/14/2005	-0.0417
5/15/2005	-0.0385
5/16/2005	-0.0389
5/17/2005	-0.0412
5/18/2005	-0.0442
5/19/2005	-0.0391
5/20/2005	-0.0402
5/21/2005	-0.0411
5/22/2005	-0.0410
5/23/2005	-0.0397
5/24/2005	-0.0352
5/25/2005	-0.0390
5/26/2005	-0.0384
5/27/2005	-0.0431
5/28/2005	-0.0477
5/29/2005	-0.0461
5/30/2005	-0.0363
5/31/2005	-0.0346
6/1/2005	-0.0413
6/2/2005	-0.0432
6/3/2005	-0.0402
6/4/2005	-0.0357
6/5/2005	-0.0368
6/7/2005	-0.0353
6/8/2005	-0.0288
6/9/2005	-0.0338
6/10/2005	-0.0374
6/11/2005	-0.0394
6/12/2005	-0.0511
6/13/2005	-0.0414
6/14/2005	-0.0406
6/15/2005	-0.0400
6/16/2005	-0.0425
6/17/2005	-0.0390
6/18/2005	-0.0404
6/19/2005	-0.0382
6/20/2005	-0.0426
6/21/2005	-0.0360
6/22/2005	-0.0429
6/23/2005	-0.0448
6/24/2005	-0.0378
6/25/2005	-0.0382
6/26/2005	-0.0335
6/27/2005	-0.0393

6/28/2005	-0.0328
6/29/2005	-0.0416
6/30/2005	-0.0348
7/1/2005	-0.0410
7/2/2005	-0.0381
7/3/2005	-0.0382
7/4/2005	-0.0389
7/5/2005	-0.0373
7/6/2005	-0.0346
7/7/2005	-0.0406
7/8/2005	-0.0434
7/9/2005	-0.0428
7/10/2005	-0.0347
7/11/2005	-0.0328
7/12/2005	-0.0387
7/13/2005	-0.0327
7/14/2005	-0.0442
7/15/2005	-0.0398
7/16/2005	-0.0398
7/17/2005	-0.0432
7/18/2005	-0.0421
7/19/2005	-0.0334
7/20/2005	-0.0356
7/21/2005	-0.0384
7/22/2005	-0.0376
7/23/2005	-0.0464
7/24/2005	-0.0415
7/25/2005	-0.0404
7/26/2005	-0.0436
7/27/2005	-0.0451
7/28/2005	-0.0392
7/29/2005	-0.0391
7/30/2005	-0.0341
7/31/2005	-0.0426
8/1/2005	-0.0366
8/2/2005	-0.0407
8/3/2005	-0.0406
8/4/2005	-0.0361
8/5/2005	-0.0372
8/6/2005	-0.0427
8/7/2005	-0.0425
8/8/2005	-0.0412
8/9/2005	-0.0396
8/10/2005	-0.0404
8/11/2005	-0.0402
8/12/2005	-0.0357
8/13/2005	-0.0343
8/14/2005	-0.0330
8/15/2005	-0.0393
8/16/2005	-0.0339

8/17/2005	-0.0396
8/18/2005	-0.0377
8/19/2005	-0.0428
8/20/2005	-0.0399
8/21/2005	-0.0373
8/22/2005	-0.0401
8/23/2005	-0.0365
8/24/2005	-0.0400
8/25/2005	-0.0386
8/26/2005	-0.0457
8/27/2005	-0.0358
8/28/2005	-0.0400
8/29/2005	-0.0352
8/30/2005	-0.0379
8/31/2005	-0.0388
9/1/2005	-0.0398
9/2/2005	-0.0404
9/3/2005	-0.0392
9/4/2005	-0.0420
9/5/2005	-0.0421
9/6/2005	-0.0405
9/7/2005	-0.0347
9/8/2005	-0.0375
9/9/2005	-0.0434
9/10/2005	-0.0343
9/11/2005	-0.0408
9/12/2005	-0.0396
9/13/2005	-0.0379
9/14/2005	-0.0354
9/15/2005	-0.0394
9/16/2005	-0.0389
9/17/2005	-0.0373
9/18/2005	-0.0385
9/24/2005	-0.0384
9/25/2005	-0.0402
9/27/2005	-0.0343
9/28/2005	-0.0388
9/29/2005	-0.0346
9/30/2005	-0.0384
10/1/2005	-0.0392
10/2/2005	-0.0316
10/3/2005	-0.0402
10/4/2005	-0.0432
10/5/2005	-0.0405
10/6/2005	-0.0390
10/7/2005	-0.0425
10/8/2005	-0.0457
10/9/2005	-0.0338
10/10/2005	-0.0399
10/11/2005	-0.0449

10/12/2005	-0.0428
10/13/2005	-0.0379
10/14/2005	-0.0430
10/15/2005	-0.0403
10/16/2005	-0.0393
10/17/2005	-0.0387
10/18/2005	-0.0362
10/19/2005	-0.0368
10/20/2005	-0.0378
10/21/2005	-0.0386
10/22/2005	-0.0487
10/23/2005	-0.0355
10/24/2005	-0.0400
10/25/2005	-0.0463
10/26/2005	-0.0392
10/27/2005	-0.0423
10/28/2005	-0.0393
10/29/2005	-0.0392
10/30/2005	-0.0400
10/31/2005	-0.0436
11/1/2005	-0.0364
11/2/2005	-0.0443
11/3/2005	-0.0396
11/4/2005	-0.0394
11/5/2005	-0.0356
11/6/2005	-0.0422
11/7/2005	-0.0406
11/8/2005	-0.0376
11/9/2005	-0.0378
11/10/2005	-0.0388
11/11/2005	-0.0397
11/12/2005	-0.0379
11/13/2005	-0.0373
11/14/2005	-0.0390
11/15/2005	-0.0375
11/16/2005	-0.0338
11/17/2005	-0.0357
11/19/2005	-0.0390
11/20/2005	-0.0327
11/21/2005	-0.0375
11/22/2005	-0.0357
11/23/2005	-0.0358
11/24/2005	-0.0385
11/25/2005	-0.0332
11/26/2005	-0.0287
11/27/2005	-0.0329
11/28/2005	-0.0356
11/29/2005	-0.0379
11/30/2005	-0.0328
12/1/2005	-0.0347

12/2/2005	-0.0414
12/3/2005	-0.0374
12/4/2005	-0.0390
12/5/2005	-0.0264
12/6/2005	-0.0356
12/7/2005	-0.0362
12/8/2005	-0.0360
12/9/2005	-0.0342
12/10/2005	-0.0300
12/11/2005	-0.0310
12/12/2005	-0.0378
12/13/2005	-0.0296
12/14/2005	-0.0324
12/15/2005	-0.0320
12/16/2005	-0.0332
12/17/2005	-0.0303
12/18/2005	-0.0345
12/19/2005	-0.0356
12/20/2005	-0.0362
12/21/2005	-0.0320
12/22/2005	-0.0298
12/23/2005	-0.0280
12/24/2005	-0.0332
12/25/2005	-0.0328
12/26/2005	-0.0299
12/27/2005	-0.0228
12/28/2005	-0.0269
12/29/2005	-0.0230
12/30/2005	-0.0303
12/31/2005	-0.0357
1/1/2006	-0.0342
1/2/2006	-0.0270
1/3/2006	-0.0252
1/10/2006	-0.0303
1/11/2006	-0.0313
1/12/2006	-0.0268
1/13/2006	-0.0260
1/14/2006	-0.0273
1/15/2006	-0.0286
1/16/2006	-0.0293
1/17/2006	-0.0360
1/18/2006	-0.0237
1/19/2006	-0.0218
1/20/2006	-0.0287
1/21/2006	-0.0261
1/22/2006	-0.0256
1/23/2006	-0.0220
1/24/2006	-0.0246
1/25/2006	-0.0270
1/26/2006	-0.0312

1/28/2006	-0.0274
1/29/2006	-0.0267
1/30/2006	-0.0303
1/31/2006	-0.0283
2/1/2006	-0.0274
2/2/2006	-0.0244
2/3/2006	-0.0276
2/4/2006	-0.0225
2/5/2006	-0.0249
2/6/2006	-0.0250
2/7/2006	-0.0178
2/8/2006	-0.0270
2/9/2006	-0.0209
2/11/2006	-0.0242
2/13/2006	-0.0283
2/15/2006	-0.0308
2/16/2006	-0.0317
2/17/2006	-0.0331
2/18/2006	-0.0305
2/19/2006	-0.0330
2/20/2006	-0.0307
2/21/2006	-0.0276
2/22/2006	-0.0286
2/23/2006	-0.0227
2/24/2006	-0.0202
2/25/2006	-0.0302
2/26/2006	-0.0262
2/27/2006	-0.0273
2/28/2006	-0.0382
3/1/2006	-0.0299
3/2/2006	-0.0325
3/3/2006	-0.0315
3/4/2006	-0.0271
3/5/2006	-0.0203
3/6/2006	-0.0204
3/7/2006	-0.0251
3/8/2006	-0.0288
3/9/2006	-0.0289
3/10/2006	-0.0291
3/11/2006	-0.0241
3/12/2006	-0.0190
3/13/2006	-0.0170
3/14/2006	-0.0264
3/15/2006	-0.0268
3/16/2006	-0.0285
3/17/2006	-0.0272
3/18/2006	-0.0270
3/19/2006	-0.0293
3/20/2006	-0.0286
3/21/2006	-0.0274

3/22/2006	-0.0235
3/23/2006	-0.0295
3/24/2006	-0.0226
3/25/2006	-0.0285
3/26/2006	-0.0276
3/27/2006	-0.0283
3/28/2006	-0.0259
3/29/2006	-0.0273
3/30/2006	-0.0243
3/31/2006	-0.0245
4/1/2006	-0.0246
4/2/2006	-0.0224
4/3/2006	-0.0202
4/4/2006	-0.0252
4/5/2006	-0.0203
4/6/2006	-0.0233
4/7/2006	-0.0246
4/8/2006	-0.0259
4/9/2006	-0.0246
4/10/2006	-0.0167
4/11/2006	-0.0213
4/12/2006	-0.0241
4/13/2006	-0.0255
4/14/2006	-0.0268
4/15/2006	-0.0260
4/16/2006	-0.0244
4/17/2006	-0.0220
4/18/2006	-0.0221
4/19/2006	-0.0251
4/20/2006	-0.0260
4/21/2006	-0.0234
4/22/2006	-0.0258
4/23/2006	-0.0188
4/24/2006	-0.0223
4/25/2006	-0.0176
4/26/2006	-0.0198
4/27/2006	-0.0167
4/28/2006	-0.0126
4/29/2006	-0.0164
4/30/2006	-0.0156
5/1/2006	-0.0228
5/2/2006	-0.0186
5/3/2006	-0.0222
5/4/2006	-0.0170
5/5/2006	-0.0216
5/6/2006	-0.0176
5/7/2006	-0.0213
5/8/2006	-0.0230
5/9/2006	-0.0201
5/10/2006	-0.0202

5/11/2006	-0.0175
5/13/2006	-0.0219
5/14/2006	-0.0191
5/15/2006	-0.0180
5/16/2006	-0.0177
5/17/2006	-0.0209
5/18/2006	-0.0205
5/19/2006	-0.0123
5/20/2006	-0.0192
5/21/2006	-0.0232
5/22/2006	-0.0197
5/23/2006	-0.0159
5/24/2006	-0.0161
5/25/2006	-0.0145
5/26/2006	-0.0165
5/27/2006	-0.0115
5/28/2006	-0.0157
5/29/2006	-0.0096
5/30/2006	-0.0118
5/31/2006	-0.0177
6/1/2006	-0.0124
6/2/2006	-0.0130
6/3/2006	-0.0156
6/4/2006	-0.0144
6/5/2006	-0.0213
6/6/2006	-0.0211
6/7/2006	-0.0247
6/8/2006	-0.0110
6/9/2006	-0.0170
6/10/2006	-0.0170
6/11/2006	-0.0209
6/12/2006	-0.0119
6/13/2006	-0.0142
6/14/2006	-0.0129
6/15/2006	-0.0182
6/16/2006	-0.0118
6/18/2006	-0.0188
6/20/2006	-0.0179
6/22/2006	-0.0163
6/23/2006	-0.0224
6/24/2006	-0.0160
6/25/2006	-0.0139
6/26/2006	-0.0190
6/27/2006	-0.0145
6/28/2006	-0.0157
6/29/2006	-0.0184
6/30/2006	-0.0205
7/2/2006	-0.0217
7/3/2006	-0.0171
7/4/2006	-0.0214

7/5/2006	-0.0138
7/6/2006	-0.0175
7/7/2006	-0.0108
7/8/2006	-0.0250
7/9/2006	-0.0194
7/12/2006	-0.0200
7/13/2006	-0.0179
7/14/2006	-0.0229
7/15/2006	-0.0217
7/16/2006	-0.0225
7/17/2006	-0.0207
7/18/2006	-0.0232
7/19/2006	-0.0204
7/20/2006	-0.0174
7/21/2006	-0.0219
7/22/2006	-0.0193
7/23/2006	-0.0168
7/24/2006	-0.0199
7/25/2006	-0.0162
7/26/2006	-0.0199
7/27/2006	-0.0160
7/28/2006	-0.0178
7/29/2006	-0.0222
7/30/2006	-0.0128
7/31/2006	-0.0148
8/1/2006	-0.0178
8/2/2006	-0.0158
8/3/2006	-0.0129
8/4/2006	-0.0176
8/5/2006	-0.0150
8/6/2006	-0.0170
8/7/2006	-0.0108
8/8/2006	-0.0182
8/9/2006	-0.0219
8/10/2006	-0.0139
8/11/2006	-0.0095
8/12/2006	-0.0115
8/13/2006	-0.0188
8/14/2006	-0.0149
8/15/2006	-0.0129
8/16/2006	-0.0143
8/17/2006	-0.0131
8/18/2006	-0.0124
8/19/2006	-0.0102
8/20/2006	-0.0137
8/21/2006	-0.0020
8/22/2006	-0.0063
8/23/2006	-0.0088
8/24/2006	-0.0057
8/25/2006	-0.0093

8/26/2006	-0.0117
8/27/2006	-0.0051
8/28/2006	-0.0050
8/29/2006	-0.0142
8/30/2006	-0.0058
8/31/2006	-0.0116
9/1/2006	-0.0075
9/2/2006	-0.0069
9/3/2006	-0.0043
9/4/2006	-0.0087
9/5/2006	-0.0025
9/6/2006	-0.0062
9/7/2006	-0.0063
9/8/2006	-0.0022
9/9/2006	-0.0014
9/10/2006	-0.0068
9/11/2006	-0.0002
9/12/2006	-0.0081
9/13/2006	-0.0053
9/14/2006	0.0004
9/16/2006	-0.0105
9/17/2006	-0.0011
9/18/2006	-0.0116
9/19/2006	0.0005
9/20/2006	-0.0085
9/21/2006	-0.0059
9/22/2006	-0.0025
9/23/2006	-0.0068
9/24/2006	-0.0067
9/25/2006	-0.0041
9/26/2006	-0.0011
9/27/2006	-0.0046
9/28/2006	-0.0117
9/29/2006	-0.0130
9/30/2006	-0.0096
10/1/2006	-0.0037
10/2/2006	-0.0048
10/3/2006	-0.0027
10/4/2006	0.0025
10/5/2006	-0.0084
10/6/2006	-0.0097
10/7/2006	-0.0082
10/8/2006	-0.0073
10/9/2006	-0.0036
10/10/2006	-0.0061
10/11/2006	-0.0038
10/12/2006	-0.0054
10/13/2006	-0.0050
10/14/2006	-0.0030
10/15/2006	-0.0028

10/16/2006	-0.0059
10/17/2006	-0.0047
10/18/2006	-0.0061
10/19/2006	-0.0071
10/20/2006	-0.0047
10/21/2006	0.0049
10/22/2006	-0.0051
10/23/2006	-0.0028
10/24/2006	-0.0045
10/25/2006	0.0014
10/26/2006	0.0007
10/27/2006	0.0035
10/28/2006	0.0046
10/29/2006	0.0092
10/30/2006	0.0060
10/31/2006	0.0043
11/1/2006	-0.0036
11/2/2006	0.0030
11/3/2006	0.0068
11/4/2006	0.0020
11/5/2006	0.0025
11/6/2006	-0.0010
11/7/2006	0.0018
11/8/2006	-0.0006
11/9/2006	0.0097
11/10/2006	0.0009
11/11/2006	0.0031
11/13/2006	0.0039
11/14/2006	0.0024
11/15/2006	0.0001
11/16/2006	-0.0027
11/17/2006	0.0002
11/18/2006	0.0038
11/19/2006	0.0013
11/20/2006	0.0003
11/21/2006	0.0029
11/22/2006	-0.0058
11/23/2006	-0.0017
11/24/2006	-0.0020
11/25/2006	-0.0019
11/26/2006	0.0002
11/27/2006	0.0009
11/28/2006	-0.0006
11/29/2006	0.0017
11/30/2006	0.0044
12/1/2006	0.0049
12/2/2006	0.0043
12/3/2006	-0.0005
12/4/2006	0.0062
12/5/2006	0.0043

12/6/2006	0.0054
12/7/2006	0.0010
12/8/2006	0.0002
12/9/2006	0.0004
12/10/2006	-0.0016
12/11/2006	0.0036
12/12/2006	0.0068
12/13/2006	0.0050
12/14/2006	0.0032
12/15/2006	0.0061
12/16/2006	0.0062
12/17/2006	0.0115
12/18/2006	0.0126
12/19/2006	0.0043
12/20/2006	0.0054
12/21/2006	0.0099
12/22/2006	0.0063
12/23/2006	0.0054
12/24/2006	0.0074
12/25/2006	0.0082
12/26/2006	0.0122
12/27/2006	0.0065
12/28/2006	0.0088
12/29/2006	0.0072
12/30/2006	0.0062
12/31/2006	0.0102
1/1/2007	0.0082
1/2/2007	0.0127
1/3/2007	0.0093
1/4/2007	0.0063
1/5/2007	0.0064
1/6/2007	0.0027
1/7/2007	0.0065
1/8/2007	0.0017
1/9/2007	-0.0037
1/10/2007	0.0033
1/11/2007	0.0021
1/12/2007	0.0088
1/13/2007	0.0041
1/14/2007	0.0054
1/15/2007	0.0072
1/16/2007	0.0025
1/17/2007	0.0011
1/18/2007	0.0049
1/19/2007	0.0024
1/20/2007	0.0061
1/22/2007	0.0048
1/23/2007	0.0027
1/24/2007	0.0067
1/25/2007	0.0053

1/26/2007	0.0100
1/27/2007	0.0083
1/28/2007	0.0036
1/29/2007	0.0040
1/30/2007	0.0044
1/31/2007	0.0040
2/1/2007	0.0042
2/2/2007	0.0073
2/3/2007	0.0050
2/4/2007	0.0006
2/5/2007	0.0045
2/6/2007	-0.0014
2/7/2007	-0.0032
2/8/2007	-0.0069
2/9/2007	0.0029
2/10/2007	0.0015
2/11/2007	-0.0029
2/12/2007	0.0022
2/13/2007	0.0034
2/14/2007	0.0002
2/15/2007	-0.0009
2/16/2007	0.0008
2/17/2007	0.0000
2/18/2007	0.0015
2/19/2007	-0.0017
2/20/2007	-0.0009
2/21/2007	-0.0008
2/22/2007	-0.0048
2/23/2007	0.0042
2/24/2007	-0.0037
2/25/2007	-0.0033
2/26/2007	-0.0015
2/28/2007	-0.0012
3/1/2007	-0.0038
3/2/2007	0.0005
3/3/2007	-0.0008
3/4/2007	-0.0017
3/5/2007	-0.0015
3/6/2007	-0.0050
3/7/2007	-0.0048
3/8/2007	0.0026
3/9/2007	0.0005
3/10/2007	0.0034
3/11/2007	-0.0013
3/12/2007	-0.0039
3/13/2007	0.0002
3/14/2007	-0.0031
3/15/2007	-0.0017
3/16/2007	-0.0067
3/17/2007	-0.0032

3/18/2007	-0.0031
3/19/2007	-0.0005
3/20/2007	-0.0111
3/21/2007	-0.0035
3/22/2007	0.0011
3/23/2007	0.0001
3/24/2007	-0.0039
3/25/2007	-0.0007
3/26/2007	-0.0050
3/27/2007	-0.0054
3/28/2007	-0.0035
3/29/2007	-0.0012
3/30/2007	0.0040
3/31/2007	-0.0543
4/1/2007	-0.0060
4/2/2007	-0.0096
4/3/2007	-0.0017
4/4/2007	-0.0024
4/5/2007	0.0046
4/6/2007	0.0053
4/10/2007	0.0198
4/11/2007	-0.0089
4/12/2007	-0.0015
4/13/2007	0.0002
4/14/2007	-0.0034
4/15/2007	-0.0042
4/16/2007	-0.0067
4/17/2007	-0.0081
4/18/2007	-0.0039
4/27/2007	0.0012
4/28/2007	0.0021
4/29/2007	-0.0091
4/30/2007	-0.0070
5/1/2007	-0.0061
5/2/2007	0.0018
5/3/2007	-0.0011
5/4/2007	-0.0030
5/5/2007	-0.0024
5/6/2007	-0.0031
5/7/2007	-0.0086
5/8/2007	-0.0065
5/9/2007	-0.0077
5/10/2007	-0.0083
5/11/2007	-0.0061
5/12/2007	-0.0059
5/13/2007	-0.0067
5/14/2007	-0.0060
5/15/2007	-0.0037
5/16/2007	0.0036
5/17/2007	-0.0039

5/18/2007	-0.0064
5/19/2007	-0.0040
5/20/2007	-0.0110
5/21/2007	0.0001
5/22/2007	-0.0025
5/24/2007	-0.0005
5/25/2007	-0.0081
5/26/2007	-0.0117
5/27/2007	-0.0038
5/28/2007	-0.0151
5/29/2007	-0.0105
5/30/2007	-0.0087
5/31/2007	-0.0116
6/1/2007	-0.0102
6/2/2007	-0.0103
6/3/2007	-0.0057
6/4/2007	0.0014
6/5/2007	-0.0114
6/6/2007	-0.0101
6/7/2007	-0.0087
6/8/2007	-0.0111
6/9/2007	-0.0120
6/10/2007	-0.0107
6/11/2007	-0.0090
6/12/2007	-0.0083
6/13/2007	-0.0167
6/14/2007	-0.0137
6/15/2007	-0.0041
6/16/2007	-0.0144
6/17/2007	-0.0087
6/18/2007	-0.0130
6/19/2007	-0.0095
6/20/2007	-0.0111
6/21/2007	-0.0100
6/22/2007	-0.0139
6/23/2007	-0.0125
6/24/2007	-0.0125
6/25/2007	-0.0066
6/26/2007	-0.0056
6/27/2007	-0.0108
6/28/2007	-0.0134
6/29/2007	-0.0138
6/30/2007	-0.0132
7/1/2007	-0.0142
7/2/2007	-0.0149
7/3/2007	-0.0126
7/4/2007	-0.0116
7/5/2007	-0.0138
7/6/2007	-0.0128
7/7/2007	-0.0161

7/8/2007	-0.0161
7/9/2007	-0.0164
7/10/2007	-0.0102
7/11/2007	-0.0083
7/12/2007	-0.0029
7/13/2007	-0.0120
7/14/2007	-0.0111
7/15/2007	-0.0150
7/16/2007	-0.0204
7/17/2007	-0.0140
7/18/2007	-0.0096
7/19/2007	-0.0118
7/20/2007	-0.0204
7/21/2007	-0.0157
7/22/2007	-0.0122
7/23/2007	-0.0161
7/24/2007	-0.0141
7/25/2007	-0.0083
7/26/2007	-0.0150
7/27/2007	-0.0131
7/28/2007	-0.0115
7/29/2007	-0.0102
7/30/2007	-0.0112
7/31/2007	-0.0175
8/1/2007	-0.0158
8/2/2007	-0.0113
8/3/2007	-0.0188
8/5/2007	-0.0141
8/6/2007	-0.0115
8/7/2007	-0.0162
8/8/2007	-0.0203
8/9/2007	-0.0207
8/10/2007	-0.0079
8/11/2007	-0.0204
8/12/2007	-0.0113
8/13/2007	-0.0184
8/14/2007	-0.0131
8/15/2007	-0.0182
8/16/2007	-0.0148
8/17/2007	-0.0135
8/18/2007	-0.0122
8/19/2007	-0.0182
8/20/2007	-0.0132
8/21/2007	-0.0116
8/22/2007	-0.0062
8/23/2007	-0.0106
8/24/2007	-0.0084
8/25/2007	-0.0099
8/26/2007	-0.0134
8/27/2007	-0.0097

8/28/2007	-0.0138
8/29/2007	0.0013
8/30/2007	-0.0175
8/31/2007	-0.0072
9/1/2007	-0.0145
9/2/2007	-0.0169
9/3/2007	-0.0155
9/4/2007	-0.0100
9/5/2007	-0.0177
9/6/2007	-0.0172
9/7/2007	-0.0153
9/8/2007	-0.0151
9/9/2007	-0.0139
9/10/2007	-0.0246
9/11/2007	-0.0098
9/12/2007	-0.0117
9/13/2007	-0.0205
9/14/2007	-0.0145
9/15/2007	-0.0216
9/16/2007	-0.0178
9/17/2007	-0.0200
9/18/2007	-0.0152
9/19/2007	-0.0089
9/20/2007	-0.0153
9/21/2007	-0.0106
9/22/2007	-0.0179
9/23/2007	-0.0171
9/24/2007	-0.0153
9/25/2007	-0.0165
9/26/2007	-0.0187
9/27/2007	-0.0116
9/28/2007	-0.0195
9/29/2007	-0.0191
9/30/2007	-0.0195
10/1/2007	-0.0180
10/2/2007	-0.0158
10/3/2007	-0.0185
10/4/2007	-0.0174
10/5/2007	-0.0138
10/6/2007	-0.0254
10/7/2007	-0.0188
10/8/2007	-0.0203
10/9/2007	-0.0184
10/10/2007	-0.0222
10/11/2007	-0.0160
10/12/2007	-0.0152
10/13/2007	-0.0166
10/14/2007	-0.0185
10/15/2007	-0.0192
10/16/2007	-0.0193

10/17/2007	-0.0140
10/18/2007	-0.0150
10/19/2007	-0.0205
10/20/2007	-0.0177
10/21/2007	-0.0112
10/22/2007	-0.0152
10/23/2007	-0.0190
10/24/2007	-0.0170
10/25/2007	-0.0136
10/26/2007	-0.0164
10/27/2007	-0.0167
10/28/2007	-0.0148
10/29/2007	-0.0109
10/30/2007	-0.0144
10/31/2007	-0.0185
11/1/2007	-0.0179
11/2/2007	-0.0127
11/5/2007	-0.0105
11/6/2007	-0.0154
11/8/2007	-0.0150
11/9/2007	-0.0169
11/10/2007	-0.0183
11/11/2007	-0.0131
11/12/2007	-0.0154
11/13/2007	-0.0153
11/14/2007	-0.0091
11/15/2007	-0.0111
11/16/2007	-0.0124
11/17/2007	-0.0160
11/18/2007	-0.0177
11/19/2007	-0.0202
11/20/2007	-0.0194
11/22/2007	-0.0181
11/23/2007	-0.0155
11/24/2007	-0.0120
11/25/2007	-0.0152
11/26/2007	-0.0099
11/27/2007	-0.0135
11/28/2007	-0.0108
11/29/2007	-0.0098
11/30/2007	-0.0127
12/1/2007	-0.0139
12/2/2007	-0.0125
12/3/2007	-0.0097
12/4/2007	-0.0178
12/5/2007	-0.0163
12/6/2007	-0.0135
12/7/2007	-0.0106
12/8/2007	-0.0156
12/9/2007	-0.0090

12/10/2007	-0.0033
12/11/2007	-0.0089
12/12/2007	-0.0044
12/13/2007	-0.0071
12/14/2007	-0.0109
12/15/2007	-0.0075
12/16/2007	-0.0062
12/17/2007	-0.0054
12/18/2007	-0.0065
12/19/2007	-0.0117
12/21/2007	-0.0070
12/22/2007	-0.0114
12/23/2007	-0.0093
12/24/2007	-0.0106
12/25/2007	-0.0103
12/26/2007	-0.0090
12/28/2007	-0.0126
12/29/2007	-0.0113
12/30/2007	-0.0088
12/31/2007	-0.0074
1/1/2008	-0.0058
1/2/2008	-0.0084
1/3/2008	-0.0072
1/4/2008	-0.0124
1/5/2008	-0.0080
1/6/2008	-0.0078
1/7/2008	-0.0079
1/8/2008	-0.0047
1/9/2008	-0.0141
1/10/2008	-0.0068
1/11/2008	-0.0125
1/12/2008	-0.0069
1/13/2008	-0.0031
1/14/2008	-0.0099
1/15/2008	-0.0286
1/16/2008	-0.0029
1/17/2008	-0.0125
1/18/2008	-0.0091
1/19/2008	-0.0101
1/20/2008	-0.0086
1/21/2008	-0.0103
1/22/2008	-0.0106
1/23/2008	-0.0129
1/24/2008	-0.0183
1/25/2008	-0.0073
1/26/2008	-0.0084
1/27/2008	-0.0079
1/29/2008	-0.0117
1/30/2008	-0.0113
1/31/2008	-0.0130

2/1/2008	-0.0154
2/2/2008	-0.0081
2/3/2008	-0.0115
2/4/2008	-0.0092
2/5/2008	-0.0128
2/6/2008	-0.0087
2/7/2008	-0.0107
2/8/2008	-0.0060
2/9/2008	-0.0067
2/10/2008	-0.0098
2/11/2008	-0.0097
2/12/2008	-0.0105
2/13/2008	-0.0101
2/14/2008	-0.0084
2/15/2008	-0.0085
2/16/2008	-0.0058
2/17/2008	-0.0085
2/18/2008	-0.0091
2/19/2008	-0.0128
2/20/2008	-0.0096
2/21/2008	-0.0110
2/22/2008	-0.0110
2/23/2008	-0.0092
2/24/2008	-0.0139
2/25/2008	-0.0127
2/26/2008	-0.0175
2/27/2008	-0.0112
2/28/2008	-0.0123
2/29/2008	-0.0120
3/1/2008	-0.0118
3/2/2008	-0.0074
3/3/2008	-0.0106
3/4/2008	-0.0091
3/5/2008	-0.0061
3/6/2008	-0.0113
3/7/2008	-0.0097
3/8/2008	-0.0170
3/9/2008	-0.0117
3/10/2008	-0.0185
3/11/2008	-0.0110
3/12/2008	-0.0112
3/13/2008	-0.0068
3/14/2008	-0.0111
3/15/2008	-0.0104
3/16/2008	-0.0095
3/17/2008	-0.0126
3/18/2008	-0.0089
3/19/2008	-0.0103
3/20/2008	-0.0213
3/21/2008	-0.0106

3/22/2008	-0.0087
3/23/2008	-0.0098
3/24/2008	-0.0076
3/25/2008	-0.0130
3/26/2008	-0.0129
3/27/2008	-0.0171
3/28/2008	-0.0119
3/29/2008	-0.0120
3/30/2008	-0.0216
3/31/2008	-0.0085
4/1/2008	-0.0051
4/2/2008	-0.0096
4/3/2008	-0.0098
4/4/2008	-0.0132
4/5/2008	-0.0096
4/6/2008	-0.0138
4/7/2008	-0.0092
4/8/2008	-0.0137
4/9/2008	-0.0120
4/10/2008	-0.0122
4/11/2008	-0.0137
4/12/2008	-0.0137
4/13/2008	-0.0133
4/14/2008	-0.0072
4/15/2008	-0.0143
4/16/2008	-0.0062
4/17/2008	-0.0088
4/18/2008	-0.0100
4/19/2008	-0.0121
4/20/2008	-0.0074
4/21/2008	-0.0172
4/22/2008	-0.0151
4/23/2008	-0.0117
4/24/2008	-0.0107
4/25/2008	-0.0048
4/26/2008	-0.0055
4/27/2008	-0.0073
4/28/2008	-0.0081
4/29/2008	-0.0118
4/30/2008	-0.0106
5/1/2008	-0.0113
5/2/2008	-0.0062
5/3/2008	-0.0065
5/4/2008	-0.0053
5/5/2008	-0.0087
5/6/2008	-0.0097
5/7/2008	-0.0158
5/8/2008	-0.0059
5/9/2008	-0.0062
5/10/2008	-0.0101

5/11/2008	-0.0026
5/12/2008	-0.0129
5/13/2008	-0.0147
5/14/2008	-0.0076
5/15/2008	-0.0107
5/16/2008	-0.0096
5/17/2008	-0.0074
5/18/2008	-0.0068
5/19/2008	-0.0021
5/20/2008	-0.0074
5/21/2008	-0.0029
5/22/2008	-0.0128
5/23/2008	-0.0090
5/24/2008	-0.0117
5/25/2008	-0.0113
5/26/2008	-0.0124
5/27/2008	-0.0085
5/28/2008	-0.0070
5/29/2008	-0.0102
5/30/2008	-0.0140
5/31/2008	-0.0089
6/1/2008	-0.0127
6/2/2008	-0.0072
6/3/2008	-0.0118
6/4/2008	-0.0182
6/5/2008	-0.0082
6/6/2008	-0.0014
6/7/2008	-0.0085
6/8/2008	-0.0121
6/9/2008	-0.0131
6/10/2008	0.0020
6/11/2008	-0.0097
6/12/2008	-0.0131
6/13/2008	-0.0061
6/14/2008	-0.0096
6/15/2008	-0.0134
6/16/2008	-0.0051
6/17/2008	-0.0098
6/18/2008	-0.0119
6/19/2008	-0.0126
6/20/2008	-0.0020
6/21/2008	-0.0159
6/22/2008	-0.0137
6/23/2008	-0.0176
6/24/2008	0.0075
6/25/2008	-0.0148
6/26/2008	-0.0043
6/27/2008	-0.0081
6/28/2008	-0.0019
6/29/2008	-0.0095

6/30/2008	-0.0104
7/1/2008	-0.0092
7/2/2008	-0.0106
7/3/2008	-0.0139
7/4/2008	-0.0073
7/5/2008	-0.0142
7/6/2008	-0.0122
7/7/2008	-0.0104
7/8/2008	-0.0151
7/9/2008	-0.0102
7/10/2008	-0.0104
7/11/2008	-0.0101
7/12/2008	-0.0144
7/13/2008	-0.0119
7/14/2008	-0.0043
7/15/2008	-0.0071
7/16/2008	-0.0070
7/17/2008	-0.0089
7/18/2008	-0.0052
7/19/2008	-0.0052
7/20/2008	-0.0144
7/21/2008	-0.0072
7/22/2008	-0.0056
7/23/2008	-0.0100
7/24/2008	-0.0054
7/25/2008	-0.0088
7/26/2008	-0.0108
7/27/2008	-0.0119
7/28/2008	-0.0050
7/29/2008	-0.0150
7/30/2008	-0.0037
7/31/2008	-0.0081
8/1/2008	-0.0009
8/2/2008	-0.0113
8/3/2008	-0.0058
8/4/2008	-0.0064
8/5/2008	-0.0105
8/6/2008	-0.0098
8/7/2008	-0.0071
8/8/2008	-0.0112
8/9/2008	-0.0070
8/10/2008	-0.0059
8/11/2008	-0.0054
8/12/2008	-0.0106
8/13/2008	-0.0069
8/14/2008	-0.0099
8/15/2008	0.0040
8/16/2008	-0.0043
8/17/2008	-0.0152
8/18/2008	-0.0079

8/19/2008	-0.0051
8/20/2008	-0.0057
8/21/2008	-0.0038
8/22/2008	0.0002
8/23/2008	-0.0044
8/24/2008	-0.0058
8/25/2008	-0.0076
8/26/2008	-0.0025
8/27/2008	-0.0029
8/28/2008	-0.0044
8/29/2008	-0.0054
8/30/2008	-0.0094
8/31/2008	-0.0091
9/1/2008	-0.0045
9/2/2008	-0.0060
9/3/2008	-0.0105
9/4/2008	-0.0066
9/5/2008	-0.0114
9/6/2008	-0.0119
9/7/2008	-0.0083
9/8/2008	-0.0092
9/9/2008	-0.0108
9/10/2008	-0.0138
9/11/2008	-0.0040
9/12/2008	-0.0120
9/13/2008	0.0074
9/14/2008	-0.0069
9/15/2008	-0.0037
9/16/2008	-0.0088
9/17/2008	-0.0034
9/18/2008	-0.0035
9/19/2008	-0.0061
9/20/2008	-0.0003
9/21/2008	-0.0084
9/22/2008	-0.0110
9/23/2008	-0.0093
9/24/2008	-0.0110
9/25/2008	-0.0043
9/26/2008	-0.0055
9/27/2008	-0.0071
9/28/2008	-0.0081
9/29/2008	-0.0063
9/30/2008	-0.0079
10/1/2008	-0.0058
10/2/2008	0.0084
10/3/2008	-0.0041
10/4/2008	-0.0035
10/5/2008	-0.0097
10/6/2008	-0.0047
10/7/2008	-0.0054

10/8/2008	-0.0064
10/9/2008	-0.0047
10/10/2008	-0.0073
10/11/2008	-0.0061
10/12/2008	-0.0027
10/13/2008	-0.0031
10/14/2008	-0.0058
10/15/2008	-0.0065
10/16/2008	-0.0054
10/17/2008	-0.0005
10/18/2008	-0.0017
10/19/2008	-0.0082
10/20/2008	-0.0018
10/21/2008	-0.0041
10/22/2008	-0.0058
10/23/2008	-0.0027
10/24/2008	-0.0022
10/25/2008	-0.0041
10/26/2008	-0.0015
10/27/2008	-0.0034
10/28/2008	0.0092
10/29/2008	-0.0116
10/30/2008	-0.0057
10/31/2008	-0.0026
11/1/2008	-0.0087
11/2/2008	-0.0124
11/3/2008	0.0025
11/4/2008	0.0029
11/5/2008	-0.0032
11/6/2008	-0.0034
11/7/2008	-0.0046
11/8/2008	-0.0067
11/9/2008	-0.0053
11/10/2008	-0.0063
11/11/2008	-0.0027
11/12/2008	-0.0045
11/13/2008	0.0017
11/14/2008	0.0012
11/15/2008	0.0024
11/16/2008	-0.0026
11/17/2008	0.0099
11/18/2008	-0.0063
11/19/2008	0.0010
11/20/2008	0.0009
11/21/2008	0.0000
11/22/2008	0.0026
11/23/2008	0.0022
11/24/2008	0.0001
11/25/2008	0.0021
11/26/2008	0.0014

11/27/2008	0.0001
11/28/2008	0.0049
11/29/2008	-0.0069
11/30/2008	-0.0012
12/1/2008	-0.0059
12/2/2008	-0.0040
12/3/2008	-0.0035
12/4/2008	-0.0010
12/5/2008	-0.0026
12/6/2008	0.0032
12/7/2008	-0.0006
12/8/2008	0.0026
12/9/2008	-0.0018
12/10/2008	-0.0002
12/11/2008	-0.0072
12/12/2008	0.0046
12/13/2008	-0.0024
12/14/2008	-0.0117
12/15/2008	-0.0064
12/16/2008	0.0091
12/17/2008	0.0032
12/18/2008	0.0000
12/19/2008	-0.0015
12/20/2008	-0.0017
12/21/2008	-0.0006
12/22/2008	-0.0015
12/23/2008	0.0018
12/24/2008	-0.0018
12/25/2008	-0.0018
12/26/2008	0.0033
12/27/2008	-0.0022
12/28/2008	0.0010
12/29/2008	-0.0018
12/30/2008	-0.0019
12/31/2008	0.0019
1/1/2009	0.0058
1/2/2009	0.0026
1/3/2009	0.0014
1/4/2009	0.0015
1/5/2009	0.0024
1/6/2009	0.0033
1/7/2009	0.0068
1/8/2009	0.0043
1/9/2009	0.0055
1/10/2009	-0.0011
1/11/2009	0.0031
1/12/2009	-0.0008
1/13/2009	0.0010
1/14/2009	0.0086
1/15/2009	-0.0023

1/16/2009	-0.0027
1/17/2009	0.0000
1/18/2009	0.0048
1/19/2009	-0.0217
1/20/2009	0.0014
1/21/2009	0.0058
1/22/2009	0.0043
1/23/2009	-0.0020
1/24/2009	-0.0089
1/25/2009	-0.0080
1/26/2009	0.0007
1/27/2009	-0.0111
1/28/2009	-0.0095
1/29/2009	-0.0029
1/30/2009	-0.0051
1/31/2009	-0.0046
2/1/2009	-0.0047
2/2/2009	-0.0109
2/3/2009	-0.0061
2/4/2009	-0.0077
2/5/2009	-0.0047
2/6/2009	-0.0064
2/7/2009	-0.0019
2/8/2009	-0.0045
2/9/2009	-0.0037
2/10/2009	-0.0025
2/11/2009	-0.0027
2/12/2009	-0.0010
2/13/2009	-0.0008
2/14/2009	0.0002
2/15/2009	-0.0018
2/16/2009	-0.0006
2/17/2009	-0.0005
2/18/2009	0.0018
2/19/2009	0.0004
2/20/2009	-0.0012
2/21/2009	0.0066
2/22/2009	0.0027
2/23/2009	-0.0020
2/24/2009	-0.0014
2/25/2009	0.0037
2/26/2009	0.0023
2/27/2009	0.0024
2/28/2009	0.0005
3/1/2009	-0.0015
3/2/2009	-0.0005
3/3/2009	-0.0008
3/4/2009	-0.0060
3/5/2009	0.0034
3/6/2009	0.0043

3/7/2009	0.0020
3/8/2009	-0.0033
3/9/2009	-0.0043
3/10/2009	-0.0047
3/11/2009	-0.0007
3/12/2009	0.0080
3/13/2009	0.0023
3/14/2009	0.0006
3/15/2009	0.0045
3/16/2009	0.0027
3/17/2009	0.0061
3/18/2009	0.0005
3/19/2009	0.0033
3/20/2009	0.0031
3/21/2009	0.0055
3/22/2009	0.0013
3/23/2009	0.0019
3/24/2009	0.0113
3/25/2009	0.0024
3/26/2009	0.0030
3/27/2009	0.0023
3/28/2009	0.0026
3/29/2009	0.0079
3/30/2009	0.0072
3/31/2009	0.0006
4/1/2009	0.0029
4/2/2009	0.0021
4/3/2009	0.0038
4/4/2009	0.0032
4/5/2009	0.0039
4/6/2009	0.0018
4/7/2009	0.0047
4/8/2009	0.0056
4/9/2009	0.0007
4/10/2009	0.0027
4/11/2009	0.0014
4/12/2009	0.0023
4/13/2009	0.0064
4/14/2009	0.0035
4/15/2009	0.0019
4/16/2009	0.0028
4/18/2009	0.0034
4/19/2009	0.0033
4/20/2009	0.0011
4/21/2009	0.0028
4/22/2009	0.0057
4/23/2009	0.0093
4/24/2009	0.0014
4/25/2009	-0.0010
4/26/2009	0.0012

4/27/2009	0.0034
4/28/2009	-0.0034
4/29/2009	0.0033
4/30/2009	0.0001
5/1/2009	0.0047
5/2/2009	0.0064
5/3/2009	-0.0004
5/4/2009	0.0041
5/5/2009	0.0090
5/6/2009	0.0036
5/7/2009	0.0018
5/8/2009	0.0030
5/9/2009	0.0041
5/10/2009	0.0040
5/11/2009	0.0025
5/12/2009	0.0045
5/13/2009	0.0033
5/14/2009	-0.0002
5/15/2009	0.0069
5/16/2009	0.0068
5/17/2009	0.0056
5/18/2009	0.0066
5/19/2009	0.0016
5/20/2009	0.0074
5/21/2009	0.0038
5/22/2009	0.0074
5/23/2009	0.0014
5/24/2009	0.0005
5/25/2009	0.0050
5/26/2009	0.0036
5/27/2009	0.0109
5/28/2009	0.0097
5/29/2009	0.0026
5/30/2009	0.0076
5/31/2009	0.0075
6/1/2009	0.0082
6/2/2009	0.0027
6/3/2009	0.0044
6/4/2009	0.0022
6/5/2009	0.0070
6/6/2009	0.0053
6/7/2009	0.0060
6/8/2009	0.0045
6/9/2009	0.0021
6/10/2009	0.0076
6/11/2009	0.0013
6/12/2009	0.0083
6/14/2009	0.0046
6/15/2009	0.0046
6/16/2009	0.0081

6/17/2009	0.0126
6/18/2009	0.0103
6/19/2009	0.0068
6/20/2009	0.0163
6/21/2009	0.0085
6/22/2009	0.0195
6/23/2009	0.0091
6/24/2009	0.0049
6/25/2009	0.0083
6/26/2009	0.0019
6/27/2009	0.0018
6/28/2009	0.0068
6/29/2009	0.0013
6/30/2009	0.0050
7/1/2009	0.0074
7/2/2009	0.0128
7/3/2009	0.0057
7/4/2009	0.0119
7/5/2009	0.0078
7/6/2009	0.0078
7/7/2009	0.0149
7/8/2009	0.0078
7/9/2009	0.0091
7/10/2009	0.0124
7/11/2009	0.0115
7/12/2009	0.0072
7/13/2009	0.0080
7/14/2009	0.0089
7/15/2009	0.0088
7/16/2009	0.0140
7/17/2009	0.0072
7/18/2009	0.0088
7/19/2009	0.0124
7/20/2009	0.0073
7/21/2009	0.0124
7/22/2009	0.0100
7/23/2009	0.0075
7/24/2009	0.0123
7/25/2009	0.0185
7/26/2009	0.0088
7/27/2009	0.0058
7/28/2009	0.0089
7/29/2009	0.0123
7/30/2009	0.0144
7/31/2009	0.0110
8/1/2009	0.0086
8/2/2009	0.0111
8/3/2009	0.0104
8/4/2009	0.0096
8/5/2009	0.0070

8/6/2009	0.0099
8/7/2009	0.0080
8/8/2009	0.0071
8/9/2009	0.0052
8/10/2009	0.0084
8/11/2009	0.0017
8/12/2009	0.0061
8/13/2009	0.0047
8/14/2009	0.0077
8/15/2009	0.0074
8/16/2009	0.0094
8/17/2009	0.0116
8/18/2009	0.0099
8/19/2009	0.0066
8/20/2009	0.0109
8/21/2009	0.0086
8/22/2009	0.0079
8/23/2009	0.0038
8/24/2009	0.0082
8/25/2009	0.0089
8/26/2009	0.0078
8/27/2009	0.0188
8/28/2009	0.0132
8/29/2009	0.0056
8/30/2009	0.0060
8/31/2009	0.0045
9/1/2009	0.0048
9/2/2009	0.0146
9/3/2009	0.0099
9/4/2009	0.0001
9/5/2009	0.0093
9/6/2009	0.0210
9/7/2009	0.0166
9/8/2009	0.0175
9/9/2009	0.0090
9/10/2009	0.0124
9/11/2009	0.0107
9/12/2009	0.0123
9/13/2009	0.0094
9/14/2009	0.0135
9/15/2009	0.0122
9/16/2009	0.0086
9/17/2009	0.0105
9/18/2009	0.0072
9/19/2009	0.0136
9/20/2009	0.0050
9/21/2009	0.0097
9/22/2009	0.0077
9/23/2009	0.0061
9/24/2009	0.0123

9/25/2009	0.0195
9/26/2009	0.0107
9/27/2009	0.0069
9/28/2009	0.0039
9/29/2009	0.0114
9/30/2009	0.0065
10/1/2009	0.0101
10/2/2009	0.0099
10/3/2009	0.0093
10/4/2009	0.0066
10/5/2009	0.0092
10/6/2009	0.0043
10/7/2009	0.0101
10/8/2009	0.0101
10/9/2009	0.0115
10/10/2009	0.0062
10/11/2009	0.0088
10/12/2009	0.0191
10/13/2009	0.0107
10/14/2009	0.0103
10/15/2009	0.0028
10/16/2009	0.0088
10/17/2009	0.0071
10/18/2009	0.0090
10/19/2009	0.0083
10/20/2009	0.0013
10/21/2009	0.0031
10/22/2009	0.0020
10/23/2009	0.0117
10/24/2009	0.0132
10/25/2009	0.0060
10/26/2009	0.0058
10/27/2009	0.0020
10/28/2009	0.0051
10/29/2009	0.0040
10/30/2009	0.0054
10/31/2009	0.0035
11/1/2009	0.0032
11/2/2009	0.0019
11/3/2009	0.0112
11/4/2009	0.0048
11/5/2009	0.0004
11/6/2009	0.0073
11/7/2009	0.0017
11/8/2009	0.0084
11/9/2009	0.0060
11/10/2009	0.0143
11/11/2009	0.0056
11/12/2009	0.0062
11/13/2009	0.0074

11/14/2009	0.0036
11/15/2009	0.0066
11/16/2009	0.0068
11/17/2009	0.0057
11/18/2009	0.0084
11/19/2009	0.0080
11/20/2009	0.0079
11/21/2009	0.0089
11/22/2009	0.0034
11/23/2009	0.0087
11/25/2009	0.0055
11/26/2009	0.0097
11/27/2009	0.0098
11/28/2009	0.0083
11/29/2009	0.0065
11/30/2009	-0.0085
12/1/2009	0.0035
12/2/2009	0.0037
12/3/2009	0.0055
12/4/2009	0.0043
12/5/2009	0.0071
12/6/2009	0.0018
12/7/2009	0.0018
12/8/2009	0.0030
12/9/2009	0.0055
12/10/2009	0.0096
12/11/2009	0.0115
12/12/2009	0.0103
12/13/2009	0.0094
12/14/2009	0.0101
12/15/2009	0.0119
12/16/2009	0.0061
12/17/2009	0.0081
12/18/2009	0.0056
12/19/2009	0.0105
12/20/2009	0.0087
12/21/2009	0.0051
12/22/2009	-0.0054
12/23/2009	0.0054
12/24/2009	0.0045
12/25/2009	0.0011
12/26/2009	0.0022
12/27/2009	-0.0002
12/28/2009	0.0030
12/29/2009	0.0029
12/30/2009	0.0021
12/31/2009	0.0021
1/1/2010	0.0066
1/2/2010	0.0090
1/3/2010	0.0067

1/4/2010	0.0055
1/5/2010	0.0034
1/6/2010	0.0068
1/7/2010	0.0040
1/8/2010	0.0105
1/9/2010	0.0168
1/10/2010	-0.0049
1/11/2010	-0.0005
1/12/2010	0.0029
1/13/2010	-0.0003
1/14/2010	0.0036
1/15/2010	0.0063
1/16/2010	0.0028
1/17/2010	0.0023
1/18/2010	0.0066
1/19/2010	0.0044
1/20/2010	0.0056
1/21/2010	0.0120
1/22/2010	0.0098
1/23/2010	0.0073
1/24/2010	0.0094
1/25/2010	0.0086
1/26/2010	0.0093
1/27/2010	0.0116
1/28/2010	0.0125
1/29/2010	0.0096
1/30/2010	0.0118
1/31/2010	0.0115
2/1/2010	0.0071
2/2/2010	0.0064
2/3/2010	0.0040
2/4/2010	0.0078
2/5/2010	0.0009
2/6/2010	0.0093
2/7/2010	0.0104
2/8/2010	0.0057
2/9/2010	0.0116
2/10/2010	0.0119
2/11/2010	0.0125
2/12/2010	0.0137
2/13/2010	0.0119
2/15/2010	0.0090
2/16/2010	0.0085
2/17/2010	0.0064
2/18/2010	0.0057
2/19/2010	0.0058
2/20/2010	0.0040
2/21/2010	0.0049
2/22/2010	0.0038
2/24/2010	0.0020

2/25/2010	0.0016
2/26/2010	0.0046
2/27/2010	0.0020
2/28/2010	0.0047
3/1/2010	0.0076
3/2/2010	0.0071
3/3/2010	0.0017
3/4/2010	0.0078
3/5/2010	0.0048
3/6/2010	0.0095
3/7/2010	0.0079
3/8/2010	0.0105
3/9/2010	0.0118
3/10/2010	0.0120
3/11/2010	0.0083
3/12/2010	0.0049
3/13/2010	0.0093
3/14/2010	0.0098
3/15/2010	0.0059
3/16/2010	0.0048
3/17/2010	0.0057
3/18/2010	0.0047
3/19/2010	0.0065
3/20/2010	0.0062
3/21/2010	0.0085
3/22/2010	0.0059
3/23/2010	0.0026
3/24/2010	0.0087
3/25/2010	0.0108
3/26/2010	0.0042
3/27/2010	0.0046
3/28/2010	0.0068
3/29/2010	0.0043
3/30/2010	0.0062
3/31/2010	-0.0011
4/1/2010	0.0101
4/2/2010	0.0031
4/3/2010	0.0032
4/4/2010	0.0032
4/5/2010	0.0078
4/6/2010	0.0043
4/7/2010	0.0059
4/8/2010	0.0070
4/9/2010	0.0092
4/10/2010	0.0042
4/11/2010	0.0097
4/12/2010	0.0084
4/13/2010	0.0029
4/14/2010	0.0057
4/15/2010	0.0073

4/16/2010	0.0063
4/17/2010	0.0050
4/18/2010	0.0043
4/19/2010	0.0031
4/20/2010	0.0079
4/21/2010	0.0081
4/22/2010	0.0039
4/23/2010	0.0071
4/26/2010	0.0097
4/27/2010	0.0075
4/28/2010	0.0052
4/29/2010	0.0087
4/30/2010	0.0130
5/1/2010	0.0049
5/2/2010	0.0067
5/3/2010	0.0063
5/4/2010	0.0060
5/5/2010	0.0095
5/6/2010	0.0043
5/7/2010	0.0085
5/8/2010	0.0058
5/9/2010	0.0022
5/10/2010	0.0044
5/11/2010	0.0071
5/12/2010	0.0055
5/13/2010	0.0055
5/14/2010	0.0079
5/15/2010	0.0013
5/16/2010	-0.0012
5/17/2010	0.0049
5/18/2010	0.0073
5/19/2010	0.0077
5/20/2010	0.0064
5/21/2010	0.0102
5/22/2010	0.0070
5/23/2010	0.0065
5/24/2010	0.0097
5/25/2010	0.0046
5/26/2010	0.0066
5/27/2010	0.0089
5/28/2010	0.0094
5/29/2010	0.0090
5/30/2010	0.0049
5/31/2010	0.0045
6/1/2010	0.0037
6/2/2010	0.0082
6/3/2010	0.0047
6/4/2010	0.0081
6/5/2010	0.0082
6/6/2010	0.0067

6/7/2010	0.0046
6/8/2010	0.0047
6/9/2010	0.0014
6/10/2010	0.0055
6/11/2010	0.0054
6/12/2010	0.0064
6/13/2010	0.0090
6/14/2010	0.0045
6/15/2010	0.0076
6/16/2010	0.0099
6/17/2010	0.0090
6/18/2010	0.0114
6/19/2010	0.0081
6/20/2010	0.0132
6/21/2010	0.0104
6/22/2010	0.0038
6/23/2010	0.0047
6/24/2010	0.0075
6/25/2010	0.0060
6/26/2010	0.0092
6/27/2010	0.0032
6/28/2010	0.0079
6/29/2010	0.0051
6/30/2010	0.0174
7/1/2010	0.0041
7/2/2010	0.0047
7/3/2010	0.0109
7/4/2010	0.0173
7/5/2010	0.0121
7/6/2010	0.0164
7/7/2010	0.0131
7/8/2010	0.0082
7/9/2010	0.0053
7/10/2010	0.0076
7/11/2010	0.0052
7/12/2010	0.0122
7/13/2010	0.0121
7/14/2010	0.0107
7/15/2010	0.0135
7/16/2010	0.0062
7/17/2010	0.0143
7/18/2010	0.0106
7/19/2010	0.0174
7/20/2010	0.0114
7/21/2010	0.0067
7/22/2010	0.0155
7/23/2010	0.0207
7/24/2010	0.0131
7/25/2010	0.0215
7/26/2010	0.0118

7/27/2010	0.0107
7/28/2010	0.0040
7/29/2010	0.0133
7/30/2010	0.0193
7/31/2010	0.0139
8/1/2010	0.0152
8/2/2010	0.0148
8/3/2010	0.0196
8/4/2010	0.0221
8/5/2010	0.0194
8/6/2010	0.0138
8/7/2010	0.0156
8/8/2010	0.0170
8/9/2010	0.0233
8/10/2010	0.0162
8/11/2010	0.0199
8/12/2010	0.0122
8/13/2010	0.0179
8/14/2010	0.0184
8/15/2010	0.0174
8/16/2010	0.0177
8/17/2010	0.0166
8/18/2010	0.0136
8/19/2010	0.0138
8/20/2010	0.0210
8/21/2010	0.0172
8/22/2010	0.0210
8/23/2010	0.0142
8/24/2010	0.0148
8/25/2010	0.0212
8/26/2010	0.0180
8/28/2010	0.0181
8/29/2010	0.0207
8/30/2010	0.0223
8/31/2010	0.0253
9/1/2010	0.0159
9/2/2010	0.0163
9/3/2010	0.0152
9/4/2010	0.0226
9/5/2010	0.0240
9/6/2010	0.0211
9/7/2010	0.0145
9/8/2010	0.0169
9/9/2010	0.0192
9/10/2010	0.0242
9/11/2010	0.0165
9/12/2010	0.0213
9/13/2010	0.0164
9/14/2010	0.0217
9/15/2010	0.0207

9/16/2010	0.0233
9/17/2010	0.0174
9/18/2010	0.0196
9/20/2010	0.0185
9/21/2010	0.0177
9/22/2010	0.0156
9/23/2010	0.0149
9/24/2010	0.0148
9/25/2010	0.0093
9/26/2010	0.0160
9/27/2010	0.0160
9/28/2010	0.0223
9/29/2010	0.0194
9/30/2010	0.0181
10/6/2010	0.0282
10/7/2010	0.0215
10/8/2010	0.0171
10/9/2010	0.0186
10/10/2010	0.0267
10/11/2010	0.0245
10/12/2010	0.0248
10/13/2010	0.0218
10/14/2010	0.0142
10/15/2010	0.0176
10/16/2010	0.0212
10/17/2010	0.0217
10/18/2010	0.0236
10/19/2010	0.0257
10/20/2010	0.0240
10/21/2010	0.0236
10/22/2010	0.0181
10/23/2010	0.0227
10/24/2010	0.0250
10/25/2010	0.0202
10/26/2010	0.0213
10/27/2010	0.0216
10/28/2010	0.0203
10/29/2010	0.0191
10/30/2010	0.0208
10/31/2010	0.0243
11/1/2010	0.0284
11/2/2010	0.0203
11/3/2010	0.0204
11/4/2010	0.0189
11/5/2010	0.0257
11/6/2010	0.0250
11/7/2010	0.0236
11/8/2010	0.0178
11/9/2010	0.0234
11/10/2010	0.0236

11/11/2010	0.0198
11/12/2010	0.0194
11/13/2010	0.0216
11/14/2010	0.0228
11/15/2010	0.0199
11/16/2010	0.0202
11/17/2010	0.0238
11/18/2010	0.0208
11/19/2010	0.0190
11/20/2010	0.0227
11/21/2010	0.0228
11/22/2010	0.0260
11/23/2010	0.0249
11/24/2010	0.0281
11/25/2010	0.0237
11/26/2010	0.0196
11/27/2010	0.0237
11/28/2010	0.0227
11/29/2010	0.0263
11/30/2010	0.0231
12/1/2010	0.0231
12/2/2010	0.0284
12/3/2010	0.0288
12/4/2010	0.0258
12/5/2010	0.0273
12/6/2010	0.0281
12/7/2010	0.0229
12/8/2010	0.0180
12/9/2010	0.0181
12/10/2010	0.0235
12/11/2010	0.0243
12/12/2010	0.0207
12/13/2010	0.0194
12/14/2010	0.0247
12/15/2010	0.0261
12/16/2010	0.0264
12/17/2010	0.0284
12/18/2010	0.0334
12/19/2010	0.0222
12/20/2010	0.0213
12/21/2010	0.0207
12/22/2010	0.0267
12/23/2010	0.0188
12/24/2010	0.0248
12/25/2010	0.0238
12/26/2010	0.0314
12/27/2010	0.0285
12/28/2010	0.0265
12/29/2010	0.0243
12/30/2010	0.0195

1/1/2011	0.0212
1/2/2011	0.0238
1/3/2011	0.0222
1/4/2011	0.0214
1/5/2011	0.0202
1/6/2011	0.0201
1/7/2011	0.0188
1/8/2011	0.0181
1/9/2011	0.0241
1/10/2011	0.0257
1/11/2011	0.0248
1/12/2011	0.0261
1/13/2011	0.0259
1/14/2011	0.0244
1/15/2011	0.0262
1/16/2011	0.0226
1/17/2011	0.0216
1/18/2011	0.0228
1/19/2011	0.0260
1/20/2011	0.0257
1/21/2011	0.0211
1/22/2011	0.0262
1/23/2011	0.0254
1/24/2011	0.0228
1/25/2011	0.0243
1/26/2011	0.0244
1/27/2011	0.0221
1/28/2011	0.0254
1/29/2011	0.0199
1/30/2011	0.0188
1/31/2011	0.0240
2/1/2011	0.0235
2/2/2011	0.0255
2/3/2011	0.0232
2/4/2011	0.0252
2/5/2011	0.0231
2/6/2011	0.0233
2/7/2011	0.0209
2/8/2011	0.0239
2/9/2011	0.0242
2/10/2011	0.0233
2/11/2011	0.0206
2/12/2011	0.0233
2/13/2011	0.0254
2/14/2011	0.0251
2/15/2011	0.0248
2/16/2011	0.0272
2/17/2011	0.0260
2/18/2011	0.0289
2/19/2011	0.0245

2/20/2011	0.0244
2/21/2011	0.0265
2/22/2011	0.0253
2/23/2011	0.0258
2/24/2011	0.0242
2/25/2011	0.0215
2/26/2011	0.0226
2/27/2011	0.0219
2/28/2011	0.0207
3/1/2011	0.0219
3/2/2011	0.0247
3/3/2011	0.0201
3/4/2011	0.0235
3/5/2011	0.0215
3/6/2011	0.0181
3/7/2011	0.0201
3/8/2011	0.0182
3/9/2011	0.0179
3/10/2011	0.0187
3/11/2011	0.0200
3/12/2011	0.0202
3/13/2011	0.0197
3/14/2011	0.0214
3/15/2011	0.0218
3/16/2011	0.0302
3/17/2011	0.0197
3/18/2011	0.0290
3/19/2011	0.0244
3/20/2011	0.0223
3/21/2011	0.0235
3/22/2011	0.0227
3/23/2011	0.0220
3/24/2011	0.0224
3/25/2011	0.0226
3/26/2011	0.0212
3/27/2011	0.0202
3/28/2011	0.0190
3/29/2011	0.0173
3/30/2011	0.0228
3/31/2011	0.0229
4/1/2011	0.0229
4/2/2011	0.0160
4/3/2011	0.0215
4/4/2011	0.0240
4/5/2011	0.0270
4/6/2011	0.0234
4/7/2011	0.0256
4/8/2011	0.0239
4/9/2011	0.0242
4/10/2011	0.0213

4/11/2011	0.0242
4/12/2011	0.0221
4/13/2011	0.0358
4/14/2011	0.0254
4/15/2011	0.0211
4/16/2011	0.0233
4/17/2011	0.0218
4/18/2011	0.0253
4/19/2011	0.0245
4/20/2011	0.0222
4/21/2011	0.0238
4/22/2011	0.0226
4/23/2011	0.0248
4/24/2011	0.0266
4/25/2011	0.0234
4/26/2011	0.0317
4/27/2011	0.0313
4/28/2011	0.0276
4/29/2011	0.0318
4/30/2011	0.0321
5/1/2011	0.0273
5/2/2011	0.0286
5/3/2011	0.0297
5/4/2011	0.0328
5/5/2011	0.0351
5/6/2011	0.0276
5/7/2011	0.0288
5/8/2011	0.0331
5/9/2011	0.0282
5/10/2011	0.0273
5/11/2011	0.0342
5/12/2011	0.0330
5/13/2011	0.0335
5/14/2011	0.0365
5/15/2011	0.0393
5/16/2011	0.0343
5/17/2011	0.0359
5/18/2011	0.0356
5/19/2011	0.0346
5/20/2011	0.0335
5/21/2011	0.0336
5/22/2011	0.0397
5/23/2011	0.0354
5/24/2011	0.0424
5/25/2011	0.0395
5/26/2011	0.0430
5/27/2011	0.0395
5/28/2011	0.0398
5/29/2011	0.0441
5/30/2011	0.0419

5/31/2011	0.0406
6/1/2011	0.0354
6/2/2011	0.0340
6/3/2011	0.0429
6/4/2011	0.0403
6/5/2011	0.0429
6/6/2011	0.0433
6/7/2011	0.0426
6/8/2011	0.0390
6/9/2011	0.0500
6/10/2011	0.0470
6/11/2011	0.0441
6/12/2011	0.0422
6/13/2011	0.0460
6/14/2011	0.0391
6/15/2011	0.0388
6/16/2011	0.0413
6/17/2011	0.0420
6/18/2011	0.0456
6/19/2011	0.0455
6/20/2011	0.0449
6/21/2011	0.0423
6/22/2011	0.0452
6/23/2011	0.0457
6/24/2011	0.0475
6/25/2011	0.0500
6/26/2011	0.0518
6/27/2011	0.0462
6/28/2011	0.0452
6/29/2011	0.0502
6/30/2011	0.0491
7/1/2011	0.0439
7/2/2011	0.0533
7/3/2011	0.0402
7/4/2011	0.0440
7/5/2011	0.0512
7/6/2011	0.0391
7/7/2011	0.0447
7/8/2011	0.0403
7/9/2011	0.0458
7/10/2011	0.0429
7/11/2011	0.0452
7/12/2011	0.0471
7/13/2011	0.0449
7/14/2011	0.0514
7/15/2011	0.0485
7/16/2011	0.0488
7/17/2011	0.0441
7/18/2011	0.0393
7/19/2011	0.0480

7/20/2011	0.0419
7/21/2011	0.0436
7/22/2011	0.0461
7/23/2011	0.0502
7/24/2011	0.0400
7/25/2011	0.0466
7/26/2011	0.0459
7/27/2011	0.0449
7/28/2011	0.0549
7/29/2011	0.0489
7/30/2011	0.0435
7/31/2011	0.0483
8/1/2011	0.0460
8/2/2011	0.0451
8/3/2011	0.0448
8/4/2011	0.0480
8/5/2011	0.0467
8/6/2011	0.0432
8/7/2011	0.0429
8/8/2011	0.0469
8/9/2011	0.0514
8/10/2011	0.0515
8/11/2011	0.0485
8/12/2011	0.0479
8/13/2011	0.0466
8/14/2011	0.0465
8/15/2011	0.0454
8/16/2011	0.0536
8/17/2011	0.0495
8/18/2011	0.0487
8/19/2011	0.0513
8/20/2011	0.0533
8/21/2011	0.0487
8/22/2011	0.0461
8/23/2011	0.0493
8/24/2011	0.0484
8/25/2011	0.0523
8/26/2011	0.0504
8/27/2011	0.0516
8/28/2011	0.0582
8/29/2011	0.0545
8/30/2011	0.0530
8/31/2011	0.0523
9/1/2011	0.0472
9/2/2011	0.0561
9/3/2011	0.0479
9/4/2011	0.0487
9/5/2011	0.0532
9/6/2011	0.0527
9/7/2011	0.0503

9/8/2011	0.0551
9/9/2011	0.0527
9/10/2011	0.0494
9/11/2011	0.0498
9/12/2011	0.0509
9/13/2011	0.0540
9/14/2011	0.0521
9/15/2011	0.0493
9/16/2011	0.0514
9/17/2011	0.0530
9/18/2011	0.0546
9/19/2011	0.0561
9/20/2011	0.0485
9/21/2011	0.0546
9/22/2011	0.0593
9/23/2011	0.0569
9/24/2011	0.0551
9/25/2011	0.0552
9/26/2011	0.0574
9/27/2011	0.0528
9/28/2011	0.0598
9/29/2011	0.0652
9/30/2011	0.0616
10/1/2011	0.0537
10/2/2011	0.0582
10/3/2011	0.0604
10/4/2011	0.0539
10/5/2011	0.0560
10/6/2011	0.0604
10/7/2011	0.0569
10/8/2011	0.0568
10/9/2011	0.0547
10/10/2011	0.0568
10/11/2011	0.0571
10/12/2011	0.0588
10/13/2011	0.0637
10/14/2011	0.0666
10/15/2011	0.0629
10/16/2011	0.0666
10/17/2011	0.0600
10/18/2011	0.0589
10/19/2011	0.0590
10/20/2011	0.0650
10/21/2011	0.0741
10/22/2011	0.0632
10/23/2011	0.0570
10/24/2011	0.0631
10/25/2011	0.0568
10/26/2011	0.0539
10/27/2011	0.0560

10/28/2011	0.0563
10/29/2011	0.0556
10/30/2011	0.0610
10/31/2011	0.0568
11/1/2011	0.0522
11/2/2011	0.0579
11/3/2011	0.0552
11/4/2011	0.0562
11/5/2011	0.0570
11/6/2011	0.0647
11/7/2011	0.0585
11/8/2011	0.0561
11/9/2011	0.0594
11/10/2011	0.0607
11/11/2011	0.0593
11/12/2011	0.0634
11/13/2011	0.0702
11/14/2011	0.0650
11/15/2011	0.0617
11/16/2011	0.0606
11/17/2011	0.0608
11/18/2011	0.0645
11/19/2011	0.0625
11/20/2011	0.0585
11/21/2011	0.0605
11/22/2011	0.0641
11/23/2011	0.0656
11/24/2011	0.0630
11/25/2011	0.0638
11/26/2011	0.0653
11/27/2011	0.0657
11/28/2011	0.0645
11/29/2011	0.0642
11/30/2011	0.0661
12/1/2011	0.0631
12/2/2011	0.0622
12/3/2011	0.0662
12/4/2011	0.0631
12/5/2011	0.0613
12/6/2011	0.0620
12/7/2011	0.0612
12/8/2011	0.0642
12/9/2011	0.0612
12/10/2011	0.0640
12/11/2011	0.0581
12/12/2011	0.0559
12/13/2011	0.0579
12/14/2011	0.0662
12/15/2011	0.0681
12/16/2011	0.0655

12/17/2011	0.0614
12/18/2011	0.0649
12/19/2011	0.0632
12/20/2011	0.0647
12/21/2011	0.0632
12/22/2011	0.0617
12/23/2011	0.0692
12/24/2011	0.0654
12/25/2011	0.0577
12/26/2011	0.0633
12/27/2011	0.0608
12/28/2011	0.0592
12/29/2011	0.0612
12/30/2011	0.0635
12/31/2011	0.0657
1/1/2012	0.0625
1/2/2012	0.0645
1/3/2012	0.0611
1/4/2012	0.0621
1/5/2012	0.0614
1/6/2012	0.0696
1/7/2012	0.0655
1/8/2012	0.0624
1/9/2012	0.0603
1/10/2012	0.0637
1/11/2012	0.0558
1/12/2012	0.0627
1/13/2012	0.0711
1/14/2012	0.0646
1/15/2012	0.0644
1/16/2012	0.0681
1/17/2012	0.0656
1/18/2012	0.0641
1/19/2012	0.0620
1/20/2012	0.0691
1/21/2012	0.0642
1/22/2012	0.0662
1/23/2012	0.0663
1/24/2012	0.0667
1/25/2012	0.0631
1/26/2012	0.0654
1/27/2012	0.0565
1/28/2012	0.0664
1/29/2012	0.0684
1/30/2012	0.0678
1/31/2012	0.0693
2/1/2012	0.0673
2/2/2012	0.0665
2/3/2012	0.0667
2/4/2012	0.0699

2/5/2012	0.0654
2/6/2012	0.0685
2/7/2012	0.0718
2/8/2012	0.0704
2/9/2012	0.0689
2/10/2012	0.0659
2/11/2012	0.0667
2/12/2012	0.0654
2/13/2012	0.0702
2/14/2012	0.0683
2/15/2012	0.0623
2/16/2012	0.0704
2/17/2012	0.0656
2/18/2012	0.0627
2/19/2012	0.0680
2/20/2012	0.0626
2/21/2012	0.0644
2/22/2012	0.0698
2/23/2012	0.0691
2/24/2012	0.0682
2/25/2012	0.0675
2/26/2012	0.0706
2/27/2012	0.0738
2/28/2012	0.0668
2/29/2012	0.0676
3/1/2012	0.0716
3/2/2012	0.0657
3/3/2012	0.0658
3/4/2012	0.0657
3/5/2012	0.0705
3/6/2012	0.0666
3/7/2012	0.0695
3/8/2012	0.0785
3/9/2012	0.0749
3/10/2012	0.0740
3/11/2012	0.0625
3/12/2012	0.0735
3/13/2012	0.0713
3/14/2012	0.0701
3/15/2012	0.0672
3/16/2012	0.0700
3/17/2012	0.0657
3/18/2012	0.0701
3/19/2012	0.0656
3/20/2012	0.0695
3/21/2012	0.0703
3/22/2012	0.0734
3/23/2012	0.0694
3/24/2012	0.0710
3/25/2012	0.0710

3/26/2012	0.0746
3/27/2012	0.0739
3/28/2012	0.0727
3/29/2012	0.0677
3/30/2012	0.0725
3/31/2012	0.0755
4/1/2012	0.0721
4/2/2012	0.0657
4/3/2012	0.0638
4/4/2012	0.0691
4/5/2012	0.0685
4/6/2012	0.0699
4/7/2012	0.0696
4/8/2012	0.0722
4/9/2012	0.0687
4/10/2012	0.0683
4/11/2012	0.0697
4/12/2012	0.0645
4/13/2012	0.0708
4/14/2012	0.0679
4/15/2012	0.0665
4/16/2012	0.0672
4/17/2012	0.0734
4/18/2012	0.0695
4/19/2012	0.0672
4/20/2012	0.0686
4/21/2012	0.0659
4/22/2012	0.0675
4/23/2012	0.0759
4/24/2012	0.0727
4/25/2012	0.0703
4/26/2012	0.0656
4/27/2012	0.0711
4/28/2012	0.0699
4/29/2012	0.0605
4/30/2012	0.0703
5/1/2012	0.0714
5/2/2012	0.0759
5/3/2012	0.0707
5/4/2012	0.0690
5/5/2012	0.0723
5/6/2012	0.0671
5/7/2012	0.0711
5/8/2012	0.0643
5/9/2012	0.0681
5/10/2012	0.0693
5/11/2012	0.0761
5/12/2012	0.0730
5/13/2012	0.0782
5/14/2012	0.0741

5/15/2012	0.0751
5/16/2012	0.0716
5/17/2012	0.0726
5/18/2012	0.0716
5/19/2012	0.0703
5/20/2012	0.0740
5/21/2012	0.0723
5/22/2012	0.0665
5/23/2012	0.0726
5/24/2012	0.0748
5/25/2012	0.0720
5/26/2012	0.0760
5/27/2012	0.0718
5/28/2012	0.0760
5/29/2012	0.0731
5/30/2012	0.0771
5/31/2012	0.0790
6/1/2012	0.0814
6/2/2012	0.0758
6/3/2012	0.0719
6/4/2012	0.0793
6/5/2012	0.0804
6/6/2012	0.0776
6/7/2012	0.0741
6/8/2012	0.0770
6/9/2012	0.0753
6/10/2012	0.0815
6/11/2012	0.0759
6/12/2012	0.0726
6/13/2012	0.0760
6/14/2012	0.0816
6/15/2012	0.0775
6/16/2012	0.0804
6/17/2012	0.0822
6/18/2012	0.0842
6/19/2012	0.0672
6/20/2012	0.0831
6/21/2012	0.0822
6/22/2012	0.0813
6/23/2012	0.0810
6/24/2012	0.0850
6/25/2012	0.0851
6/26/2012	0.0814
6/27/2012	0.0840
6/28/2012	0.0850
6/29/2012	0.0850
6/30/2012	0.0825
7/1/2012	0.0914
7/2/2012	0.0809
7/3/2012	0.0841

7/4/2012	0.0884
7/5/2012	0.0817
7/6/2012	0.0840
7/7/2012	0.0806
7/8/2012	0.0838
7/9/2012	0.0894
7/10/2012	0.0878
7/11/2012	0.0867
7/12/2012	0.0923
7/13/2012	0.0866
7/14/2012	0.0883
7/15/2012	0.0944
7/16/2012	0.0912
7/17/2012	0.0890
7/18/2012	0.0869
7/19/2012	0.0890
7/20/2012	0.0864
7/21/2012	0.0925
7/22/2012	0.0910
7/23/2012	0.0973
7/24/2012	0.0909
7/25/2012	0.0900
7/26/2012	0.0906
7/27/2012	0.0923
7/28/2012	0.0924
7/29/2012	0.0929
7/30/2012	0.0937
7/31/2012	0.0957
8/1/2012	0.1044
8/2/2012	0.0977
8/3/2012	0.1016
8/4/2012	0.1017
8/5/2012	0.0985
8/6/2012	0.0970
8/7/2012	0.1047
8/8/2012	0.1033
8/9/2012	0.1143
8/10/2012	0.1180
8/11/2012	0.1076
8/12/2012	0.1081
8/13/2012	0.1015
8/14/2012	0.1053
8/15/2012	0.1076
8/17/2012	0.1092
8/18/2012	0.1100
8/19/2012	0.1094
8/20/2012	0.1166
8/21/2012	0.1099
8/22/2012	0.1142
8/24/2012	0.1146

8/25/2012	0.1161
8/26/2012	0.1123
8/27/2012	0.1193
8/28/2012	0.1112
8/29/2012	0.1133
8/30/2012	0.1198
8/31/2012	0.1169
9/1/2012	0.1216
9/2/2012	0.1230
9/3/2012	0.1102
9/4/2012	0.1256
9/5/2012	0.1124
9/6/2012	0.1209
9/7/2012	0.1196
9/8/2012	0.1248
9/9/2012	0.1226
9/10/2012	0.1237
9/11/2012	0.1180
9/12/2012	0.1194
9/13/2012	0.1233
9/14/2012	0.1293
9/15/2012	0.1278
9/16/2012	0.1230
9/17/2012	0.1237
9/18/2012	0.1239
9/19/2012	0.1225
9/20/2012	0.1272
9/21/2012	0.1272
9/22/2012	0.1284
9/23/2012	0.1238
9/24/2012	0.1263
9/25/2012	0.1233
9/26/2012	0.1285
9/27/2012	0.1335
9/28/2012	0.1281
9/29/2012	0.1275
9/30/2012	0.1231
10/1/2012	0.1272
10/2/2012	0.1331
10/3/2012	0.1296
10/4/2012	0.1271
10/5/2012	0.1283
10/6/2012	0.1299
10/7/2012	0.1303
10/8/2012	0.1326
10/9/2012	0.1245
10/10/2012	0.1340
10/11/2012	0.1295
10/12/2012	0.1362
10/13/2012	0.1318

10/14/2012	0.1340
10/15/2012	0.1271
10/16/2012	0.1253
10/17/2012	0.1319
10/18/2012	0.1320
10/19/2012	0.1279
10/20/2012	0.1322
10/21/2012	0.1304
10/22/2012	0.1284
10/23/2012	0.1339
10/24/2012	0.1305
10/25/2012	0.1307
10/26/2012	0.1421
10/27/2012	0.1426
10/28/2012	0.1421
10/30/2012	0.1295
10/31/2012	0.1361
11/1/2012	0.1266
11/2/2012	0.1341
11/3/2012	0.1262
11/4/2012	0.1369
11/5/2012	0.1288
11/6/2012	0.1256
11/7/2012	0.1422
11/8/2012	0.1339
11/9/2012	0.1327
11/10/2012	0.1290
11/11/2012	0.1420
11/12/2012	0.1405
11/13/2012	0.1372
11/14/2012	0.1365
11/15/2012	0.1390
11/16/2012	0.1324
11/17/2012	0.1404
11/18/2012	0.1437
11/19/2012	0.1328
11/20/2012	0.1434
11/21/2012	0.1383
11/22/2012	0.1341
11/23/2012	0.1390
11/24/2012	0.1354
11/25/2012	0.1308
11/26/2012	0.1312
11/27/2012	0.1331
11/28/2012	0.1384
11/29/2012	0.1399
11/30/2012	0.1347
12/1/2012	0.1330
12/2/2012	0.1309
12/3/2012	0.1275

12/4/2012	0.1309
12/5/2012	0.1352
12/6/2012	0.1364
12/7/2012	0.1348
12/8/2012	0.1414
12/9/2012	0.1428
12/10/2012	0.1388
12/11/2012	0.1427
12/12/2012	0.1374
12/13/2012	0.1376
12/14/2012	0.1393
12/15/2012	0.1421
12/16/2012	0.1488
12/17/2012	0.1532
12/18/2012	0.1469
12/19/2012	0.1491
12/21/2012	0.1508
12/22/2012	0.1548
12/23/2012	0.1501
12/24/2012	0.1432
12/25/2012	0.1493
12/26/2012	0.1499
12/27/2012	0.1505
12/28/2012	0.1493
12/29/2012	0.1536
12/30/2012	0.1591
12/31/2012	0.1531
1/1/2013	0.1569
1/2/2013	0.1567
1/3/2013	0.1569
1/4/2013	0.1602
1/5/2013	0.1595
1/6/2013	0.1649
1/7/2013	0.1641
1/8/2013	0.1644
1/9/2013	0.1627
1/10/2013	0.1624
1/11/2013	0.1655
1/12/2013	0.1625
1/13/2013	0.1625
1/14/2013	0.1679
1/15/2013	0.1685
1/16/2013	0.1729
1/17/2013	0.1730
1/18/2013	0.1615
1/19/2013	0.1682
1/20/2013	0.1651
1/21/2013	0.1608
1/22/2013	0.1664
1/23/2013	0.1758

1/24/2013	0.1644
1/25/2013	0.1719
1/26/2013	0.1662
1/27/2013	0.1633
1/28/2013	0.1691
1/29/2013	0.1641
1/30/2013	0.1687
1/31/2013	0.1671
2/1/2013	0.1735
2/2/2013	0.1785
2/3/2013	0.1707
2/4/2013	0.1682
2/5/2013	0.1721
2/6/2013	0.1731
2/7/2013	0.1726
2/8/2013	0.1760
2/9/2013	0.1791
2/10/2013	0.1743
2/11/2013	0.1778
2/12/2013	0.1793
2/13/2013	0.1816
2/14/2013	0.1765
2/15/2013	0.1727
2/16/2013	0.1799
2/17/2013	0.1779
2/19/2013	0.1757
2/20/2013	0.1782
2/21/2013	0.1771
2/22/2013	0.1825
2/23/2013	0.1843
2/24/2013	0.1832
2/25/2013	0.1770
2/26/2013	0.1779
2/27/2013	0.1787
2/28/2013	0.1782
3/1/2013	0.1733
3/2/2013	0.1797
3/3/2013	0.1777
3/4/2013	0.1729
3/5/2013	0.1712
3/6/2013	0.1788
3/7/2013	0.1736
3/8/2013	0.1740
3/9/2013	0.1742
3/10/2013	0.1755
3/11/2013	0.1767
3/12/2013	0.1749
3/13/2013	0.1746
3/14/2013	0.1839
3/15/2013	0.1767

3/16/2013	0.1737
3/17/2013	0.1727
3/18/2013	0.1765
3/19/2013	0.1740
3/20/2013	0.1759
3/21/2013	0.1789
3/22/2013	0.1760
3/23/2013	0.1732
3/24/2013	0.1777
3/26/2013	0.1864
3/27/2013	0.1890
3/28/2013	0.1807
3/29/2013	0.1850
3/30/2013	0.1835
3/31/2013	0.1798
4/1/2013	0.1787
4/2/2013	0.1831
4/3/2013	0.1817
4/4/2013	0.1885
4/5/2013	0.1856
4/6/2013	0.1786
4/7/2013	0.1811
4/8/2013	0.1814
4/9/2013	0.1793
4/10/2013	0.1790
4/11/2013	0.1828
4/12/2013	0.1817
4/13/2013	0.1819
4/14/2013	0.1805
4/15/2013	0.1832
4/16/2013	0.1821
4/17/2013	0.1859
4/18/2013	0.1842
4/19/2013	0.1909
4/20/2013	0.1895
4/21/2013	0.1837
4/22/2013	0.1900
4/23/2013	0.1784
4/24/2013	0.1871
4/25/2013	0.1900
4/26/2013	0.1875
4/27/2013	0.1901
4/28/2013	0.1873
4/29/2013	0.1877
4/30/2013	0.1784
5/1/2013	0.1837
5/2/2013	0.1820
5/3/2013	0.1893
5/4/2013	0.1864
5/5/2013	0.1879

5/6/2013	0.1890
5/7/2013	0.1847
5/8/2013	0.1835
5/9/2013	0.1842
5/10/2013	0.1813
5/11/2013	0.1853
5/12/2013	0.1868
5/13/2013	0.1782
5/14/2013	0.1848
5/15/2013	0.1830
5/16/2013	0.1839
5/17/2013	0.1850
5/18/2013	0.1838
5/19/2013	0.1751
5/20/2013	0.1839
5/21/2013	0.1843
5/22/2013	0.1939
5/23/2013	0.1789
5/24/2013	0.1838
5/25/2013	0.1845
5/26/2013	0.1860
5/27/2013	0.1790
5/28/2013	0.1844
5/29/2013	0.1830
5/30/2013	0.1793
5/31/2013	0.1771
6/1/2013	0.1851
6/2/2013	0.1863
6/3/2013	0.1857
6/4/2013	0.1792
6/5/2013	0.1844
6/6/2013	0.1830
6/7/2013	0.1869
6/8/2013	0.1879
6/9/2013	0.1853
6/10/2013	0.1839
6/11/2013	0.1851
6/12/2013	0.1786
6/13/2013	0.1870
6/14/2013	0.1835
6/15/2013	0.1801
6/16/2013	0.1856
6/17/2013	0.1792
6/18/2013	0.1843
6/19/2013	0.1800
6/20/2013	0.1789
6/21/2013	0.1873
6/22/2013	0.1851
6/23/2013	0.1890
6/24/2013	0.1871

6/25/2013	0.1845
6/26/2013	0.1852
6/27/2013	0.1783
6/28/2013	0.1885
6/29/2013	0.1861
6/30/2013	0.1833
7/1/2013	0.1849
7/2/2013	0.1837
7/3/2013	0.1800
7/4/2013	0.1812
7/5/2013	0.1880
7/6/2013	0.1890
7/7/2013	0.1835
7/8/2013	0.1862
7/9/2013	0.1876
7/10/2013	0.1814
7/11/2013	0.1838
7/12/2013	0.1866
7/13/2013	0.1840
7/14/2013	0.1833
7/15/2013	0.1835
7/16/2013	0.1813
7/17/2013	0.1780
7/18/2013	0.1734
7/19/2013	0.1819
7/20/2013	0.1735
7/21/2013	0.1861
7/22/2013	0.1839
7/23/2013	0.1807
7/24/2013	0.1825
7/25/2013	0.1828
7/26/2013	0.1858
7/27/2013	0.1832
7/28/2013	0.1788
7/29/2013	0.1862
7/30/2013	0.1787
7/31/2013	0.1922
8/1/2013	0.1858
8/2/2013	0.1823
8/3/2013	0.1797
8/4/2013	0.1776
8/5/2013	0.1804
8/6/2013	0.1824
8/7/2013	0.1874
8/8/2013	0.1846
8/9/2013	0.1814
8/10/2013	0.1919
8/11/2013	0.1896
8/12/2013	0.1795
8/13/2013	0.1825

8/14/2013	0.1823
8/15/2013	0.1778
8/16/2013	0.1877
8/17/2013	0.1816
8/18/2013	0.1817
8/19/2013	0.1803
8/20/2013	0.1827
8/21/2013	0.1868
8/22/2013	0.1804
8/23/2013	0.1835
8/24/2013	0.1790
8/25/2013	0.1765
8/26/2013	0.1799
8/27/2013	0.1840
8/28/2013	0.1764
8/29/2013	0.1815
8/30/2013	0.1798
8/31/2013	0.1803
9/1/2013	0.1883
9/2/2013	0.1792
9/3/2013	0.1805
9/4/2013	0.1831
9/5/2013	0.1762
9/6/2013	0.1657
9/7/2013	0.1707
9/8/2013	0.1827
9/9/2013	0.1889
9/10/2013	0.1838
9/11/2013	0.1800
9/12/2013	0.1888
9/13/2013	0.1869
9/14/2013	0.1784
9/15/2013	0.1816
9/17/2013	0.1902
9/18/2013	0.1829
9/19/2013	0.1867
9/20/2013	0.1807
9/21/2013	0.1842
9/23/2013	0.1770
9/24/2013	0.1796
9/25/2013	0.1801
9/26/2013	0.1791
9/27/2013	0.1788
9/28/2013	0.1749
9/30/2013	0.1730
10/1/2013	0.1825
10/2/2013	0.1821
10/3/2013	0.1802
10/4/2013	0.1803
10/5/2013	0.1809

10/6/2013	0.1778
10/7/2013	0.1840
10/8/2013	0.1689
10/9/2013	0.1814
10/10/2013	0.1826
10/11/2013	0.1828
10/12/2013	0.1907
10/13/2013	0.1798
10/14/2013	0.1807
10/15/2013	0.1782
10/16/2013	0.1819
10/17/2013	0.1816
10/18/2013	0.1794
10/19/2013	0.1775
10/20/2013	0.1761
10/21/2013	0.1794
10/22/2013	0.1765
10/23/2013	0.1792
10/24/2013	0.1804
10/25/2013	0.1785
10/26/2013	0.1766
10/27/2013	0.1769
10/28/2013	0.1791
10/29/2013	0.1850
10/30/2013	0.1790
10/31/2013	0.1827
11/1/2013	0.1803
11/2/2013	0.1808
11/3/2013	0.1872
11/4/2013	0.1845
11/5/2013	0.1876
11/6/2013	0.1817
11/7/2013	0.1791
11/8/2013	0.1789
11/9/2013	0.1776
11/10/2013	0.1788
11/11/2013	0.1825
11/12/2013	0.1792
11/13/2013	0.1786
11/14/2013	0.1810
11/15/2013	0.1808
11/16/2013	0.1809
11/17/2013	0.1795
11/18/2013	0.1757
11/19/2013	0.1811
11/20/2013	0.1789
11/21/2013	0.1822
11/22/2013	0.1803
11/23/2013	0.1826
11/24/2013	0.1802

11/25/2013	0.1810
11/26/2013	0.1772
11/27/2013	0.1782
11/28/2013	0.1790
11/29/2013	0.1799
11/30/2013	0.1888
12/1/2013	0.1787
12/2/2013	0.1799
12/3/2013	0.1854
12/4/2013	0.1808
12/5/2013	0.1805
12/6/2013	0.1821
12/7/2013	0.1806
12/8/2013	0.1783
12/9/2013	0.1808
12/10/2013	0.1844
12/11/2013	0.1816
12/12/2013	0.1825
12/13/2013	0.1780
12/14/2013	0.1794
12/15/2013	0.1811
12/16/2013	0.1771
12/18/2013	0.1787
12/19/2013	0.1745
12/20/2013	0.1785
12/21/2013	0.1780
12/22/2013	0.1770
12/23/2013	0.1817
12/24/2013	0.1746
12/25/2013	0.1745
12/26/2013	0.1818
12/27/2013	0.1833
12/28/2013	0.1776
12/29/2013	0.1763
12/30/2013	0.1787
12/31/2013	0.1767
1/1/2014	0.1759
1/2/2014	0.1746
1/3/2014	0.1733
1/4/2014	0.1749
1/5/2014	0.1783
1/6/2014	0.1746
1/7/2014	0.1745
1/8/2014	0.1768
1/9/2014	0.1743
1/10/2014	0.1758
1/11/2014	0.1779
1/12/2014	0.1806
1/13/2014	0.1763
1/14/2014	0.1837

1/15/2014	0.1797
1/16/2014	0.1768
1/17/2014	0.1798
1/18/2014	0.1762
1/19/2014	0.1756
1/30/2014	0.1885
1/31/2014	0.1826
2/1/2014	0.1912
2/2/2014	0.2027
2/3/2014	0.1915
2/4/2014	0.1785
2/5/2014	0.1778
2/6/2014	0.1768
2/7/2014	0.1784
2/8/2014	0.1785
2/9/2014	0.1812
2/11/2014	0.1840
2/12/2014	0.1818
2/13/2014	0.1769
2/14/2014	0.1782
2/15/2014	0.1815
2/16/2014	0.1841
2/17/2014	0.1835
2/18/2014	0.1815
2/19/2014	0.1850
2/20/2014	0.1820
2/21/2014	0.1868
2/22/2014	0.1844
2/23/2014	0.1792
2/24/2014	0.1797
2/25/2014	0.1792
2/26/2014	0.1656
2/27/2014	0.1821
2/28/2014	0.1809
3/1/2014	0.1819
3/2/2014	0.1795
3/3/2014	0.1820
3/4/2014	0.1841
3/5/2014	0.1844
3/6/2014	0.1851
3/7/2014	0.1753
3/8/2014	0.1819
3/9/2014	0.1859
3/10/2014	0.1832
3/11/2014	0.1816
3/12/2014	0.1833
3/13/2014	0.1827
3/14/2014	0.1799
3/15/2014	0.1829
3/16/2014	0.1881

3/17/2014	0.1783
3/18/2014	0.1821
3/19/2014	0.1826
3/20/2014	0.1770
3/21/2014	0.1817
3/22/2014	0.1797
3/23/2014	0.1908
3/24/2014	0.1846
3/25/2014	0.1838
3/26/2014	0.1861
3/27/2014	0.1903
3/28/2014	0.1803
3/29/2014	0.1845
3/30/2014	0.1801
3/31/2014	0.1854
4/1/2014	0.1837
4/2/2014	0.1800
4/3/2014	0.1794
4/4/2014	0.1842
4/5/2014	0.1785
4/6/2014	0.1836
4/7/2014	0.1794
4/8/2014	0.1884
4/9/2014	0.1784
4/10/2014	0.1840
4/11/2014	0.1840
4/12/2014	0.1850
4/13/2014	0.1866
4/14/2014	0.1851
4/15/2014	0.1886
4/16/2014	0.1847
4/17/2014	0.1835
4/18/2014	0.1831
4/19/2014	0.1846
4/20/2014	0.1809
4/21/2014	0.1867
4/22/2014	0.1810
4/23/2014	0.1878
4/24/2014	0.1854
4/25/2014	0.1862
4/26/2014	0.1851
4/27/2014	0.1779
4/28/2014	0.1753
4/29/2014	0.1815
4/30/2014	0.1809
5/1/2014	0.1789
5/2/2014	0.1848
5/3/2014	0.1820
5/4/2014	0.1836
5/5/2014	0.1838

5/6/2014	0.1819
5/7/2014	0.1851
5/8/2014	0.1838
5/9/2014	0.1830
5/10/2014	0.1820
5/11/2014	0.1871
5/12/2014	0.1805
5/13/2014	0.1862
5/14/2014	0.1886
5/15/2014	0.1820
5/16/2014	0.1858
5/17/2014	0.1882
5/18/2014	0.1823
5/19/2014	0.1828
5/20/2014	0.1850
5/21/2014	0.1865
5/22/2014	0.1881
5/23/2014	0.1908
5/24/2014	0.1855
5/25/2014	0.1913
5/26/2014	0.1938
5/27/2014	0.1913
5/28/2014	0.1862
5/29/2014	0.1863
5/30/2014	0.1869
5/31/2014	0.1920
6/1/2014	0.1863
6/2/2014	0.1911
6/3/2014	0.1923
6/4/2014	0.1878
6/5/2014	0.1899
6/6/2014	0.1873
6/7/2014	0.1839
6/8/2014	0.1860
6/9/2014	0.1851
6/10/2014	0.1927
6/11/2014	0.1920
6/12/2014	0.1855
6/13/2014	0.1848
6/14/2014	0.1897
6/15/2014	0.1894
6/16/2014	0.1947
6/17/2014	0.1910
6/18/2014	0.1790
6/19/2014	0.1865
6/20/2014	0.1868
6/21/2014	0.1864
6/22/2014	0.1835
6/23/2014	0.1902
6/24/2014	0.1883

6/25/2014	0.1830
6/26/2014	0.1911
6/27/2014	0.1867
6/28/2014	0.1859
6/29/2014	0.1885
6/30/2014	0.1876
7/1/2014	0.1863
7/2/2014	0.1872
7/3/2014	0.1935
7/4/2014	0.1897
7/5/2014	0.1865
7/6/2014	0.1926
7/7/2014	0.1854
7/8/2014	0.1929
7/9/2014	0.1998
7/10/2014	0.1879
7/11/2014	0.1966
7/12/2014	0.1990
7/13/2014	0.1935
7/14/2014	0.1881
7/15/2014	0.1908
7/16/2014	0.1882
7/17/2014	0.1944
7/18/2014	0.1876
7/19/2014	0.1934
7/20/2014	0.1936
7/21/2014	0.2006
7/22/2014	0.2091
7/23/2014	0.1907
7/24/2014	0.1947
7/25/2014	0.1938
7/26/2014	0.1952
7/27/2014	0.2097
7/28/2014	0.1977
7/29/2014	0.1943
7/30/2014	0.1992
7/31/2014	0.1964
8/1/2014	0.1936
8/2/2014	0.1971
8/3/2014	0.1945
8/4/2014	0.2001
8/5/2014	0.2035
8/6/2014	0.2033
8/7/2014	0.2043
8/8/2014	0.1988
8/9/2014	0.1981
8/10/2014	0.1992
8/11/2014	0.2044
8/12/2014	0.1991
8/13/2014	0.2005

8/14/2014	0.2057
8/15/2014	0.1946
8/16/2014	0.1959
8/17/2014	0.2047
8/18/2014	0.1982
8/19/2014	0.2010
8/20/2014	0.2081
8/21/2014	0.2051
8/22/2014	0.2027
8/23/2014	0.2062
8/24/2014	0.2014
8/25/2014	0.2064
8/26/2014	0.1986
8/27/2014	0.2038
8/28/2014	0.2047
8/29/2014	0.2004
8/30/2014	0.2100
8/31/2014	0.2133
9/1/2014	0.2095
9/2/2014	0.2103
9/3/2014	0.2064
9/4/2014	0.2115
9/5/2014	0.2091
9/6/2014	0.2091
9/7/2014	0.2078
9/8/2014	0.2055
9/9/2014	0.2103
9/10/2014	0.2146
9/11/2014	0.2142
9/12/2014	0.2102
9/13/2014	0.2063
9/14/2014	0.2146
9/15/2014	0.2088
9/16/2014	0.2123
9/17/2014	0.2110
9/18/2014	0.2121
9/19/2014	0.2145
9/20/2014	0.2152
9/21/2014	0.2187
9/22/2014	0.2179
9/23/2014	0.2223
9/24/2014	0.2261
9/25/2014	0.2257
9/26/2014	0.2190
9/27/2014	0.2220
9/28/2014	0.2232
9/29/2014	0.2172
9/30/2014	0.2179
10/1/2014	0.2227
10/2/2014	0.2272

10/3/2014	0.2207
10/4/2014	0.2212
10/5/2014	0.2218
10/6/2014	0.2193
10/7/2014	0.2201
10/8/2014	0.2191
10/9/2014	0.2184
10/10/2014	0.2216
10/11/2014	0.2247
10/12/2014	0.2199
10/13/2014	0.2203
10/14/2014	0.2248
10/15/2014	0.2235
10/16/2014	0.2215
10/17/2014	0.2246
10/18/2014	0.2256
10/19/2014	0.2243
10/20/2014	0.2273
10/21/2014	0.2249
10/22/2014	0.2257
10/23/2014	0.2269
10/24/2014	0.2306
10/25/2014	0.2255
10/26/2014	0.2258
10/27/2014	0.2246
10/28/2014	0.2273
10/29/2014	0.2252
10/30/2014	0.2271
10/31/2014	0.2325
11/1/2014	0.2232
11/2/2014	0.2260
11/3/2014	0.2294
11/4/2014	0.2200
11/5/2014	0.2211
11/6/2014	0.2281
11/7/2014	0.2301
11/8/2014	0.2287
11/9/2014	0.2209
11/10/2014	0.2253
11/11/2014	0.2268
11/12/2014	0.2227
11/13/2014	0.2274
11/14/2014	0.2260
11/15/2014	0.2208
11/16/2014	0.2260
11/17/2014	0.2375
11/18/2014	0.2233
11/19/2014	0.2187
11/20/2014	0.2283
11/21/2014	0.2274

11/22/2014	0.2266
11/23/2014	0.2281
11/24/2014	0.2304
11/25/2014	0.2305
11/26/2014	0.2278
11/27/2014	0.2260
11/28/2014	0.2296
11/29/2014	0.2293
11/30/2014	0.2298
12/1/2014	0.2346
12/2/2014	0.2283
12/3/2014	0.2260
12/4/2014	0.2282
12/5/2014	0.2278
12/6/2014	0.2296
12/7/2014	0.2274
12/8/2014	0.2313
12/9/2014	0.2340
12/10/2014	0.2285
12/11/2014	0.2292
12/12/2014	0.2299
12/13/2014	0.2274
12/14/2014	0.2285
12/15/2014	0.2342
12/16/2014	0.2315
12/17/2014	0.2286
12/18/2014	0.2299
12/19/2014	0.2310
12/20/2014	0.2346
12/21/2014	0.2292
12/22/2014	0.2302
12/23/2014	0.2316
12/24/2014	0.2309
12/25/2014	0.2316
12/26/2014	0.2350
12/27/2014	0.2310
12/28/2014	0.2422
12/29/2014	0.2397
12/30/2014	0.2344
12/31/2014	0.2374
1/1/2015	0.2405
1/2/2015	0.2363
1/3/2015	0.2380
1/4/2015	0.2386
1/5/2015	0.2326
1/6/2015	0.2342
1/7/2015	0.2340
1/8/2015	0.2328
1/9/2015	0.2361
1/10/2015	0.2359

1/11/2015	0.2341
1/12/2015	0.2360
1/13/2015	0.2354
1/14/2015	0.2345
1/15/2015	0.2354
1/16/2015	0.2336
1/17/2015	0.2442
1/18/2015	0.2365
1/19/2015	0.2408
1/20/2015	0.2417
1/21/2015	0.2414
1/22/2015	0.2444
1/23/2015	0.2455
1/24/2015	0.2416
1/25/2015	0.2433
1/26/2015	0.2414
1/27/2015	0.2436
1/28/2015	0.2451
1/29/2015	0.2400
1/30/2015	0.2475
1/31/2015	0.2401
2/1/2015	0.2456
2/2/2015	0.2379
2/3/2015	0.2419
2/4/2015	0.2417
2/5/2015	0.2430
2/6/2015	0.2423
2/7/2015	0.2404
2/8/2015	0.2420
2/9/2015	0.2395
2/10/2015	0.2467
2/11/2015	0.2394
2/12/2015	0.2393
2/13/2015	0.2402
2/14/2015	0.2469
2/15/2015	0.2415
2/16/2015	0.2433
2/17/2015	0.2437
2/18/2015	0.2436
2/19/2015	0.2410
2/20/2015	0.2431
2/21/2015	0.2467
2/22/2015	0.2459
2/23/2015	0.2438
2/24/2015	0.2380
2/25/2015	0.2434
2/26/2015	0.2395
2/27/2015	0.2399
2/28/2015	0.2438
3/1/2015	0.2377

3/2/2015	0.2395
3/3/2015	0.2440
3/4/2015	0.2457
3/5/2015	0.2475
3/6/2015	0.2345
3/7/2015	0.2444
3/8/2015	0.2421
3/9/2015	0.2425
3/10/2015	0.2426
3/11/2015	0.2439
3/12/2015	0.2477
3/13/2015	0.2430
3/14/2015	0.2430
3/15/2015	0.2429
3/16/2015	0.2458
3/17/2015	0.2437
3/18/2015	0.2474
3/19/2015	0.2478
3/20/2015	0.2478
3/21/2015	0.2442
3/22/2015	0.2449
3/23/2015	0.2453
3/24/2015	0.2437
3/25/2015	0.2430
3/26/2015	0.2431
3/27/2015	0.2483
3/28/2015	0.2458
3/29/2015	0.2447
3/30/2015	0.2488
3/31/2015	0.2447
4/1/2015	0.2475
4/2/2015	0.2486
4/3/2015	0.2466
4/4/2015	0.2494
4/5/2015	0.2492
4/6/2015	0.2479
4/7/2015	0.2464
4/8/2015	0.2467
4/9/2015	0.2494
4/10/2015	0.2422
4/11/2015	0.2455
4/12/2015	0.2463
4/13/2015	0.2533
4/14/2015	0.2454
4/15/2015	0.2459
4/16/2015	0.2479
4/17/2015	0.2488
4/18/2015	0.2482
4/19/2015	0.2480
4/20/2015	0.2508

4/21/2015	0.2487
4/22/2015	0.2522
4/23/2015	0.2492
4/24/2015	0.2465
4/25/2015	0.2470
4/26/2015	0.2514
4/27/2015	0.2487
4/28/2015	0.2494
4/29/2015	0.2503
4/30/2015	0.2494
5/1/2015	0.2439
5/2/2015	0.2505
5/3/2015	0.2508
5/4/2015	0.2507
5/5/2015	0.2474
5/6/2015	0.2491
5/7/2015	0.2536
5/8/2015	0.2531
5/9/2015	0.2494
5/10/2015	0.2567
5/11/2015	0.2490
5/12/2015	0.2553
5/13/2015	0.2524
5/14/2015	0.2552
5/15/2015	0.2554
5/16/2015	0.2610
5/17/2015	0.2578
5/18/2015	0.2549
5/19/2015	0.2524
5/20/2015	0.2544
5/21/2015	0.2576
5/22/2015	0.2563
5/23/2015	0.2541
5/24/2015	0.2541
5/25/2015	0.2505
5/26/2015	0.2597
5/27/2015	0.2527
5/28/2015	0.2523
5/29/2015	0.2506
5/30/2015	0.2526
5/31/2015	0.2503
6/1/2015	0.2490
6/2/2015	0.2485
6/3/2015	0.2588
6/4/2015	0.2507
6/5/2015	0.2603
6/6/2015	0.2523
6/7/2015	0.2537
6/8/2015	0.2593
6/9/2015	0.2562

6/10/2015	0.2553
6/11/2015	0.2566
6/12/2015	0.2548
6/13/2015	0.2552
6/14/2015	0.2548
6/15/2015	0.2557
6/16/2015	0.2563
6/17/2015	0.2552
6/18/2015	0.2612
6/19/2015	0.2581
6/20/2015	0.2614
6/21/2015	0.2562
6/22/2015	0.2587
6/23/2015	0.2605
6/24/2015	0.2588
6/25/2015	0.2580
6/26/2015	0.2604
6/27/2015	0.2590
6/28/2015	0.2596
6/29/2015	0.2569
6/30/2015	0.2589
7/1/2015	0.2580
7/2/2015	0.2570
7/3/2015	0.2633
7/4/2015	0.2645
7/5/2015	0.2598
7/6/2015	0.2594
7/7/2015	0.2602
7/8/2015	0.2645
7/9/2015	0.2618
7/10/2015	0.2655
7/11/2015	0.2616
7/12/2015	0.2650
7/13/2015	0.2670
7/14/2015	0.2605
7/15/2015	0.2678
7/16/2015	0.2629
7/17/2015	0.2570
7/18/2015	0.2594
7/19/2015	0.2643
7/20/2015	0.2649
7/21/2015	0.2621
7/23/2015	0.2562
7/24/2015	0.2571
7/25/2015	0.2586
7/26/2015	0.2629
7/27/2015	0.2555
7/28/2015	0.2594
7/29/2015	0.2653
7/30/2015	0.2662

7/31/2015	0.2601
8/1/2015	0.2656
8/2/2015	0.2639
8/3/2015	0.2600
8/4/2015	0.2650
8/5/2015	0.2608
8/6/2015	0.2639
8/7/2015	0.2551
8/8/2015	0.2694
8/9/2015	0.2613
8/10/2015	0.2604
8/11/2015	0.2613
8/12/2015	0.2660
8/13/2015	0.2684
8/14/2015	0.2649
8/15/2015	0.2673
8/16/2015	0.2630
8/17/2015	0.2731
8/18/2015	0.2704
8/19/2015	0.2718
8/20/2015	0.2672
8/21/2015	0.2691
8/22/2015	0.2720
8/23/2015	0.2680
8/24/2015	0.2647
8/25/2015	0.2687
8/26/2015	0.2720
8/27/2015	0.2729
8/28/2015	0.2697
8/29/2015	0.2720
8/30/2015	0.2732
8/31/2015	0.2742
9/1/2015	0.2760
9/2/2015	0.2822
9/3/2015	0.2727
9/4/2015	0.2787
9/5/2015	0.2720
9/6/2015	0.2776
9/7/2015	0.2820
9/8/2015	0.2788
9/9/2015	0.2765
9/10/2015	0.2766
9/11/2015	0.2815
9/12/2015	0.2801
9/13/2015	0.2830
9/14/2015	0.2847
9/15/2015	0.2886
9/16/2015	0.2820
9/17/2015	0.2828
9/18/2015	0.2855

9/19/2015	0.2812
9/20/2015	0.2813
9/21/2015	0.2863
9/22/2015	0.2814
9/23/2015	0.2877
9/24/2015	0.2870
9/25/2015	0.2856
9/26/2015	0.2859
9/27/2015	0.2872
9/28/2015	0.2871
9/29/2015	0.2916
9/30/2015	0.2905
10/1/2015	0.2921
10/2/2015	0.2881
10/3/2015	0.2852
10/4/2015	0.2898
10/5/2015	0.2918
10/6/2015	0.2901
10/7/2015	0.2853
10/8/2015	0.2868
10/9/2015	0.2843
10/10/2015	0.2919
10/11/2015	0.2855
10/12/2015	0.2886
10/13/2015	0.2932
10/15/2015	0.2822
10/16/2015	0.2842
10/17/2015	0.2865
10/18/2015	0.2866
10/19/2015	0.2831
10/20/2015	0.2899
10/21/2015	0.2881
10/22/2015	0.2896
10/23/2015	0.2943
10/24/2015	0.2897
10/25/2015	0.2866
10/26/2015	0.2833
10/27/2015	0.2880
10/28/2015	0.2864
10/29/2015	0.2833
10/30/2015	0.2817
10/31/2015	0.2927
11/1/2015	0.2838
11/2/2015	0.2876
11/3/2015	0.2889
11/4/2015	0.2862
11/5/2015	0.2880
11/6/2015	0.2848
11/7/2015	0.2884
11/8/2015	0.2887

11/10/2015	0.2885
11/11/2015	0.2898
11/12/2015	0.2889
11/13/2015	0.2903
11/14/2015	0.2897
11/15/2015	0.2937
11/16/2015	0.2913
11/17/2015	0.2912
11/18/2015	0.2909
11/19/2015	0.2867
11/20/2015	0.2877
11/21/2015	0.2989
11/22/2015	0.2886
11/23/2015	0.2936
11/24/2015	0.2903
11/25/2015	0.2951
11/26/2015	0.2987
11/27/2015	0.2915
11/28/2015	0.2864
11/29/2015	0.2901
11/30/2015	0.2921
12/1/2015	0.2877
12/2/2015	0.2926
12/3/2015	0.2929
12/4/2015	0.2919
12/5/2015	0.2921
12/6/2015	0.2908
12/7/2015	0.2893
12/8/2015	0.2899
12/9/2015	0.2936
12/10/2015	0.2965
12/11/2015	0.2951
12/12/2015	0.2950
12/13/2015	0.2946
12/14/2015	0.2917
12/15/2015	0.2940
12/16/2015	0.2924
12/17/2015	0.2914
12/18/2015	0.2914
12/19/2015	0.2910
12/20/2015	0.2903
12/21/2015	0.2937
12/22/2015	0.2910
12/23/2015	0.2939
12/24/2015	0.2935
12/25/2015	0.2976
12/26/2015	0.2970
12/27/2015	0.2936
12/28/2015	0.2966
12/29/2015	0.2962

12/30/2015	0.2981
12/31/2015	0.2987
1/1/2016	0.2950
1/2/2016	0.2964
1/3/2016	0.2948
1/4/2016	0.2988
1/5/2016	0.3049
1/6/2016	0.2925
1/7/2016	0.2972
1/8/2016	0.2997
1/9/2016	0.2975
1/10/2016	0.2925
1/11/2016	0.3005
1/12/2016	0.3032
1/13/2016	0.2961
1/14/2016	0.3012
1/15/2016	0.3009
1/16/2016	0.3060
1/17/2016	0.3069
1/18/2016	0.3042
1/19/2016	0.3017
1/20/2016	0.3007
1/21/2016	0.3004
1/22/2016	0.3025
1/23/2016	0.3035
1/24/2016	0.3072
1/26/2016	0.3020
1/27/2016	0.3003
1/28/2016	0.3025
1/29/2016	0.3013
1/30/2016	0.3053
1/31/2016	0.3015
2/1/2016	0.3048
2/2/2016	0.3057
2/3/2016	0.3090
2/4/2016	0.3093
2/5/2016	0.3091
2/6/2016	0.3015
2/7/2016	0.3005
2/8/2016	0.3028
2/9/2016	0.3006
2/10/2016	0.3027
2/11/2016	0.3010
2/12/2016	0.3014
2/13/2016	0.3052
2/14/2016	0.3044
2/15/2016	0.3069
2/16/2016	0.3094
2/17/2016	0.3060
2/18/2016	0.3031

2/19/2016	0.3049
2/20/2016	0.3060
2/21/2016	0.3054
2/22/2016	0.3096
2/23/2016	0.3070
2/24/2016	0.3115
2/25/2016	0.3088
2/26/2016	0.3100
2/27/2016	0.3101
2/28/2016	0.3160
2/29/2016	0.3265
3/1/2016	0.3159
3/2/2016	0.3126
3/3/2016	0.3129
3/4/2016	0.3074
3/5/2016	0.3091
3/6/2016	0.3048
3/7/2016	0.3093
3/8/2016	0.3160
3/9/2016	0.3047
3/10/2016	0.3050
3/11/2016	0.3111
3/12/2016	0.3097
3/13/2016	0.3150
3/14/2016	0.3125
3/15/2016	0.3112
3/16/2016	0.3149
3/17/2016	0.3128
3/18/2016	0.3125
3/19/2016	0.3095
3/20/2016	0.3116
3/21/2016	0.3114
3/22/2016	0.3122
3/23/2016	0.3157
3/24/2016	0.3104
3/25/2016	0.3105
3/26/2016	0.3116
3/27/2016	0.3133
3/28/2016	0.3092
3/29/2016	0.3122
3/30/2016	0.3080
3/31/2016	0.3108
4/1/2016	0.3093
4/2/2016	0.3144
4/3/2016	0.3148
4/4/2016	0.3171
4/5/2016	0.3157
4/6/2016	0.3143
4/7/2016	0.3166
4/8/2016	0.3162

4/9/2016	0.3194
4/10/2016	0.3186
4/11/2016	0.3165
4/12/2016	0.3154
4/13/2016	0.3215
4/14/2016	0.3240
4/15/2016	0.3235
4/16/2016	0.3192
4/17/2016	0.3193
4/18/2016	0.3267
4/19/2016	0.3198
4/20/2016	0.3225
4/21/2016	0.3239
4/22/2016	0.3244
4/23/2016	0.3238
4/24/2016	0.3252
4/25/2016	0.3304
4/26/2016	0.3292
4/27/2016	0.3263
4/28/2016	0.3303
4/29/2016	0.3295
4/30/2016	0.3351
5/1/2016	0.3301
5/2/2016	0.3385
5/3/2016	0.3341
5/4/2016	0.3281
5/5/2016	0.3338
5/6/2016	0.3337
5/7/2016	0.3332
5/8/2016	0.3324
5/9/2016	0.3335
5/10/2016	0.3358
5/11/2016	0.3321
5/12/2016	0.3346
5/13/2016	0.3383
5/14/2016	0.3397
5/15/2016	0.3391
5/16/2016	0.3389
5/17/2016	0.3405
5/18/2016	0.3406
5/19/2016	0.3413
5/20/2016	0.3360
5/21/2016	0.3333
5/22/2016	0.3422
5/23/2016	0.3370
5/24/2016	0.3390
5/25/2016	0.3409
5/26/2016	0.3401
5/27/2016	0.3425
5/28/2016	0.3398

5/29/2016	0.3396
5/30/2016	0.3382
5/31/2016	0.3411
6/1/2016	0.3400
6/2/2016	0.3465
6/3/2016	0.3420
6/4/2016	0.3367
6/5/2016	0.3432
6/6/2016	0.3410
6/7/2016	0.3457
6/8/2016	0.3388
6/9/2016	0.3429
6/10/2016	0.3415
6/11/2016	0.3414
6/12/2016	0.3443
6/13/2016	0.3479
6/14/2016	0.3467
6/15/2016	0.3410
6/16/2016	0.3480
6/17/2016	0.3438
6/18/2016	0.3485
6/19/2016	0.3461
6/20/2016	0.3423
6/21/2016	0.3404
6/22/2016	0.3450
6/23/2016	0.3485
6/24/2016	0.3487
6/25/2016	0.3392
6/26/2016	0.3398
6/27/2016	0.3513
6/28/2016	0.3447
6/29/2016	0.3485
6/30/2016	0.3460
7/1/2016	0.3404
7/2/2016	0.3475
7/3/2016	0.3496
7/4/2016	0.3485
7/5/2016	0.3512
7/6/2016	0.3414
7/7/2016	0.3510
7/8/2016	0.3529
7/9/2016	0.3519
7/10/2016	0.3529
7/11/2016	0.3535
7/12/2016	0.3488
7/13/2016	0.3523
7/14/2016	0.3651
7/15/2016	0.3541
7/16/2016	0.3573
7/17/2016	0.3561

7/18/2016	0.3577
7/19/2016	0.3565
7/20/2016	0.3625
7/21/2016	0.3630
7/22/2016	0.3556
7/23/2016	0.3575
7/24/2016	0.3599
7/25/2016	0.3541
7/26/2016	0.3552
7/27/2016	0.3603
7/28/2016	0.3548
7/29/2016	0.3645
7/30/2016	0.3566
7/31/2016	0.3549
8/1/2016	0.3518
8/31/2016	0.3609
9/1/2016	0.3572
9/2/2016	0.3642
9/3/2016	0.3604
9/4/2016	0.3638
9/5/2016	0.3646
9/6/2016	0.3616
9/7/2016	0.3634
9/8/2016	0.3661
9/9/2016	0.3643
9/10/2016	0.3606
9/11/2016	0.3624
9/12/2016	0.3664
9/13/2016	0.3557
9/14/2016	0.3631
9/15/2016	0.3598
9/16/2016	0.3682
9/17/2016	0.3582
9/18/2016	0.3653
9/19/2016	0.3601
9/20/2016	0.3657
9/21/2016	0.3615
9/22/2016	0.3625
9/23/2016	0.3629
9/24/2016	0.3540
9/25/2016	0.3674
9/26/2016	0.3620
9/27/2016	0.3607
9/28/2016	0.3634
9/29/2016	0.3616
9/30/2016	0.3578
10/1/2016	0.3607
10/2/2016	0.3622
10/3/2016	0.3587
10/4/2016	0.3657

10/5/2016	0.3606
10/6/2016	0.3723
10/7/2016	0.3597
10/8/2016	0.3695
10/9/2016	0.3640
10/10/2016	0.3630
10/11/2016	0.3647
10/12/2016	0.3646
10/13/2016	0.3668
10/14/2016	0.3652
10/15/2016	0.3645
10/16/2016	0.3650
10/17/2016	0.3622
10/18/2016	0.3681
10/19/2016	0.3639
10/20/2016	0.3651
10/21/2016	0.3693
10/22/2016	0.3670
10/23/2016	0.3657
10/24/2016	0.3669
10/25/2016	0.3689
10/26/2016	0.3704
10/27/2016	0.3674
10/28/2016	0.3766
10/29/2016	0.3660
10/30/2016	0.3730
10/31/2016	0.3747
11/1/2016	0.3668
11/2/2016	0.3724
11/3/2016	0.3699
11/4/2016	0.3675
11/5/2016	0.3747
11/6/2016	0.3715
11/7/2016	0.3728
11/8/2016	0.3730
11/9/2016	0.3747
11/10/2016	0.3690
11/11/2016	0.3732
11/12/2016	0.3659
11/13/2016	0.3705
11/14/2016	0.3703
11/15/2016	0.3717
11/16/2016	0.3692
11/17/2016	0.3678
11/18/2016	0.3704
11/19/2016	0.3679
11/20/2016	0.3651
11/21/2016	0.3670
11/22/2016	0.3687
11/23/2016	0.3677

11/24/2016	0.3732
11/25/2016	0.3719
11/26/2016	0.3757
11/27/2016	0.3699
11/28/2016	0.3714
11/29/2016	0.3706
11/30/2016	0.3732
12/1/2016	0.3667
12/2/2016	0.3691
12/3/2016	0.3683
12/4/2016	0.3700
12/6/2016	0.3712
12/7/2016	0.3691
12/8/2016	0.3693
12/9/2016	0.3701
12/10/2016	0.3695
12/11/2016	0.3716
12/12/2016	0.3741
12/13/2016	0.3713
12/14/2016	0.3691
12/15/2016	0.3713
12/16/2016	0.3731
12/17/2016	0.3700
12/18/2016	0.3689
12/19/2016	0.3766
12/20/2016	0.3710
12/21/2016	0.3733
12/22/2016	0.3702
12/23/2016	0.3736
12/24/2016	0.3745
12/25/2016	0.3706
12/26/2016	0.3742
12/27/2016	0.3757
12/28/2016	0.3725
12/29/2016	0.3759
12/30/2016	0.3802
12/31/2016	0.3715
1/1/2017	0.3743
1/2/2017	0.3773
1/3/2017	0.3728
1/4/2017	0.3740
1/5/2017	0.3790
1/6/2017	0.3783
1/7/2017	0.3704
1/8/2017	0.3797
1/9/2017	0.3761
1/10/2017	0.3729
1/11/2017	0.3695
1/12/2017	0.3705
1/13/2017	0.3787

1/14/2017	0.3733
1/15/2017	0.3763
1/16/2017	0.3761
1/17/2017	0.3787
1/18/2017	0.3730
1/19/2017	0.3740
1/20/2017	0.3705
1/21/2017	0.3726
1/22/2017	0.3735
1/23/2017	0.3719
1/24/2017	0.3747
1/25/2017	0.3724
1/26/2017	0.3680
1/27/2017	0.3696
1/28/2017	0.3738
1/29/2017	0.3729
1/30/2017	0.3685
1/31/2017	0.3738
2/1/2017	0.3720
2/2/2017	0.3698
2/3/2017	0.3733
2/4/2017	0.3705
2/5/2017	0.3710
2/8/2017	0.3728
2/9/2017	0.3744
2/10/2017	0.3725
2/11/2017	0.3719
2/12/2017	0.3688
2/13/2017	0.3680
2/14/2017	0.3732
2/15/2017	0.3721
2/16/2017	0.3727
2/17/2017	0.3739
2/18/2017	0.3710
2/19/2017	0.3714
2/20/2017	0.3713
2/21/2017	0.3724
2/22/2017	0.3748
2/23/2017	0.3728
2/24/2017	0.3739
2/25/2017	0.3711
2/26/2017	0.3729
2/27/2017	0.3741
2/28/2017	0.3734
3/1/2017	0.3682
3/2/2017	0.3726
3/3/2017	0.3737
3/4/2017	0.3723
3/5/2017	0.3760
3/6/2017	0.3736

3/7/2017	0.3789
3/8/2017	0.3761
3/9/2017	0.3756
3/10/2017	0.3826
3/11/2017	0.3780
3/12/2017	0.3751
3/13/2017	0.3722
3/14/2017	0.3728
3/15/2017	0.3763
3/16/2017	0.3748
3/17/2017	0.3769
3/18/2017	0.3751
3/19/2017	0.3755
3/20/2017	0.3766
3/21/2017	0.3770
3/22/2017	0.3738
3/23/2017	0.3743
3/24/2017	0.3775
3/25/2017	0.3719
3/26/2017	0.3746
3/27/2017	0.3764
3/28/2017	0.3772
3/29/2017	0.3751
3/30/2017	0.3760
3/31/2017	0.3775
4/1/2017	0.3779
4/2/2017	0.3768
4/3/2017	0.3717
4/4/2017	0.3824
4/5/2017	0.3815
4/6/2017	0.3818
4/7/2017	0.3773
4/8/2017	0.3784
4/9/2017	0.3807
4/10/2017	0.3802
4/11/2017	0.3786
4/12/2017	0.3810
4/13/2017	0.3811
4/14/2017	0.3804
4/15/2017	0.3796
4/16/2017	0.3761
4/17/2017	0.3809
4/18/2017	0.3864
4/19/2017	0.3803
4/20/2017	0.3812
4/21/2017	0.3786
4/22/2017	0.3798
4/23/2017	0.3811
4/24/2017	0.3797
4/25/2017	0.3772

4/26/2017	0.3740
4/27/2017	0.3799
4/28/2017	0.3846
4/29/2017	0.3801
4/30/2017	0.3802
5/1/2017	0.3784
5/2/2017	0.3797
5/4/2017	0.3814
5/5/2017	0.3797
5/6/2017	0.3831
5/7/2017	0.3791
5/8/2017	0.3790
5/9/2017	0.3767
5/10/2017	0.3750
5/11/2017	0.3758
5/12/2017	0.3780
5/13/2017	0.3765
5/14/2017	0.3736
5/15/2017	0.3792
5/16/2017	0.3866
5/17/2017	0.3805
5/18/2017	0.3848
5/19/2017	0.3757
5/20/2017	0.3759
5/21/2017	0.3791
5/22/2017	0.3816
5/23/2017	0.3756
5/24/2017	0.3811
5/25/2017	0.3800
5/26/2017	0.3826
5/27/2017	0.3779
5/28/2017	0.3850
5/29/2017	0.3786
5/30/2017	0.3785
5/31/2017	0.3721
6/1/2017	0.3847
6/2/2017	0.3878
6/3/2017	0.3705
6/4/2017	0.3778
6/5/2017	0.3782
6/6/2017	0.3727
6/7/2017	0.3784
6/8/2017	0.3818
6/9/2017	0.3829
6/10/2017	0.3765
6/11/2017	0.3815
6/12/2017	0.3836
6/13/2017	0.3852
6/14/2017	0.3839
6/15/2017	0.3736

6/16/2017	0.3830
6/17/2017	0.3886
6/18/2017	0.3817
6/19/2017	0.3834
6/20/2017	0.3833
6/21/2017	0.3779
6/22/2017	0.3838
6/23/2017	0.3832
6/24/2017	0.3783
6/25/2017	0.3772
6/26/2017	0.3789
6/27/2017	0.3799
6/28/2017	0.3907
6/29/2017	0.3816
6/30/2017	0.3804
7/1/2017	0.3843
7/2/2017	0.3831
7/3/2017	0.3840
7/4/2017	0.3892
7/5/2017	0.3866
7/6/2017	0.3830
7/7/2017	0.3880
7/8/2017	0.3839
7/9/2017	0.3869
7/10/2017	0.3861
7/11/2017	0.3861
7/12/2017	0.3865
7/13/2017	0.3878
7/14/2017	0.3913
7/15/2017	0.3775
7/16/2017	0.3849
7/17/2017	0.3859
7/18/2017	0.3858
7/19/2017	0.3852
7/20/2017	0.3888
7/21/2017	0.3844
7/22/2017	0.3863
7/23/2017	0.3910
7/24/2017	0.3866
7/25/2017	0.3852
7/26/2017	0.3845
7/27/2017	0.3962
7/28/2017	0.3923
7/29/2017	0.3876
7/30/2017	0.3919
7/31/2017	0.3932
8/1/2017	0.3895
8/2/2017	0.3870
8/3/2017	0.3861
8/4/2017	0.3864

8/5/2017	0.3907
8/6/2017	0.3881
8/7/2017	0.3905
8/8/2017	0.3994
8/9/2017	0.3932
8/11/2017	0.3927
8/12/2017	0.3870
8/13/2017	0.3942
8/14/2017	0.3912
8/15/2017	0.3939
8/16/2017	0.3999
8/17/2017	0.3917
8/18/2017	0.3859
8/19/2017	0.3979
8/20/2017	0.3952
8/21/2017	0.3974
8/22/2017	0.3985
8/23/2017	0.3968
8/24/2017	0.3945
8/25/2017	0.3972
8/26/2017	0.3977
8/27/2017	0.3956
8/28/2017	0.3988
8/29/2017	0.3953
8/30/2017	0.3981
8/31/2017	0.3886
9/1/2017	0.3940
9/2/2017	0.4027
9/3/2017	0.4003
9/4/2017	0.3929
9/5/2017	0.3970
9/6/2017	0.3977
9/7/2017	0.3974
9/8/2017	0.3885
9/9/2017	0.4063
9/10/2017	0.3905
9/11/2017	0.3975
9/12/2017	0.3931
9/13/2017	0.3938
9/14/2017	0.3957
9/15/2017	0.3937
9/16/2017	0.4009
9/17/2017	0.3923
9/18/2017	0.3961
9/19/2017	0.3937
9/20/2017	0.3952
9/21/2017	0.3929
9/22/2017	0.3975
9/23/2017	0.4013
9/24/2017	0.4003

9/25/2017	0.4065
9/26/2017	0.4038
9/27/2017	0.4003
9/28/2017	0.4015
9/29/2017	0.4027
9/30/2017	0.4013
10/1/2017	0.3953
10/2/2017	0.3976
10/3/2017	0.3985
10/4/2017	0.3987
10/5/2017	0.4023
10/6/2017	0.4067
10/7/2017	0.4037
10/8/2017	0.3973
10/9/2017	0.3993
10/10/2017	0.4008
10/11/2017	0.4029
10/12/2017	0.3993
10/13/2017	0.3974
10/14/2017	0.4029
10/15/2017	0.4016
10/16/2017	0.4004
10/17/2017	0.4023
10/18/2017	0.3950
10/19/2017	0.4006
10/20/2017	0.4019
10/21/2017	0.4032
10/22/2017	0.3956
10/23/2017	0.4027
10/24/2017	0.4067
10/25/2017	0.4096
10/26/2017	0.4051
10/27/2017	0.4060
10/28/2017	0.4112
10/29/2017	0.4096
10/30/2017	0.4104
10/31/2017	0.4078
11/1/2017	0.4075
11/2/2017	0.4106
11/3/2017	0.4062
11/4/2017	0.4062
11/5/2017	0.4125
11/6/2017	0.3994
11/7/2017	0.4101
11/8/2017	0.4091
11/9/2017	0.4118
11/10/2017	0.4116
11/11/2017	0.4092
11/12/2017	0.4103
11/13/2017	0.4116

11/14/2017	0.4092
11/15/2017	0.4133
11/16/2017	0.4112
11/17/2017	0.4099
11/18/2017	0.4139
11/19/2017	0.4143
11/20/2017	0.4130
11/21/2017	0.4141
11/22/2017	0.4143
11/23/2017	0.4084
11/24/2017	0.4119
11/25/2017	0.4147
11/26/2017	0.4166
11/27/2017	0.4128
11/28/2017	0.4130
11/29/2017	0.4106
11/30/2017	0.4155
12/1/2017	0.4150
12/2/2017	0.4200
12/3/2017	0.4124
12/4/2017	0.4158
12/5/2017	0.4179
12/6/2017	0.4159
12/7/2017	0.4179
12/8/2017	0.4185
12/9/2017	0.4242
12/10/2017	0.4200
12/11/2017	0.4156
12/12/2017	0.4218
12/13/2017	0.4188
12/14/2017	0.4168
12/15/2017	0.4197
12/16/2017	0.4181
12/17/2017	0.4277
12/18/2017	0.4215
12/19/2017	0.4250
12/20/2017	0.4236
12/21/2017	0.4252
12/22/2017	0.4208
12/23/2017	0.4238
12/24/2017	0.4244
12/25/2017	0.4203
12/26/2017	0.4247
12/27/2017	0.4294
12/28/2017	0.4246
12/29/2017	0.4188
12/30/2017	0.4216
12/31/2017	0.4222
1/1/2018	0.4286
1/2/2018	0.4228

1/3/2018	0.4243
1/4/2018	0.4228
1/5/2018	0.4208
1/6/2018	0.4179
1/7/2018	0.4231
1/8/2018	0.4316
1/9/2018	0.4291
1/10/2018	0.4294
1/11/2018	0.4204
1/12/2018	0.4271
1/13/2018	0.4275
1/14/2018	0.4230
1/15/2018	0.4270
1/16/2018	0.4270
1/17/2018	0.4331
1/18/2018	0.4253
1/19/2018	0.4336
1/20/2018	0.4263
1/21/2018	0.4292
1/22/2018	0.4318
1/23/2018	0.4327
1/24/2018	0.4313
1/25/2018	0.4309
1/26/2018	0.4316
1/27/2018	0.4323
1/28/2018	0.4323
1/29/2018	0.4354
1/30/2018	0.4338
1/31/2018	0.4354
2/1/2018	0.4391
2/2/2018	0.4447
2/3/2018	0.4363
2/4/2018	0.4370
2/5/2018	0.4378
2/6/2018	0.4356
2/7/2018	0.4374
2/8/2018	0.4389
2/9/2018	0.4358
2/10/2018	0.4415
2/11/2018	0.4373
2/12/2018	0.4377
2/13/2018	0.4375
2/14/2018	0.4369
2/15/2018	0.4396
2/16/2018	0.4394
2/17/2018	0.4374
2/18/2018	0.4422
2/19/2018	0.4415
2/20/2018	0.4407
2/21/2018	0.4420

2/22/2018	0.4414
2/23/2018	0.4360
2/24/2018	0.4361
2/25/2018	0.4412
2/26/2018	0.4440
2/27/2018	0.4409
2/28/2018	0.4391
3/1/2018	0.4513
3/2/2018	0.4423
3/3/2018	0.4420
3/4/2018	0.4383
3/5/2018	0.4304
3/6/2018	0.4353
3/7/2018	0.4343
3/8/2018	0.4405
3/9/2018	0.4373
3/10/2018	0.4409
3/11/2018	0.4411
3/12/2018	0.4430
3/13/2018	0.4370
3/14/2018	0.4392
3/15/2018	0.4393
3/16/2018	0.4404
3/17/2018	0.4459
3/18/2018	0.4468
3/20/2018	0.4433
3/22/2018	0.4512
3/23/2018	0.4452
3/24/2018	0.4442
3/25/2018	0.4454
3/26/2018	0.4477
3/27/2018	0.4478
3/28/2018	0.4451
3/29/2018	0.4486
3/30/2018	0.4484
3/31/2018	0.4524
4/1/2018	0.4508
4/2/2018	0.4481
4/3/2018	0.4479
4/4/2018	0.4519
4/5/2018	0.4510
4/6/2018	0.4488
4/7/2018	0.4531
4/8/2018	0.4510
4/9/2018	0.4504
4/10/2018	0.4500
4/11/2018	0.4489
4/12/2018	0.4262
4/13/2018	0.4373
4/14/2018	0.4574

4/15/2018	0.4555
4/16/2018	0.4550
4/17/2018	0.4553
4/18/2018	0.4592
4/19/2018	0.4534
4/21/2018	0.4539
4/22/2018	0.4540
4/23/2018	0.4514
4/24/2018	0.4554
4/25/2018	0.4512
4/26/2018	0.4529
4/27/2018	0.4513
4/28/2018	0.4553
4/29/2018	0.4549
4/30/2018	0.4587
5/1/2018	0.4620
5/2/2018	0.4613
5/3/2018	0.4568
5/4/2018	0.4627
5/5/2018	0.4588
5/6/2018	0.4578
5/7/2018	0.4526
5/8/2018	0.4614
5/9/2018	0.4546
5/10/2018	0.4557
5/11/2018	0.4540
5/12/2018	0.4606
5/13/2018	0.4533
5/14/2018	0.4576
5/15/2018	0.4592
5/16/2018	0.4646
5/17/2018	0.4576
5/18/2018	0.4616
5/19/2018	0.4614
5/20/2018	0.4622
5/21/2018	0.4580
5/22/2018	0.4586
5/23/2018	0.4623
5/24/2018	0.4559
5/25/2018	0.4687
5/26/2018	0.4665
5/27/2018	0.4592
5/28/2018	0.4619
5/29/2018	0.4601
5/30/2018	0.4605
5/31/2018	0.4689
6/1/2018	0.4639
6/2/2018	0.4679
6/3/2018	0.4684
6/4/2018	0.4673

6/5/2018	0.4715
6/6/2018	0.4630
6/7/2018	0.4717
6/8/2018	0.4702
6/9/2018	0.4676
6/10/2018	0.4676
6/11/2018	0.4676
6/12/2018	0.4639
6/13/2018	0.4664
6/14/2018	0.4748
6/15/2018	0.4716
6/16/2018	0.4703
6/17/2018	0.4682
6/18/2018	0.4692
6/19/2018	0.4790
6/20/2018	0.4691
6/21/2018	0.4666
6/22/2018	0.4744
6/23/2018	0.4746
6/24/2018	0.4761
6/25/2018	0.4768
6/26/2018	0.4733
6/27/2018	0.4739
6/28/2018	0.4805
6/29/2018	0.4682
6/30/2018	0.4697
7/1/2018	0.4750
7/2/2018	0.4714
7/3/2018	0.4789
7/4/2018	0.4738
7/5/2018	0.4717
7/6/2018	0.4755
7/7/2018	0.4796
7/8/2018	0.4797
7/9/2018	0.4816
7/10/2018	0.4815
7/11/2018	0.4771
7/12/2018	0.4721
7/13/2018	0.4772
7/14/2018	0.4794
7/15/2018	0.4791
7/16/2018	0.4751
7/17/2018	0.4824
7/18/2018	0.4731
7/19/2018	0.4771
7/20/2018	0.4739
7/21/2018	0.4748
7/22/2018	0.4812
7/23/2018	0.4773
7/24/2018	0.4784

7/25/2018	0.4789
7/26/2018	0.4705
7/27/2018	0.4839
7/28/2018	0.4726
7/29/2018	0.4815
7/30/2018	0.4826
7/31/2018	0.4775
8/1/2018	0.4825
8/2/2018	0.4833
8/3/2018	0.4819
8/4/2018	0.4772
8/5/2018	0.4837
8/6/2018	0.4784
8/7/2018	0.4738
8/8/2018	0.4843
8/9/2018	0.4758
8/10/2018	0.4745
8/11/2018	0.4805
8/12/2018	0.4735
8/13/2018	0.4787
8/14/2018	0.4877
8/15/2018	0.4796
8/16/2018	0.4957
8/17/2018	0.4856
8/18/2018	0.4814
8/19/2018	0.4821
8/20/2018	0.4841
8/21/2018	0.4857
8/22/2018	0.4808
8/23/2018	0.4827
8/24/2018	0.4743
8/25/2018	0.4897
8/26/2018	0.4626
8/27/2018	0.4844
8/28/2018	0.4832
8/29/2018	0.4860
8/30/2018	0.4816
8/31/2018	0.4832
9/1/2018	0.4837
9/2/2018	0.4893
9/3/2018	0.4840
9/4/2018	0.4893
9/5/2018	0.4883
9/6/2018	0.4896
9/7/2018	0.4859
9/8/2018	0.4876
9/9/2018	0.4900
9/10/2018	0.4844
9/11/2018	0.4898
9/12/2018	0.4863

9/13/2018	0.4884
9/14/2018	0.4955
9/15/2018	0.4906
9/16/2018	0.4906
9/17/2018	0.4786
9/18/2018	0.4850
9/19/2018	0.4908
9/20/2018	0.4940
9/21/2018	0.4897
9/22/2018	0.4899
9/23/2018	0.4902
9/24/2018	0.4942
9/25/2018	0.4984
9/26/2018	0.4927
9/27/2018	0.4948
9/28/2018	0.4980
9/29/2018	0.4923
9/30/2018	0.4944
10/1/2018	0.5147
10/2/2018	0.5019
10/3/2018	0.4972
10/4/2018	0.4890
10/5/2018	0.4916
10/6/2018	0.4833
10/7/2018	0.4933
10/8/2018	0.4943
10/9/2018	0.4925
10/10/2018	0.4937
10/11/2018	0.4941
10/12/2018	0.4981
10/13/2018	0.4975
10/14/2018	0.4916
10/15/2018	0.4989
10/16/2018	0.4978
10/17/2018	0.4992
10/18/2018	0.4979
10/19/2018	0.4985
10/20/2018	0.4976
10/21/2018	0.4981
10/22/2018	0.4989
10/23/2018	0.5042
10/24/2018	0.5005
10/25/2018	0.5013
10/26/2018	0.4999
10/27/2018	0.4941
10/28/2018	0.5024
10/29/2018	0.4983
10/30/2018	0.4989
10/31/2018	0.4999
11/1/2018	0.4967

11/2/2018	0.5039
11/3/2018	0.4956
11/4/2018	0.5043
11/5/2018	0.5002
11/6/2018	0.4966
11/7/2018	0.4993
11/8/2018	0.4964
11/9/2018	0.5007
11/10/2018	0.4985
11/11/2018	0.4990
11/12/2018	0.5028
11/13/2018	0.4963
11/14/2018	0.4987
11/15/2018	0.4987
11/16/2018	0.5028
11/17/2018	0.5033
11/18/2018	0.4989
11/19/2018	0.5061
11/20/2018	0.4975
11/21/2018	0.5020
11/22/2018	0.4915
11/23/2018	0.4952
11/24/2018	0.5004
11/25/2018	0.5052
11/26/2018	0.5008
11/27/2018	0.5101
11/28/2018	0.5052
11/29/2018	0.5072
11/30/2018	0.5022
12/1/2018	0.5053
12/2/2018	0.5013
12/3/2018	0.4998
12/4/2018	0.5069
12/5/2018	0.5080
12/6/2018	0.4984
12/7/2018	0.5043
12/8/2018	0.5017
12/9/2018	0.5074
12/10/2018	0.5060
12/11/2018	0.5045
12/12/2018	0.5029
12/13/2018	0.5061
12/14/2018	0.5072
12/15/2018	0.5070
12/16/2018	0.5042
12/17/2018	0.5064
12/18/2018	0.5053
12/19/2018	0.5048
12/20/2018	0.5050
12/21/2018	0.5056

12/22/2018	0.5060
12/23/2018	0.5086
12/24/2018	0.5094
12/25/2018	0.5105
12/26/2018	0.5116
12/27/2018	0.5072
12/28/2018	0.5080
12/29/2018	0.5106
12/30/2018	0.5094
12/31/2018	0.5096
1/1/2019	0.5090
1/2/2019	0.5117
1/3/2019	0.5122
1/4/2019	0.5090
1/5/2019	0.5123
1/6/2019	0.5167
1/7/2019	0.5113
1/8/2019	0.5109
1/9/2019	0.5119
1/10/2019	0.5163
1/11/2019	0.5168
1/12/2019	0.5169
1/13/2019	0.5104
1/14/2019	0.5139
1/15/2019	0.5133
1/16/2019	0.5099
1/17/2019	0.5126
1/18/2019	0.5111
1/19/2019	0.5138
1/20/2019	0.5128
1/21/2019	0.5121
1/22/2019	0.5150
1/23/2019	0.5141
1/24/2019	0.5158
1/25/2019	0.5138
1/26/2019	0.5116
1/27/2019	0.5154
1/28/2019	0.5134
1/29/2019	0.5117
1/30/2019	0.5145
1/31/2019	0.5121
2/1/2019	0.5178
2/2/2019	0.5144
2/3/2019	0.5193
2/4/2019	0.5194
2/5/2019	0.5185
2/6/2019	0.5196
2/7/2019	0.5160
2/8/2019	0.5178
2/9/2019	0.5136

2/10/2019	0.5149
2/11/2019	0.5232
2/12/2019	0.5223
2/13/2019	0.5181
2/14/2019	0.5229
2/15/2019	0.5274
2/16/2019	0.5207
2/17/2019	0.5173
2/18/2019	0.5210
2/19/2019	0.5202
2/20/2019	0.5212
2/21/2019	0.5208
2/22/2019	0.5178
2/23/2019	0.5325
2/24/2019	0.5259
2/25/2019	0.5233
2/26/2019	0.5273
2/27/2019	0.5285
2/28/2019	0.5266
3/1/2019	0.5316
3/2/2019	0.5273
3/3/2019	0.5247
3/4/2019	0.5272
3/5/2019	0.5233
3/6/2019	0.5211
3/7/2019	0.5269
3/8/2019	0.5232
3/9/2019	0.5263
3/10/2019	0.5277
3/11/2019	0.5288
3/12/2019	0.5276
3/13/2019	0.5301
3/14/2019	0.5319
3/15/2019	0.5263
3/16/2019	0.5275
3/17/2019	0.5322
3/18/2019	0.5309
3/19/2019	0.5320
3/20/2019	0.5351
3/21/2019	0.5348
3/22/2019	0.5410
3/23/2019	0.5351
3/24/2019	0.5329
3/25/2019	0.5369
3/26/2019	0.5385
3/27/2019	0.5382
3/28/2019	0.5416
3/29/2019	0.5335
3/30/2019	0.5322
3/31/2019	0.5360

4/1/2019	0.5356
4/2/2019	0.5327
4/3/2019	0.5315
4/4/2019	0.5266
4/5/2019	0.5316
4/6/2019	0.5359
4/7/2019	0.5386
4/8/2019	0.5365
4/9/2019	0.5328
4/10/2019	0.5340
4/11/2019	0.5405
4/12/2019	0.5390
4/13/2019	0.5364
4/14/2019	0.5376
4/15/2019	0.5339
4/16/2019	0.5388
4/17/2019	0.5395
4/18/2019	0.5360
4/19/2019	0.5382
4/20/2019	0.5389
4/21/2019	0.5378
4/22/2019	0.5400
4/23/2019	0.5400
4/24/2019	0.5388
4/25/2019	0.5383
4/26/2019	0.5370
4/27/2019	0.5440
4/28/2019	0.5427
4/29/2019	0.5421
4/30/2019	0.5463
5/1/2019	0.5398
5/2/2019	0.5402
5/3/2019	0.5426
5/4/2019	0.5461
5/5/2019	0.5494
5/6/2019	0.5438
5/7/2019	0.5422
5/8/2019	0.5419
5/9/2019	0.5437
5/10/2019	0.5423
5/11/2019	0.5457
5/12/2019	0.5433
5/13/2019	0.5495
5/14/2019	0.5539
5/15/2019	0.5469
5/16/2019	0.5473
5/17/2019	0.5501
5/18/2019	0.5481
5/19/2019	0.5465
5/20/2019	0.5474

5/21/2019	0.5497
5/22/2019	0.5431
5/23/2019	0.5480
5/24/2019	0.5490
5/25/2019	0.5505
5/26/2019	0.5497
5/27/2019	0.5467
5/28/2019	0.5531
5/29/2019	0.5530
5/30/2019	0.5585
5/31/2019	0.5540
6/1/2019	0.5563
6/2/2019	0.5557
6/3/2019	0.5520
6/4/2019	0.5525
6/5/2019	0.5524
6/6/2019	0.5536
6/7/2019	0.5547
6/8/2019	0.5540
6/9/2019	0.5563
6/10/2019	0.5604
6/11/2019	0.5577
6/12/2019	0.5586
6/13/2019	0.5556
6/14/2019	0.5561
6/16/2019	0.5588
6/17/2019	0.5592
6/18/2019	0.5547
6/19/2019	0.5495
6/20/2019	0.5617
6/21/2019	0.5597
6/22/2019	0.5620
6/23/2019	0.5542
6/24/2019	0.5575
6/25/2019	0.5627
6/26/2019	0.5616
6/27/2019	0.5669
6/28/2019	0.5654
6/29/2019	0.5593
6/30/2019	0.5604
7/1/2019	0.5598
7/2/2019	0.5629
7/3/2019	0.5615
7/4/2019	0.5681
7/5/2019	0.5587
7/6/2019	0.5567
7/7/2019	0.5598
7/8/2019	0.5680
7/9/2019	0.5626
7/10/2019	0.5632

7/11/2019	0.5661
7/12/2019	0.5693
7/13/2019	0.5624
7/14/2019	0.5718
7/15/2019	0.5672
7/16/2019	0.5661
7/17/2019	0.5669
7/18/2019	0.5633
7/19/2019	0.5678
7/20/2019	0.5626
7/21/2019	0.5649
7/22/2019	0.5703
7/23/2019	0.5764
7/24/2019	0.5616
7/25/2019	0.5655
7/26/2019	0.5634
7/27/2019	0.5733
7/28/2019	0.5740
7/29/2019	0.5684
7/30/2019	0.5680
7/31/2019	0.5580
8/1/2019	0.5676
8/2/2019	0.5695
8/3/2019	0.5696
8/4/2019	0.5633
8/5/2019	0.5694
8/6/2019	0.5654
8/7/2019	0.5723
8/8/2019	0.5745
8/9/2019	0.5721
8/10/2019	0.5687
8/11/2019	0.5789
8/12/2019	0.5743
8/13/2019	0.5767
8/14/2019	0.5877
8/15/2019	0.5786
8/16/2019	0.5806
8/17/2019	0.5762
8/18/2019	0.5680
8/19/2019	0.5755
8/20/2019	0.5738
8/21/2019	0.5818
8/22/2019	0.5753
8/23/2019	0.5861
8/24/2019	0.5758
8/25/2019	0.5802
8/26/2019	0.5800
8/27/2019	0.5755
8/28/2019	0.5797
8/29/2019	0.5830

8/30/2019	0.5784
8/31/2019	0.5799
9/1/2019	0.5845
9/2/2019	0.5865
9/3/2019	0.5743
9/4/2019	0.5826
9/5/2019	0.5815
9/6/2019	0.5882
9/7/2019	0.5860
9/8/2019	0.5808
9/9/2019	0.5780
9/10/2019	0.5798
9/11/2019	0.5811
9/12/2019	0.5777
9/13/2019	0.5838
9/14/2019	0.5827
9/15/2019	0.5830
9/16/2019	0.5781
9/17/2019	0.5812
9/18/2019	0.5830
9/19/2019	0.5869
9/20/2019	0.5860
9/21/2019	0.5838
9/22/2019	0.5831
9/23/2019	0.5776
9/24/2019	0.5813
9/25/2019	0.5813
9/26/2019	0.5859
9/27/2019	0.5820
9/28/2019	0.5840
9/29/2019	0.5860
9/30/2019	0.5865
10/1/2019	0.5834
10/2/2019	0.5940
10/3/2019	0.5939
10/4/2019	0.5847
10/5/2019	0.5885
10/6/2019	0.5897
10/7/2019	0.5887
10/8/2019	0.5834
10/9/2019	0.5868
10/10/2019	0.5872
10/11/2019	0.5849
10/12/2019	0.5806
10/13/2019	0.5833
10/14/2019	0.5799
10/15/2019	0.5832
10/16/2019	0.5864
10/17/2019	0.5870
10/18/2019	0.5818

10/19/2019	0.5874
10/20/2019	0.5871
10/21/2019	0.5893
10/22/2019	0.5883
10/23/2019	0.5894
10/24/2019	0.5898
10/25/2019	0.5971
10/26/2019	0.5898
10/27/2019	0.5939
10/28/2019	0.5875
10/29/2019	0.5877
10/30/2019	0.5898
10/31/2019	0.5923
11/1/2019	0.5948
11/2/2019	0.5984
11/3/2019	0.5981
11/4/2019	0.5916
11/5/2019	0.5953
11/6/2019	0.5907
11/7/2019	0.5960
11/8/2019	0.5981
11/9/2019	0.5945
11/10/2019	0.5878
11/11/2019	0.6017
11/12/2019	0.5998
11/13/2019	0.5969
11/14/2019	0.5920
11/15/2019	0.6016
11/16/2019	0.6053
11/17/2019	0.6067
11/19/2019	0.6055
11/20/2019	0.5992
11/21/2019	0.5949
11/22/2019	0.6004
11/23/2019	0.5986
11/24/2019	0.6029
11/25/2019	0.6109
11/26/2019	0.6080
11/27/2019	0.6082
11/28/2019	0.5997
11/29/2019	0.6112
11/30/2019	0.6129
12/1/2019	0.6115
12/2/2019	0.6117
12/3/2019	0.6112
12/4/2019	0.6099
12/5/2019	0.6099
12/6/2019	0.6113
12/7/2019	0.6059
12/8/2019	0.6105

12/9/2019	0.6138
12/10/2019	0.6161
12/11/2019	0.6150
12/13/2019	0.6121
12/14/2019	0.6177
12/15/2019	0.6117
12/16/2019	0.6120
12/17/2019	0.6111
12/18/2019	0.6071
12/19/2019	0.6121
12/20/2019	0.6087
12/21/2019	0.6152
12/22/2019	0.6143
12/23/2019	0.6171
12/24/2019	0.6145
12/25/2019	0.6127
12/26/2019	0.6157
12/27/2019	0.6141
12/28/2019	0.6117
12/29/2019	0.6205
12/30/2019	0.6205
12/31/2019	0.6090
1/1/2020	0.6142
1/2/2020	0.6119
1/3/2020	0.6144
1/4/2020	0.6140
1/5/2020	0.6185
1/6/2020	0.6178
1/7/2020	0.6143
1/8/2020	0.6141
1/9/2020	0.6143
1/10/2020	0.6156
1/11/2020	0.6168
1/12/2020	0.6141
1/13/2020	0.6167
1/14/2020	0.6144
1/15/2020	0.6165
1/16/2020	0.6176
1/17/2020	0.6178
1/18/2020	0.6182
1/19/2020	0.6178
1/20/2020	0.6202
1/21/2020	0.6178
1/22/2020	0.6183
1/23/2020	0.6215
1/24/2020	0.6204
1/25/2020	0.6206
1/26/2020	0.6234
1/27/2020	0.6196
1/28/2020	0.6201

1/29/2020	0.6244
1/30/2020	0.6171
1/31/2020	0.6175
2/1/2020	0.6195
2/2/2020	0.6184
2/3/2020	0.6209
2/4/2020	0.6234
2/5/2020	0.6224
2/6/2020	0.6229
2/7/2020	0.6227
2/8/2020	0.6212
2/9/2020	0.6202
2/10/2020	0.6178
2/11/2020	0.6245
2/12/2020	0.6223
2/13/2020	0.6232
2/14/2020	0.6222
2/15/2020	0.6215
2/16/2020	0.6199
2/17/2020	0.6218
2/18/2020	0.6259
2/19/2020	0.6228
2/20/2020	0.6229
2/21/2020	0.6263
2/22/2020	0.6302
2/23/2020	0.6253
2/24/2020	0.6268
2/25/2020	0.6261
2/26/2020	0.6251
2/27/2020	0.6264
2/28/2020	0.6278
2/29/2020	0.6254
3/1/2020	0.6312
3/2/2020	0.6256
3/3/2020	0.6289
3/4/2020	0.6272
3/5/2020	0.6243
3/6/2020	0.6226
3/7/2020	0.6321
3/8/2020	0.6285
3/9/2020	0.6247
3/10/2020	0.6294
3/11/2020	0.6262
3/12/2020	0.6272
3/13/2020	0.6281
3/14/2020	0.6260
3/15/2020	0.6283
3/16/2020	0.6265
3/17/2020	0.6310
3/18/2020	0.6313

3/19/2020	0.6265
3/20/2020	0.6292
3/21/2020	0.6319
3/22/2020	0.6339
3/23/2020	0.6372
3/24/2020	0.6328
3/25/2020	0.6347
3/26/2020	0.6315
3/27/2020	0.6305
3/28/2020	0.6280
3/29/2020	0.6315
3/30/2020	0.6277
3/31/2020	0.6276
4/1/2020	0.6317
4/2/2020	0.6260
4/3/2020	0.6312
4/4/2020	0.6314
4/5/2020	0.6291
4/6/2020	0.6320
4/7/2020	0.6352
4/8/2020	0.6322
4/9/2020	0.6319
4/10/2020	0.6312
4/11/2020	0.6346
4/12/2020	0.6322
4/13/2020	0.6326
4/14/2020	0.6376
4/15/2020	0.6346
4/16/2020	0.6281
4/17/2020	0.6321
4/18/2020	0.6331
4/19/2020	0.6321
4/20/2020	0.6330
4/21/2020	0.6354
4/22/2020	0.6335
4/23/2020	0.6332
4/24/2020	0.6333
4/25/2020	0.6297
4/26/2020	0.6302
4/27/2020	0.6289
4/28/2020	0.6329
4/29/2020	0.6327
4/30/2020	0.6324
5/1/2020	0.6348
5/2/2020	0.6334
5/3/2020	0.6380
5/4/2020	0.6307
5/5/2020	0.6359
5/6/2020	0.6335
5/7/2020	0.6380

5/8/2020	0.6318
5/9/2020	0.6337
5/10/2020	0.6344
5/11/2020	0.6329
5/12/2020	0.6325
5/13/2020	0.6398
5/14/2020	0.6371
5/15/2020	0.6381
5/16/2020	0.6401
5/17/2020	0.6428
5/18/2020	0.6416
5/19/2020	0.6360
5/20/2020	0.6392
5/21/2020	0.6370
5/22/2020	0.6448
5/23/2020	0.6380
5/24/2020	0.6389
5/25/2020	0.6427
5/26/2020	0.6419
5/27/2020	0.6476
5/28/2020	0.6421
5/29/2020	0.6418
5/30/2020	0.6418
5/31/2020	0.6405
6/1/2020	0.6444
6/2/2020	0.6422
6/3/2020	0.6428
6/4/2020	0.6425
6/5/2020	0.6437
6/6/2020	0.6410
6/7/2020	0.6355
6/8/2020	0.6405
6/9/2020	0.6481
6/10/2020	0.6360
6/11/2020	0.6398
6/12/2020	0.6406
6/13/2020	0.6431
6/14/2020	0.6412
6/15/2020	0.6434
6/16/2020	0.6469
6/17/2020	0.6357
6/18/2020	0.6404
6/19/2020	0.6502
6/20/2020	0.6448
6/21/2020	0.6500
6/22/2020	0.6514
6/23/2020	0.6524
6/24/2020	0.6496
6/25/2020	0.6490
6/26/2020	0.6526

6/27/2020	0.6484
6/28/2020	0.6512
6/29/2020	0.6419
6/30/2020	0.6464
